# Two-electron two-nucleus effective Hamiltonian and the spin diffusion barrier

**DOI:** 10.1126/sciadv.adr7168

**Published:** 2025-01-03

**Authors:** Gevin von Witte, Sebastian Kozerke, Matthias Ernst

**Affiliations:** ^1^ Institute for Biomedical Engineering, University and ETH Zurich, 8092 Zurich, Switzerland.; ^2^ Institute of Molecular Physical Science, ETH Zurich, 8093 Zurich, Switzerland.

## Abstract

Dynamic nuclear polarization (DNP) and emerging quantum technologies rely on the spin transfer in electron-nuclear hybrid quantum systems. Spin transfers might be suppressed by larger couplings, e.g., hyperfine couplings suppressing nuclear dipolar flip-flops (”spin diffusion barrier”). We apply the Schrieffer-Wolff transformation to a two-electron two-nucleus spin system involving dipolar and hyperfine couplings in their tensorial form and study possible polarization-transfer processes. Among the different effective Hamiltonian matrix elements investigated is an energy-conserving electron-nuclear four-spin flip-flop, which combines an electronic with a nuclear dipolar flip-flop. The relevance of this electron-nuclear four-spin flip-flop for nuclear spin diffusion close to electrons is supported by model fits of HypRes-on experimental data. We connect the closely related fields of magnetic resonance and quantum information and provide a model that explains how all nuclear spins can contribute to the hyperpolarization of the bulk without a spin diffusion barrier in DNP.

## Teaser

Combined nuclear-electron transitions in four-spin systems can explain spin diffusion close to the electrons.

## INTRODUCTION

In nuclear magnetic resonance (NMR), the problem of interactions between electron and nuclear spins has been discussed since at least the 1940s (*1*–*13*). Dynamic nuclear polarization (DNP) relies on the large thermal polarization and fast relaxation of unpaired electrons for transfer to low thermal polarization baths, typically slow relaxing nuclear spins. Microwave (MW) irradiation transfers polarization from the unpaired electrons (often called radicals in DNP) to hyperfine-coupled nuclei. In DNP, the polarization transfer efficiency is limited not only by the polarization transfer from electrons to nuclei but also by the subsequent transport of the nuclear hyperpolarization into the bulk. Typically, nuclear spins in the bulk of the sample can be observed with inductive detection following a radio frequency (RF) excitation pulse. While hyperfine-coupled spins show the most efficient polarization transfer, they are strongly frequency shifted rendering them often unobservable in NMR (quenched, hidden or hypershifted spins). Accordingly, dipolar nuclear spin flip-flop processes are non-energy conserving. Nuclear spin flip-flops, described macroscopically by a nuclear spin diffusion rate constant, subsequently spread the transferred polarization (homogeneously) throughout the sample. Throughout this work, we will refer to the recently coined term hypershifted spins (*10*) to relate to strongly hyperfine-coupled spins that are difficult to observe with RF pulses.

In 1949, Bloembergen proposed the concept of a ‘spin diffusion barrier’ (*1*), describing spins that are strongly coupled to unpaired electrons and, therefore, frequency shifted (hypershifted), such that they do not contribute to spin diffusion toward the bulk. $T_{1,e}$ relaxation of the electrons will lead to a broadening of the hyperfine-split lines and eventually, for fast $T_{1,e}$ times to a population-averaged pseudo-contact shift (*14*–*17*) that can be substantial under DNP conditions because the polarization of the electrons will be high at low temperatures and high fields. Several experiments have demonstrated indirectly (*4*, *6*, *8*, *9*, *18*, *19*) or directly (*10*) an effective contribution of spins assumed to be within the spin diffusion barrier to the DNP process. These studies may question the size and existence of a spin diffusion barrier. Theoretical works aimed to explain these through relaxation processes, i.e. paramagnetic (electronic) (*3*, *9*, *20*–*22*) or nuclear (*23*) relaxation causing a nuclear-nuclear flip-flop. In addition, the broadening of the zero-quantum (ZQ; nuclear flip-flop) line by the electron has been proposed as another pathway to make the spin diffusion close to electrons more efficient (*5*). These models yield a strongly suppressed (vanishing) spin diffusion rate constant for spins less than several Angstrom away from the electron and a spin diffusion rate constant always smaller or equal to the one in the bulk. In contrast, simulations of quantum dots suggest a spin diffusion coefficient around the electron exceeding its bulk value (*24*) attributed to electron-mediated nuclear flip-flops described as two virtual electron-nuclear flip-flops (*25*). In a similar direction, spin diffusion close to pairs of P1 centers in diamond is discussed in terms of two virtual electron-electron-nuclear triple spin flips (*26*, *27*).

For materials with a large electron line width and limited electron dipolar coupling, MW irradiation at a given frequency results in a hole burned into the electron spectrum (*28*). The resulting polarization difference between the hole and the rest of the electrons unaffected by the MW can be used to perform cross-effect (CE) DNP. The minimum model to understand CE DNP consists of two electrons and a nucleus (*29*). If the frequency difference between the electrons $\Delta \omega_{e}=\omega_{e,1}-\omega_{e,2}$ becomes equal to the frequency of the nuclear Larmor frequency $\omega_{n}$ (CE condition: $\Delta \omega_{e}\approx \pm \omega_{n}$), then MW irradiation results in an efficient polarization transfer. Thus, the fundamental process to generate hyperpolarization is a three-spin flip-flop-flip with an electronic flip-flop and a nuclear flip.

In the past decades, condensed matter systems have developed into one of the prime approaches for quantum information processing thanks to advanced manufacturing technology and tunability (*30*). Defect centers in crystals such as the nitrogen-vacancy (NV) center in diamond, phosphorous (P) dopants in silicon or quantum dots consist of a single or multiple unbound electrons surrounded by nuclear spins of the host crystal. Electron spins offer faster gate times and easier readout at the expense of a shorter qubit coherence time ($T_{2}$). The opposite is true for nuclear spins. This has inspired the use of hybrid electron-nuclear spin systems with nuclear spins for processing or as a long coherence time qubit memory, which is read out through an electron (*31*–*35*). In either case of a hybrid electron-nuclear spin system, the coherence times of the electron and nuclear spins are highly dependent on the interactions between the two spin types. Even if only the electron spin is used for a specific application, the interaction with background nuclear spins, e.g., ^13^C or ^29^Si with 1.1 and 4.7% natural abundance, strongly influence the electron’s relaxation (*36*). Hence, isotope control, i.e., host crystals containing only a reduced or vanishing amount of nuclear isotopes with a magnetic moment, represents an efficient strategy to prolong electron coherence times (*37*–*41*). Similar relaxation dependencies between electrons and nuclei have been studied in NMR, electron paramagnetic resonance (EPR) and DNP. In NMR and DNP, nuclear relaxation by nearby electrons (paramagnetic relaxation) has been studied extensively (*17*). In addition, the shortening effects of nuclear fluctuations, e.g., nuclear flip-flops, often called nuclear spin diffusion, reorientation of chemical (methyl) groups, or tunneling, on the electronic phase memory or coherence times have been investigated in EPR (*42*–*46*).

The present work is divided in two parts: We first study a four-spin model consisting of two electrons and two nuclei to describe possible spin-transfer processes near a defect center (electron) before discussing different spin transport mechanisms in DNP. In the first part, we apply a lowest-order Schrieffer-Wolff transformation (*47*) to the spin system to calculate an effective Hamiltonian describing the electron-nuclear spin dynamics. The chosen approach involving the Schrieffer-Wolff transformation is widely used in quantum many-body systems (*47*) and enables the efficient generation of effective Hamiltonians of spin systems with computer algebra systems, e.g., Mathematica (Wolfram Research, USA) as demonstrated in section S7. The use of computer algebra systems enables the study of larger spin systems than can typically be on paper, e.g., more than two to three spins, and provides the effective transition-matrix elements of different process. In the following, we choose a didactic approach in showcasing that the lowest-order Schrieffer-Wolff transformation in combination with the two-electron two-nucleus spin system can recover several known spin-transfer processes and an electron-nuclear four-spin flip-flop potentially mediating nuclear spin diffusion close to paramagnetic defects. In the second part, to study if electron-nuclear four-spin flip-flops could explain recent experimental evidence of spin diffusion between hypershifted and bulk nuclei, we simulate HypRes-on experimental data (*9*). To this end, the previously introduced one-compartment model of hyperpolarization (*48*, *49*) is extended to two coupled compartments (Sec. S3, Supplementary Material). Simulation results suggest similar scaling of DNP injection by triple spin flips and inter-compartment coupling with applied MW power in agreement with the hypothesis that electron-nuclear four-spin flip-flops in DNP cause spin transport from hypershifted to bulk nuclei. Therefore, the studied electron-nuclear four-spin flip-flops provide a theoretical foundation for the experimentally observed absence of a spin diffusion barrier (*4*, *6*, *8*–*10*, *18*, *19*).

## RESULTS

### Two-electron two-nucleus spin system

The Hamiltonian of a two-electron two-nucleus spin system in the laboratory frame assuming identical frequencies for the two nuclei $\left(\omega_{n,1}=\omega_{n,2}=\omega_{n}\right)$ and allowing for different electron frequencies (i.e., due to g-anisotropy or two different radicals; $\omega_{e,a}=\omega_{e}+\Delta \omega_{e,a}$ and $\omega_{e,b}=\omega_{e}+\Delta \omega_{e,b}$) is given by

$$\begin{array}{c} H=\omega_{e,a}S_{a}^{z}+\omega_{e,b}S_{b}^{z}+{\vec{S}}_{a}D_{\text{ee}}{\vec{S}}_{b}+{\vec{S}}_{\epsilon }A_{\mathrm{\epsilon i}}{\vec{I}}_{i}+\ldots \\ +\omega_{n}\left(I_{1}^{z}+I_{2}^{z}\right)+{\vec{I}}_{1}d_{\text{nn}}{\vec{I}}_{2} \end{array}$$ (1)

with the Einstein sum convention of double occurring indices. For the hyperfine coupling A, electron dipolar coupling $D_{\text{ee}}$, and nuclear dipolar coupling $d_{\text{nn}}$, the following general form with a quantization along the *z* axis is used

$$A_{\epsilon i}=\left(\begin{array}{ccc} A_{\epsilon i}^{++} & A_{\epsilon i}^{+-} & A_{\epsilon i}^{+z}\\ A_{\epsilon i}^{-+} & A_{\epsilon i}^{--} & A_{\epsilon i}^{-z}\\ A_{\epsilon i}^{z+} & A_{\epsilon i}^{z-} & A_{\epsilon i}^{\mathrm{zz}} \end{array}\right)$$ (2A)

$$D_{\text{ee}}=\left(\begin{array}{ccc} D^{++} & D^{+-} & D^{+z}\\ D^{-+} & D^{--} & D^{-z}\\ D^{z+} & D^{z-} & D^{\mathrm{zz}} \end{array}\right)$$ (2B)

$$d_{\text{nn}}=\left(\begin{array}{ccc} d^{++} & d^{+-} & d^{+z}\\ d^{-+} & d^{--} & d^{-z}\\ d^{z+} & d^{z-} & d^{\mathrm{zz}} \end{array}\right)$$ (2C)

Spin interactions are often written in the *xyz* rather than in the +−z basis as used in our notation. For the translation between the two, we find

$$A^{z+}=A^{+z}={\left(A^{z-}\right)}^{*}={\left(A^{-z}\right)}^{*}=\frac{A^{\mathrm{xz}}+{\mathrm{iA}}^{\mathrm{yz}}}{2}$$ (3A)

$$A^{++}={\left(A^{--}\right)}^{*}=\frac{A^{\mathrm{xx}}-A^{\mathrm{yy}}+2{\mathrm{iA}}_{\mathrm{xy}}}{4}$$ (3B)

$$A^{+-}=A^{-+}=\frac{A^{\mathrm{xx}}+A^{\mathrm{yy}}}{4}$$ (3C)

$$A^{\mathrm{zz}}=A^{\mathrm{zz}}$$ (3D)

The hyperfine coupling consists in general of a dipolar part and an isotropic Fermi contact part with the latter taking a diagonal form in the *xyz* basis ($\bar{a}I_{\mathrm{xyz}}$). The Fermi contact term is important for s-like electron orbitals with short electron-nuclear distances or delocalized electrons, e.g., P dopants in silicon or quantum dots. The Fermi contact term can exceed several hundred MHz while the dipolar hyperfine coupling for an ^1^H nuclear spin 3 or 5 Å away from the (point charge) electron is around 3 or 0.6 MHz, respectively. Since only the $A^{\mathrm{zz}}$, $A^{+-}$, and $A^{-+}$ terms from Eq. 3 A to D depend on the Fermi contact hyperfine coupling, only these might exceed a few megahetz in cases with the dipolar hyperfine coupling of a few megahertz or less. If the wave functions of the two electrons overlap, then this results in an isotropic exchange interaction $J_{\text{ee}}$, similar to the Fermi contact part. Hence, an eventual $J_{\text{ee}}$ can be absorbed into $D_{\text{ee}}$.

Throughout this work, we assume a positive gyromagnetic ratio γ (<46 MHz/T) for nuclear spins, resulting in opposite preferred spin directions for electrons ($\gamma_{e}\approx -28$ GHz/T) and nuclei. We define $\omega_{n}=|\omega_{n,L}|=|-\gamma_{n}B_{0}|$ and $\omega_{e}=|\omega_{e,L}|=|-\gamma_{e}B_{0}|$ such that both are positive frequencies. The different sign of the gyromagnetic ratios of electrons and nuclei leads to opposite magnetic quantum numbers defining the ground state, i.e., for electrons the spin-down state ($m_{S}=-1/2$), denoted by ↓, has lower energy than the spin-up state ($m_{S}=+1/2$), denoted by ↑. For nuclei, this is inverted with $m_{I}=+1/2$ (spin-up, denoted by ⇑) lower in energy than $m_{I}=-1/2$ (spin-down, denoted by ⇓). This notation with ↑, ↓, ⇑, and ⇓ is more common in physics compared to the α ($m_{I}=+1/2$) and β ($m_{I}=-1/2$) notation in magnetic resonance (NMR and EPR) and offers in the current case the advantage that electron and nuclear spins are easy to distinguish.

**Fig. 1.**
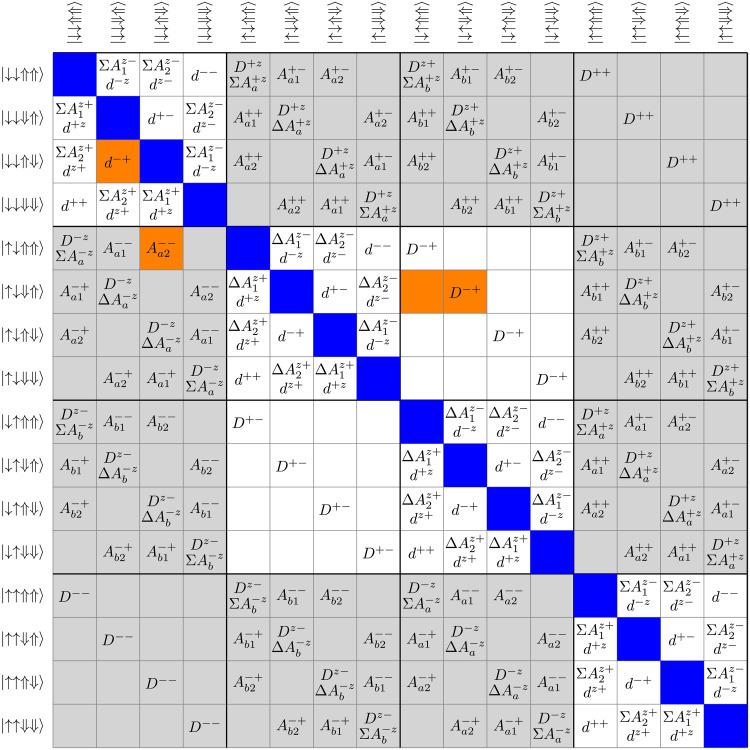
Two-electron two-nucleus spin system involving electron and nuclear dipolar as well as hyperfine couplings. The diagonal matrix elements (colored in blue) form $H_{0}$ in the following and are omitted for clarity with an example given by Eq. 1. Off-diagonal elements (without sign) compose V. V consists of an inner part with quadratic blocks along the diagonal (colored in white), which conserve the total electron quantum number, while the outer part involves net electron flips (shaded in gray). More details are given in the section, “Two-electron two-nucleus spin system.” For elements highlighted in orange, the effective Hamiltonian elements are discussed in detail in the sections, “Electron-mediated spin diffusion (EMSD),” “Electron-nuclear flip-flip or flip-flop,” “Triple spin flips,” and “Electron-nuclear four-spin flip-flops.”

The 16-by-16 matrix of the four-spin model of Eq. 1 is sketched in Fig. 1 and can be rewritten as $H=H_{0}+V=H_{0}+V_{\text{inner}}+V_{\text{outer}}$. $H_{0}$ is the diagonal part of the Hamiltonian containing the electron and nuclear Zeeman energy and the *zz* elements of the hyperfine and dipolar couplings with an example for the diagonal energy levels given by

$$\begin{array}{c} E_{3}=E_{↓↓⇑⇓}=-\frac{1}{2}\left(\omega_{e}+\Delta \omega_{e,a}\right)-\frac{1}{2}\left(\omega_{e}+\Delta \omega_{e,b}\right)+D^{\mathrm{zz}}\\ -\left(\frac{1}{2}\omega_{n}+A_{a1}^{\mathrm{zz}}+A_{b1}^{\mathrm{zz}}\right)+\left(\frac{1}{2}\omega_{n}+A_{a2}^{\mathrm{zz}}+A_{b2}^{\mathrm{zz}}\right)-d^{\mathrm{zz}} \end{array}$$ (4)

The 16 energy levels can be grouped into four spin-up and spin-down parallel electron states each and eight (two times four) antiparallel electron states as indicated by white shadings in Fig. 1. These quadratic blocks around the diagonal form $V_{\text{inner}}$ and are characterized by conservation of the total electron quantum number ($m_{S}=m_{S_{a}}+m_{S_{b}}$). The ten remaining 4-by-4 blocks form $V_{\text{outer}}$ and result in a change of the total electron quantum number $m_{S}$ (net electron flip). Within the diagonal 4-by-4 blocks, the nuclear dipolar and hyperfine couplings can cause transitions between the nuclear spin states. All matrix elements outside the diagonal 4-by-4 blocks involve electron flips or flip-flops either through hyperfine or electron dipolar couplings.

For intermediate (tens to hundreds of mT depending on the other contributions to H) to high magnetic fields, the electron Zeeman energy is much larger than all spin-spin interactions. In such a case, the parallel and antiparallel electron states are energetically well separated. Regarding the other contributions to H: The electron frequency offsets can be hundreds of megahertz at higher fields for defects/radicals with large *g*-factor anisotropy. Nuclear Larmor frequencies $\omega_{n}$ can vary between a few megahertz for low-γ nuclei and intermediate fields of up to several hundred megahertz at higher fields for high-γ nuclei. Hyperfine couplings can span from a few kilohertz for rather distant nuclei to hundreds of megahertz for electrons localized on a specific atom, although in this case most of the coupling would arise from the isotropic Fermi contact part that only affects $A^{+-}$, $A^{-+}$, and $A^{\mathrm{zz}}$ (see above and Eq. 3). Electron dipolar couplings can range into several megahertz for close-by spin-1/2 electrons (no zero-field splitting). Electron exchange couplings $J_{\text{ee}}$, eventually absorbed into $D_{\text{ee}}$, can range much higher, and the same arguments as for the Fermi contact part of the hyperfine coupling would apply. Nuclear dipolar couplings range from hundreds of hertz for low-γ, low-abundance nuclei to several kilohertz for high-γ, high-abundance nuclei such as ^1^H.

To simplify the notation in the following, we define

$$\Sigma A_{i}^{z,+/-/z}=A_{\mathrm{ai}}^{z,+/-/z}+A_{\mathrm{bi}}^{z,+/-/z}$$ (5A)

$$\Delta A_{i}^{z,+/-/z}=A_{\mathrm{ai}}^{z,+/-/z}-A_{\mathrm{bi}}^{z,+/-/z}$$ (5B)

$$\Sigma A_{\epsilon }^{+/-/z,z}=A_{\epsilon 1}^{+/-/z,z}+A_{\epsilon 2}^{+/-/z,z}$$ (5C)

$$\Delta A_{\epsilon }^{+/-/z,z}=A_{\epsilon 1}^{+/-/z,z}-A_{\epsilon 2}^{+/-/z,z}$$ (5D)

$$\Delta \omega_{e}=\Delta \omega_{e,a}-\Delta \omega_{e,b}$$ (5E)

The commas in the superscript of Eq. 5 A to E are only written here for clarity and will be omitted in the following, i.e., $A_{\epsilon i}^{z,z}=A_{\epsilon i}^{\mathrm{zz}}$.

Nuclear spin transitions within the diagonal 4-by-4 blocks are suppressed by the separation between the energy levels, e.g.

$$E_{2}-E_{3}=\Sigma A_{1}^{\mathrm{zz}}-\Sigma A_{2}^{\mathrm{zz}}=\Delta A_{a}^{\mathrm{zz}}+\Delta A_{b}^{\mathrm{zz}}$$ (6A)

$$E_{4}-E_{3}=\omega_{n}+\frac{\Sigma A_{1}^{\mathrm{zz}}}{2}+\frac{d^{\mathrm{zz}}}{2}$$ (6B)

unless the $\omega_{n}$ matches the hyperfine couplings, or the hyperfine couplings would be symmetric, causing an energy level degeneracy. Outside of the diagonal 4-by-4 blocks, all elements of $V_{\text{outer}}$ are much smaller than $\omega_{e}$. For a large enough electron energy offset $\Delta \omega_{e}=\Delta \omega_{e,a}-\Delta \omega_{e,b}$, electronic flip-flops by $D^{+-}$ and $D^{-+}$ are suppressed unless an energy-level degeneracy would occur for a special combination of electron energy offsets and hyperfine couplings. Therefore, for large enough magnetic fields, hyperfine couplings, and electron energy offsets, the spin dynamics in the four-spin system is suppressed to first order.

The effective Hamiltonian is calculated by $H^{\text{eff}}=e^{S}He^{-S}$, where S is given in lowest order by $V+\left[S,H_{0}\right]=0$ (off-diagonal V and diagonal $H_{0}$) such that $H^{\text{eff}}=H_{0}+1/2\left[S,V\right]+O\left(V^{3}\right)$. Thus, the calculation of the effective Hamiltonian transition-matrix elements is mostly multiplication of 4-by-4 matrices and solving a system of (coupled) linear equations. The major advantages of the chosen approach is its ability to derive several relevant spin-transfer processes in one step, applicability to spin system of more than two or three spins and straightforward implementation in computer algebra systems.

Here, we apply two separate Schrieffer-Wolff transformations to $V_{\text{inner}}$ and $V_{\text{outer}}$ although this is identical to applying it to V with the current structure as discussed in section S1. Applying the Schrieffer-Wolff transformation separately to $V_{\text{inner}}$ and $V_{\text{outer}}$ ensures that the off-diagonal perturbation is smaller than the energy gap of the diagonal as the $A_{\epsilon i}^{+-}$ elements can exceed $\omega_{n}$ but not $\omega_{e}$. However, $\Delta \omega_{e}$ might not always be larger than $D^{+-}$ causing a breakdown of the Schrieffer-Wolff-transformation. Assuming that the Schrieffer-Wolff transformation can be applied, an example for the renormalized energies in the effective Hamiltonian is given by

$$\begin{array}{c} E_{3}^{\text{eff}}=E_{3}-\frac{1}{2}\left[\frac{4d^{-+}d^{+-}}{\Delta A_{a}^{\mathrm{zz}}+\Delta A_{b}^{\mathrm{zz}}}+\frac{\left(\Sigma A_{1}^{z-}+d^{-z}\right)\left(\Sigma A_{1}^{z+}+d^{+z}\right)}{\Sigma A_{1}^{\mathrm{zz}}+d^{\mathrm{zz}}+2\omega_{n}}\right.\\ \left.-\frac{\left(\Sigma A_{2}^{z-}-d^{z-}\right)\left(\Sigma A_{2}^{z+}-d^{z+}\right)}{\Sigma A_{2}^{\mathrm{zz}}-d^{\mathrm{zz}}+2\omega_{n}}\right]+O\left(\omega_{e}^{-1}\right) \end{array}$$ (7)

The other energies can be calculated with a Mathematica notebook as shown in section S7.

In the following, we will discuss several matrix elements of $H^{\text{eff}}$. Specifically, we will look into different processes ranging from no electron flips (just their passive presence) over single electron flips (single-quantum transition) to electron flip-flops (ZQ transition).

### Electron-mediated spin diffusion (EMSD)

This process describes a nuclear flip-flop in the passive presence of the electrons, e.g., ∣↓↓⇓⇑〉→∣↓↓⇑⇓〉 connecting two states that are separated by an energy on the order of the nuclear Larmor frequency

$$\begin{array}{cc} H_{3,2}^{\text{eff}} & =\frac{1}{4}\sum_{i}\frac{\left(\Sigma A_{1}^{z+}-d^{+z}\right)\left(\Sigma A_{2}^{z-}-d^{z-}\right)}{\Sigma A_{i}^{\mathrm{zz}}-d^{\mathrm{zz}}+2\omega_{n}}\\ & -\frac{1}{4}\sum_{i}\frac{\left(\Sigma A_{1}^{z+}+d^{+z}\right)\left(\Sigma A_{2}^{z-}+d^{z-}\right)}{\Sigma A_{i}^{\mathrm{zz}}+d^{\mathrm{zz}}+2\omega_{n}}\\ & +\sum_{\epsilon \neq \kappa }\sum_{i\neq j}\left[\frac{2A_{\epsilon 1}^{++}A_{\epsilon 2}^{--}}{A_{\kappa i}^{\mathrm{zz}}-A_{\epsilon j}^{\mathrm{zz}}-d^{\mathrm{zz}}+D^{\mathrm{zz}}-2\omega_{e,\epsilon }+2\omega_{n}}\right.\\ & +\left.\frac{2A_{\epsilon 1}^{-+}A_{\epsilon 2}^{+-}}{A_{\epsilon i}^{\mathrm{zz}}-A_{\kappa j}^{\mathrm{zz}}-d^{\mathrm{zz}}+D^{\mathrm{zz}}-2\omega_{e,\epsilon }-2\omega_{n}}\right]\\ & \approx -\sum_{\epsilon }\frac{A_{\epsilon 1}^{++}A_{\epsilon 2}^{--}+A_{\epsilon 1}^{-+}A_{\epsilon 2}^{+-}}{\omega_{e,\epsilon }} \end{array}$$ (8)

The first two terms are mostly negligible as these cancel out for $d\ll \omega_{n},\Sigma A_{i}^{+/-/z}$. The latter two terms describe a pseudo dipolar coupling (*50*), often called electron-mediated spin diffusion (EMSD), discussed as a limiting process in quantum dots (*24*, *25*) and quantum computing (*51*). Considering the tensorial nature of the hyperfine coupling, only parts of the hyperfine coupling contribute to the nuclear-nuclear spin flip-flops instead of the full coupling. Furthermore, two different pathways exist, either through $A_{b1}^{++}A_{b2}^{--}$ or $A_{b1}^{-+}A_{b2}^{+-}$. Because EMSD scales as hyperfine coupling squared divided by electron Larmor frequency, its frequency is in the range of hertz. Thus, it is a low frequency, broad nonresonant matrix element because the denominator is dominated by $\omega_{e}$. In contrast, all the following discussed matrix elements are (partially) resonant and only become relevant over a rather narrow frequency interval.

In DNP, this term might transport polarization between hypershifted nuclei under all conditions. The magnitude of the rate constant will depend in a perturbation treatment (*52*) on the square of the coupling term in the Hamiltonian and the intensity of the ZQ line at frequency zero.

### Electron-nuclear flip-flip or flip-flop

This process describes a joint one-nucleus-one-electron flip-flip [double-quantum (DQ)] or flip-flop (ZQ) process, e.g., ∣↓↓⇑⇓〉→∣↑↓⇑⇑〉 with energies separated on the order of the electron Larmor frequency

$$\begin{array}{cc} H_{5,3}^{\text{eff}} & =\frac{1}{2}\left[\frac{\left(\Delta A_{a}^{+z}-D^{+z}\right)\left(\Delta A_{2}^{z+}+d^{z+}\right)}{\Delta A_{2}^{\mathrm{zz}}+d^{\mathrm{zz}}-2\omega_{n}}\right.\\ & -\frac{\left(\Sigma A_{a}^{+z}-D^{+z}\right)\left(\Sigma A_{2}^{z+}-d^{z+}\right)}{\Sigma A_{2}^{\mathrm{zz}}-d^{\mathrm{zz}}+2\omega_{n}}+\frac{2A_{a1}^{+-}d^{++}}{\Sigma A_{a}^{\mathrm{zz}}-\Sigma A_{b}^{\mathrm{zz}}-4\omega_{n}}\\ & +\frac{2A_{b2}^{++}D^{+-}}{\Sigma A_{a}^{\mathrm{zz}}-\Sigma A_{b}^{\mathrm{zz}}+\Delta \omega_{e}}-\left.\frac{2A_{a1}^{++}d^{-+}}{\Delta A_{a}^{\mathrm{zz}}+\Delta A_{b}^{\mathrm{zz}}}\right]+O\left(\omega_{e}^{-1}\right) \end{array}$$ (9)

The probability of such a transition driven by the Hamiltonian of Eq. 9 is negligible, but under MW irradiation, it becomes important for solid-effect (SE) DNP. In quantum information processing, this matrix element describes an electron-nucleus two qubit gate that can be used to initialize the nuclear qubits (*35*).

Because the electron nuclear flip-flop only requires a two spin system (one electron and one nucleus), we can simplify Eq. 9 to

$$\begin{array}{c} H_{2,3}^{\text{eff},1e1n}=-\frac{A^{+z}A^{z+}}{4}\left(\frac{1}{A^{\mathrm{zz}}-2\omega_{n}}+\frac{1}{A^{\mathrm{zz}}+2\omega_{n}}\right)+O\left(\omega_{e}^{-1}\right)\\ =-\frac{{\left(A^{z+}\right)}^{2}}{2}\frac{A^{\mathrm{zz}}}{{\left(A^{\mathrm{zz}}\right)}^{2}-{\left(2\omega_{n}\right)}^{2}}+O\left(\omega_{e}^{-1}\right) \end{array}$$ (10)

where we used $A^{z+}=A^{+z}$ from Eq. 3A. This polarization transfer might be responsible for the observed near-unity polarization in optically pumped quantum dots (*53*).

If MW irradiation is applied to the electron-nuclear spin system in DNP, then this transition would be called the SE. MW irradiation is tuned to $\omega_{e}-\omega_{n}$ (ZQ) or $\omega_{e}+\omega_{n}$ (DQ) to create a nuclear hyperpolarization (*11*, *13*). In section S2, SE and resonant mixing (RM) are derived in a one electron–one nucleus spin system with MW irradiation, underlining the ability of the used Schrieffer-Wolff approach to describe the known and unknown processes in electron-nuclear spin systems.

### Triple spin flips

This process describes a joint electronic flip-flop and a nuclear flip, e.g., ∣↓↑⇑⇑〉→∣↑↓⇓⇑〉 of two states that are separated by energies on the order of the nuclear Larmor frequency

$$\begin{array}{c} H_{6,9}^{\text{eff}}=-\frac{D^{+-}}{2}\left[\frac{\Delta A_{1}^{z-}+d^{-z}}{\Sigma A_{a}^{\mathrm{zz}}-\Sigma A_{b}^{\mathrm{zz}}+2\Delta \omega_{e}}\right.-\frac{\Delta A_{1}^{z-}-d^{-z}}{\Delta A_{1}^{\mathrm{zz}}-\Delta A_{2}^{\mathrm{zz}}-2\Delta \omega_{e}}\\ +\frac{\Delta A_{1}^{z-}+d^{-z}}{\Delta A_{1}^{\mathrm{zz}}+d^{\mathrm{zz}}-2\omega_{n}}-\left.\frac{\Delta A_{1}^{z-}-d^{-z}}{\Delta A_{1}^{\mathrm{zz}}-d^{\mathrm{zz}}+2\omega_{n}}\right]+O\left(\omega_{e}^{-1}\right) \end{array}$$ (11)

Triple spin flips with an electronic flip-flop and the flip of a hyperfine coupled nucleus (flip-flop-flip transition) are the basis for cross effect (CE) and thermal mixing (TM) DNP (*54*–*56*). For the CE, DNP is efficient if $\Delta \omega_{e}≃\pm \omega_{n}$, creating an energy level degeneracy with the energy difference of the electrons available to flip a nuclear spin (*11*–*13*, *29*). Ignoring the hyperfine couplings in the denominator and all nuclear dipolar couplings gives in good approximation

$$H_{6,10}^{\text{eff}}\approx -\frac{D^{+-}\Delta A_{1}^{z-}}{2}\left[\frac{\omega_{n}-\Delta \omega_{e}}{\Delta \omega_{e}\omega_{n}}\right]\overset{\Delta \omega_{e}=-\omega_{n}}{=}-\frac{D^{+-}\Delta A_{1}^{z-}}{\omega_{n}}$$ (12)

for the polarization transfer by triple spin flips if the matching condition is fulfilled, reproducing the triple spin flip result from (*54*). To generate hyperpolarization, MW irradiation is required to generate a population imbalance between the two electrons involved in the CE process (*11*, *12*, *29*). Such a Hamiltonian will drive not only heteronuclear polarization transfer but also homonuclear ZQ polarization transfer on the electrons, which is mechanistically very similar to the MIRROR (*57*, *58*) experiment.

The electronic dipolar flip-flop represents an energy available for transfer to other coupled spins. In the following, we will discuss that this energy could be used to induce nuclear flip-flops of hyperfine coupled nuclei, which would be suppressed by hyperfine coupling differences exceeding the nuclear dipolar coupling.

### Electron-nuclear four-spin flip-flops

This process describes a joint electron flip-flop and nuclear flip-flop, e.g., ∣↓↑⇑⇓〉→∣↑↓⇓⇑〉 where the energy difference of the two states is on the order of the difference frequency of the two electrons

$$\begin{array}{cc} H_{6,10}^{\text{eff}} & =-D^{+-}d^{-+}\left[\frac{1}{\Delta A_{1}^{\mathrm{zz}}-\Delta A_{2}^{\mathrm{zz}}+\Delta \omega_{e}}\right.\\ & +\left.\frac{1}{\Delta A_{1}^{\mathrm{zz}}-\Delta A_{2}^{\mathrm{zz}}-\Delta \omega_{e}}+\frac{2}{\Delta A_{1}^{\mathrm{zz}}-\Delta A_{2}^{\mathrm{zz}}}\right]+O\left(\omega_{e}^{-1}\right) \end{array}$$ (13)

These terms have similarities to the CE transition discussed above as they combine an electron flip-flop with a nuclear transition. However, in this case, the nuclear transition is a flip-flop mediated by the dipolar interaction (in total: flip-flop-flip-flop). These three terms scale as $D^{+-}d^{-+}\approx O$(MHz ⋅ kHz) and become resonant if the nuclei have either identical hyperfine couplings $\Delta A_{a}^{\mathrm{zz}}-\Delta A_{b}^{\mathrm{zz}}=\Delta A_{1}^{\mathrm{zz}}-\Delta A_{2}^{\mathrm{zz}}≃0$ or if the difference matches the electron frequency difference $\Delta A_{1}^{\mathrm{zz}}-\Delta A_{2}^{\mathrm{zz}}≃\pm \Delta \omega_{e}$. The former case suggests that spin diffusion between spins with identical hyperfine coupling can be faster than bulk spin diffusion. The latter case describes electron-nuclear four-spin flip-flops as an energy conserving process, independent of the interaction with MW photons or phonons causing nuclear spin and spectral diffusion close to the electron.

Similar to triple spin flips, this electron-nuclear four-spin flip-flop can drive homonuclear ZQ polarization transfer on the electrons but in addition also on the nuclei and electron-nuclear zero- and double-quantum polarization transfer. However, the magnitude will be much smaller since the magnitude is determined by the product of the electron and the nuclear dipolar coupling while the CE Hamiltonian contains the product of the electron dipolar coupling with the hyperfine coupling to the nuclei.

For a thermal electron polarization close to unity at liquid helium temperatures and few Tesla magnetic fields, electron-nuclear four-spin flip-flops are suppressed as few electron pairs with opposite polarization are available. However, this changes upon MW irradiation reducing the electron polarization (cf. Eq. 16) similar to the probability for triple spin flips to occur.

Electron-nuclear four-spin flip-flops can be understood as a heteronuclear CE with the two nuclei having different resonance frequencies (in this case due to different hyperfine couplings). A heteronuclear CE has been investiagated in (*59*). A heteronuclear CE would explain the equilibration of polarization in samples containing more than one NMR-active nuclei (at least locally close to the electron) that has been usually described through a spin bath approach (*60*, *61*) adapted from a classical description of TM.

If dopant clusters (with multiple electrons) are used for quantum information processing (*62*), then this term might limit the coherence and lifetime of nuclear spin qubits.

### The effective Hamiltonian

In the above equations, we did not assume any particular symmetry of the spin interactions besides the existence of an intermediate to strong magnetic field, creating a quantization axis. Furthermore, we did not include any type of MW irradiation unless explicitly stated. Thus, all these processes occur in thermalized systems as often encountered in quantum information processing. In DNP, for large enough electron frequency differences $\Delta \omega_{e}$, MW irradiation at one of the electron frequencies causes (damped) Rabi oscillations, while the other electron remains unaffected. Thus, MW irradiation creates a polarization difference between the two electrons available for transfer to nuclear spins either as (CE) triple spin flips or electron-nuclear four-spin flip-flops. The dependence of the triple spin flip rate and nuclear spin flip-flops close to the electron with MW power (electron saturation) is indirectly investigated in the next section.

The above effective two-electron two-nucleus model is limited to processes involving two interactions at most as only the lowest order of the Schrieffer-Wolff transformation was used. Higher-order Schrieffer-Wolff transformations (*47*) could resolve this. Terms of the form DAA might show up, e.g., for four-spin CE (*59*). In the presented model, the transition matrix elements for these transitions are nonzero, but a correct description is not possible as three interaction processes cannot be described with a lowest-order Schrieffer-Wolff transformation. Extension to higher-order transformations in the laboratory frame might result in very long expressions. Thus, effective Hamiltonians in the rotating frame can be used to simplify the result while retaining terms not scaling with $O\left(\omega_{e}^{-n}\right)$, n≥1.

We tested the Schrieffer-Wolff transformation of the two-electron two-nucleus system in the electron rotating frame. For the EMSD (Eq. 8), triple spin flips (Eq. 11) and electron-nuclear four-spin flip-flops (Eq. 13), we found the same expressions as in the laboratory frame except from the truncated $O\left(\omega_{e}^{-1}\right)$ terms. For the single-electron flip processes, e.g., electron-nuclear flip-flip or flip-flops, the rotating frame transformation adds additional terms that were scaling in the lab frame with $\omega_{e,\epsilon }^{-1}$ and in the rotating frame will scale with $\Delta \omega_{e,\epsilon }^{-1}$. Other terms scaling as $O\left(\omega_{e}^{-1}\right)$ in the laboratory frame are truncated.

Future studies might combine the effective Hamiltonian with relaxation or describe the coupling with phonons or (cavity) photons in a quantized approach, i.e., through creation and annihilation operators. This might lead to the discovery of so-far undiscovered quantum many-body effects causing hyperpolarization.

Next, we will discuss the effective Hamiltonian elements from the perspective of DNP experiments, which are typically conducted at cryogenic temperatures, a few Tesla magnetic fields and mostly with organic radicals. EMSD has been studied in quantum dots with extended electron wave functions (Fermi contact hyperfine coupling), connecting a large number of nuclear spins via EMSD. In DNP, the electron wave functions extend only over a few nuclear spins. Thus, only those few spins might experience effective couplings via EMSD [$O\left(\omega_{e}^{-1}\right)$ scaling] at a few Tesla magnetic fields on the order of several (tens of) hertz, which makes the observation of EMSD in DNP experiments difficult to impossible. Electron-nuclear flip-flops and triple spin flips are the prototypical microscopic DNP mechanisms and have been studied extensively as discussed above.

Electron-nuclear four-spin flip-flops have not been studied experimentally, and it is challenging to measure these directly. Thus, we turn to indirect evidence for the relevance of this process in DNP polarization transfer. For this, we explore the similarity to triple spin flip DNP with both relying on an electronic flip-flop process providing the energy for a nuclear excitation, which requires opposite spin direction of the involved electrons, e.g., ∣↑↓〉 or ∣↓↑〉. Thus, for electron polarization close to unity as encountered in a few Tesla magnetic field and liquid-helium temperatures, i.e., under dissolution DNP conditions, electronic flip-flops would be rare and the electron polarization needs to be considered.

Section 8.1.4 of the book by Wenckebach (*13*) derives a rate equation for the nuclear polarization $P_{n}$ in an electron-electron-nucleus three-spin system based on triple spin flips considering the electron polarization (given by the difference of the density matrix components $\rho_{+-+}$ and $\rho_{-+-}$) as

$$\frac{\partial P_{n}}{\partial t}=\frac{\partial }{\partial t}\left(\rho_{+-+}-\rho_{-+-}\right)=-\frac{1}{2}W_{\text{een}}\left(1-P_{e,a}P_{e,b}\right)\left[P_{n}-\frac{P_{e,a}-P_{e,b}}{1-P_{e,a}P_{e,b}}\right]$$ (14)

where $P_{e,\epsilon }$ is the polarization of the two involved electrons and $W_{\text{een}}$ the triple spin transition rate.

Adding a second nuclear spin to Eq. 14 yields

$$\begin{array}{cc} \frac{\partial P_{n,i}}{\partial t} & =\frac{\partial }{\partial t}\left(\rho_{+-+-}-\rho_{-+-+}\right)\\ & =-\frac{1}{4}W_{\text{eenn}}\left(1-P_{e,a}P_{e,b}\right)\left(1-P_{n,i}P_{n,j}\right)\left[\frac{P_{n,i}-P_{n,j}}{1-P_{n,i}P_{n,j}}-\frac{P_{e,a}-P_{e,b}}{1-P_{e,a}P_{e,b}}\right] \end{array}$$ (15)

with $W_{\text{eenn}}$ the electron-nuclear four-spin flip-flop transition rate, similar to the triple spin flip transition rate in Eq. 14 but with a nuclear dipolar flip-flop instead of a nuclear flip and evaluated for electron-nuclear four-spin flip-flops ($\Delta \omega_{e}\pm \left(\Delta A_{1}^{\mathrm{zz}}-\Delta A_{2}^{\mathrm{zz}}\right)$, cf. Eq. 13).

Because both Eqs. 14 and 15 have similar dependence on the electron polarization (and electron spectral line shape as discussed in section S3), it can be expected that electron systems prone to triple spin flips feature electron-nuclear four-spin flip-flops with the latter possibly present in narrow-line radicals, i.e., the electron line is narrower than $\omega_{n}$ as for example in trityl, if the hyperfine coupling differences are smaller than the electron line width. Section S3 provides a more detailed discussion of the possibility of electron-nuclear four-spin flip-flops based on the electron line shape. The similar dependence of triple spin flips and electron-nuclear four-spin flip-flops on the electron polarization suggests a similar dependence on MW irradiation, i.e., MW irradiation reduces the electron polarization at the irradiation frequency by inducing damped Rabi oscillations as discussed in the next section. Hence, a similar dependence of the hyperpolarization creation (macroscopic quantity associated with triple spin flips) and transport of hyperpolarization from hypershifted to bulk spins (macroscopic quantity associated with electron-nuclear four-spin flip-flops) would represent evidence for the relevance of electron-nuclear four-spin flip-flops. In the remainder of this manuscript, we will simulate the MW power-dependent HypRes-on data from Chessari *et al.* (*9*) with a macroscopic two-compartment model to provide experimental relevance for electron-nuclear four-spin flip-flops.

Before this, we would like to remark that electron spectral diffusion (eSD) might represent an additional complication in the above rate equation description. eSD might distribute the reduced electron polarization in frequency space, which reduces the polarization differences between electron spin pairs. However, eSD remains challenging to understand and model (*13*, *63*–*65*), and hence, we left this out of the above discussion.

### Electron saturation dependence of the spin transport between hypershifted and bulk nuclei

**Fig. 2.**
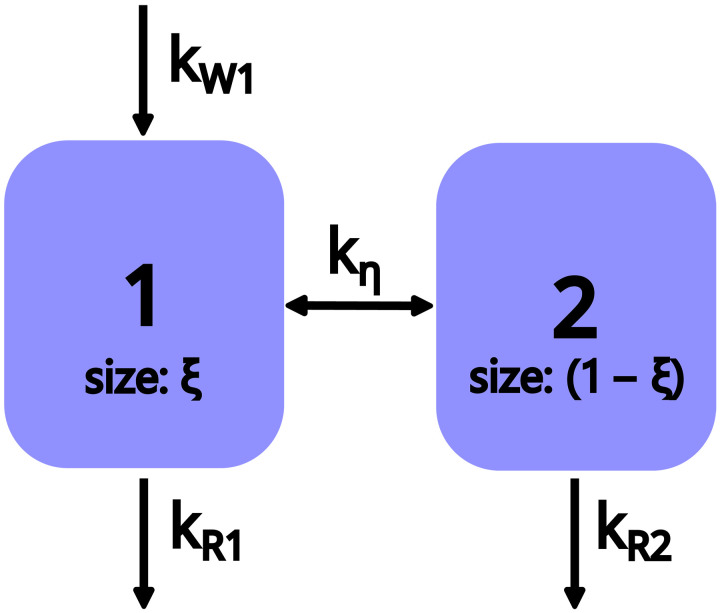
Coupled two-compartment model of hyperpolarization. The injection of polarization into the first compartment is given by $k_{W1}$. The coupling to an eventual second compartment is given by $k_{\eta }$. The polarization in the two compartments decays with $k_{R1}$ and $k_{R2}$. ξ describes the relative size of the first compartment and (1−ξ) that of the second.

In the following, we will apply a two-compartment model of hyperpolarization as sketched in Fig. 2 and discussed in detail in section S4 to the HypRes-on data from (*9*). This approach enables us to quantify the increase in coupling between the hypershifted and bulk spins by MW irradiation, which is considered to describe the spin diffusion close to the electron. The sample used in these experiments is TEMPOL in ^1^H glassy matrices in which DNP is commonly attributed to triple spin flips. For triple spin flips, the polarization difference between the two electron spins leads to the nuclear hyperpolarization. If the coupling between the hypershifted and the bulk spin compartments shows the same dependence on the electron saturation by MW irradiation, then this would be a strong indication that electron-nuclear four-spin flip-flops with the dependence on the electron spin polarization difference are the main process for nuclear flip-flops (spin diffusion) close to electrons.

In MW-on HypRes or HypRes-on experiments, the sample was first hyperpolarized before broadband saturation pulses were applied to saturate the bulk nuclear polarization. During the saturation, the MW was switched to the frequency of the other DNP lobe, reversing the sign of the DNP injection, and eventually its power was adjusted (*9*). This creates two competing polarization dynamics: First, the positive polarization from the build-up is still stored in the hypershifted spins close to the electrons and diffuses into the bulk, with a time constant given by the inter-compartment coupling term. Second, the negative DNP process injects hyperpolarization with the opposite sign first into the unobservable (hypershifted) first compartment and then into the bulk spins. We note the similar polarization maximum during the HypRes-on experiment for all MW powers (cf. fig. S3), possibly suggesting a similar scaling of DNP injection and intercompartment coupling (spin diffusion from hypershifted to bulk spins) with MW power.

A system compromised of the RF ”invisible” hypershifted spins and the bulk spins can be described by a two-compartment model. To model this, we extend the previously introduced one-compartment rate equation model (*48*, *49*) to a coupled two-compartment model: Fig. 2 sketches the basic idea of the model with DNP/hyperpolarization injection (creation) only into the first compartment (relative size ξ), a coupling between the two compartments and a separate relaxation rate constant for each compartment. For simplicity and in analogy to the one-compartment model (*48*), we ignore a thermal equilibrium polarization as nuclear polarization enhancements exceeding 100 can be achieved in many materials, rendering the thermal polarization small compared to typical measurement uncertainties. The details of the model can be found in section S3.

**Fig. 3.**
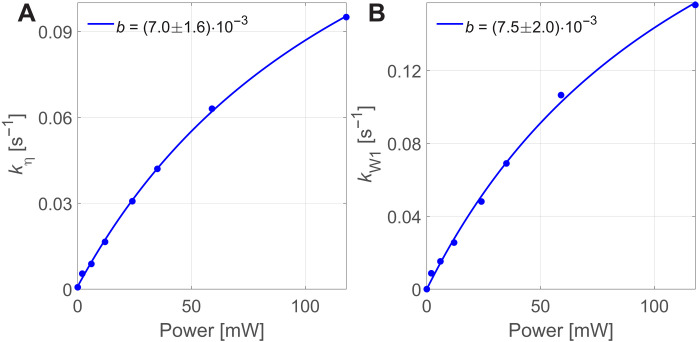
Two-compartment modelling of HypRes-on data. Power dependence of the inter-compartment coupling constant $k_{\eta }$ (**A**) and injection parameter $k_{W1}$ (**B**) describing the experimental data from (*9*). The size of the first compartment was set to 7% and the relaxation rate constants to zero. More details about the simulations can be found in section S5, particularly fig. S3. The coupling constant $k_{\eta }$ and DNP injection $k_{W1}$ are fitted with $a\left(1-\frac{1}{\mathrm{bx}+1}\right)$ (compare Eq. 10) with $\mathrm{bx}=\gamma_{e}^{2}B_{1,\text{MW}}^{2}T_{2,e}T_{1,e}$ being the saturation factor and an additional offset for the coupling constant attributed to the finite thermal coupling (*8*), although the offset is fitted to be effectively zero. The similar scaling of DNP injection and transport from the hidden into the bulk spins suggests a similar origin with the DNP injection originating from triple spin flips.

The parameters of the best fits to the HypRes-on data with the two-compartment model as described in eqs. S18 are shown in Fig. 3. The fitting is insensitive to the relaxation rates of the two compartments owing to rather low polarization levels and short experimental durations and, hence, the relaxation rate constants are set to zero. This leaves the DNP injection into the first compartment $k_{W1}$ and the intercompartment coupling $k_{\eta }$ as the remaining fit parameters. More details on the simulations including the fits to the experimental data can be found in section S5.

We fit the best fit parameters as shown in Fig. 3 with a model describing the saturation of the electrons by the MW irradiation based on

$$1-\frac{P_{e,\infty }}{P_{0,e}}=1-\frac{1}{\gamma_{e}^{2}B_{1,\text{MW}}^{2}T_{2,e}T_{1,e}+1}$$ (16)

with $T_{1,e}$, $T_{2,e}$ being the electronic relaxation times and $B_{1,\text{MW}}$ the MW $B_{1}$ field. Because we do not know the relationship between applied MW power and $B_{1,\text{MW}}$, we use the generalized saturation parameter *b* (cf. caption of Fig. 3). Equation 16 is derived from the Torrey model (*66*) of damped Rabi oscillations, which in this case is equivalent to the *z* part of the time-independent steady-state solutions of the Bloch equations (cf. section S6).

The coupling constant $k_{\eta }$ and DNP injection $k_{W1}$ show a nearly identical saturation parameter in Fig. 3, suggesting a common origin. We note that the coupling and injection parameters are for the highest MW power only around one half of their fitted maximum value, allowing for a much higher DNP injection into the bulk if higher MW powers would be available. However, higher MW power at liquid helium temperatures likely would not result in higher steady-state polarization as the relaxation scales linear with the electron saturation although the build-up time could be shortened (*49*).

## DISCUSSION

The two-electron two-nucleus spin system discussed above describes several nuclear and electron-nuclear spin transfer processes of which some are known in different communities. Two different processes possibly leading to nuclear spin diffusion around electrons can be compared: (i) EMSD is present under any conditions and is non-resonant but strongly suppressed by its $O\left(\omega_{e}^{-1}\right)$ scaling. Therefore, EMSD is irrelevant for most DNP experiments but highly relevant for quantum dots or information processing (*24*, *25*, *51*). (ii) Electron-nuclear four-spin flip-flop processes are energy conserving if the resonance condition is met and do not involve an immediate interaction with the lattice or MW field but require electrons with different spin directions available as provided during MW irradiation (cf. Eq. 16) or (*11*). Thus, under MW irradiation, the polarization transfer would be expected to scale similar to DNP relying on triple spin flips (as for 50 mM TEMPOL in ^1^H glassy matrices) as both rely on the saturation of one electron population by MW irradiation, consistent with our results described in Fig. 3.

Hence, our work suggests that spin diffusion close to electrons for ^1^H-rich electron environments is relatively fast as electron-nuclear four-spin flip-flops enable nuclear flip-flops even for nuclei with different energies (nuclear spectral diffusion) due to hyperfine couplings. For these electron-nuclear four-spin flip-flops, the electron polarization in at least parts of the electron spectrum needs to be clearly below unity, e.g., MW irradiation or not to high thermal electron polarizations, to enable electronic flip-flops. This suggests that a spin diffusion barrier does not exist and all spins can contribute to the transport of nuclear hyperpolarization toward the bulk for large enough electronic and nuclear dipolar couplings, e.g., in ^1^H glassy matrices. This is supported by selective deuteration experiments in ^1^H-rich electron environments (*19*, *67*), relaxation (*4*), Hyp-Res (*8*, *9*), and three-spin solid effect experiments (*6*). Future work might give a quantitative estimate of the spin diffusion close to the electron for a specific material, include relaxation effects, and involve higher order Schrieffer-Wolff transformations (*47*).

## MATERIALS AND METHODS

The Schrieffer-Wolff transformations were computed with Mathematica (section S7). HypRes-on fits were performed with in-house developed MATLAB scripts.

## Supplementary Materials

### This PDF file includes:

Sections S1 to S7 Figs. S1 to S3
