# Supplementary material for: Two-electron two-nucleus effective Hamiltonian and the spin diffusion barrier
Source: Sci Adv. 2025 Jan 3;11(1):eadr7168. doi: 10.1126/sciadv.adr7168 (PMC11698094; doi:10.1126/sciadv.adr7168)
Supplement: Supplementary file 1 — Sections S1 to S7 Figs. S1 to S3 [file sciadv.adr7168_sm.pdf]

Science Advances
AAAS

# Supplementary Materials for

## Two-electron two-nucleus effective Hamiltonian and the spin diffusion barrier

Gevin von Witte *et al.*

Corresponding author: Matthias Ernst, maer@ethz.ch

*Sci. Adv.* **11**, eadr7168 (2025)
DOI: 10.1126/sciadv.adr7168

**This PDF file includes:**

Sections S1 to S7
Figs. S1 to S3

# S1 Additivity of the first-order Schrieffer-Wolff transformation for quadratic block-diagonal and off-block-diagonal interactions

**Definition:** Let $\mathcal{P}_0$ be a projector on the diagonal matrix elements of a given n-dimensional square matrix $\mathbf{V}$ (*70*)

$$\mathcal{P}_0 \bullet \mathbf{V} = \sum_{j=1}^{n} |\psi_j\rangle \langle\psi_j| \mathbf{V} |\psi_j\rangle \langle\psi_j| \qquad . \tag{S1}$$

and

$$\mathcal{Q}_0 \bullet \mathbf{V} = \sum_{\substack{j,k=1 \\ j\neq k}}^{n} |\psi_j\rangle \langle\psi_j| \mathbf{V} |\psi_k\rangle \langle\psi_k| \tag{S2}$$

for the off-diagonal part. Let

$$\mathcal{N}_i = \{(j,k) \mid a_i \leq j,k \leq b_i,\ 1 \leq a_i, b_i \leq n,\ a_i, b_i, j, k \in \mathbb{N}\} \tag{S3}$$

be an interval of real numbers with $\mathcal{N}_i \cap \mathcal{N}_j = \emptyset\ \forall i \neq j$ and $\mathcal{N} = \bigcup_i \mathcal{N}_i$ Then we can define a projector on the block diagonal part of the matrix given by all $\mathcal{N}_i$

$$\mathcal{Q}_{\text{inner}} \bullet \mathbf{V} = \sum_{\substack{(j,k)\in\mathcal{N} \\ j\neq k}} |\psi_j\rangle \langle\psi_j| \mathbf{V} |\psi_k\rangle \langle\psi_k| \tag{S4}$$

and the corresponding projector on the other off-diagonal elements

$$\mathcal{Q}_{\text{outer}} \bullet \mathbf{V} = \sum_{\substack{(j,k)\notin\mathcal{N} \\ j\neq k}} |\psi_j\rangle \langle\psi_j| \mathbf{V} |\psi_k\rangle \langle\psi_k| \tag{S5}$$

**Definition:** The first-order effective Hamiltonian $\mathbf{H}^{\text{eff}}$ from the Schrieffer-Wolff transformation of $\mathbf{H} = \mathbf{H}_0 + \mathbf{V}$ with with a diagonal matrix $\mathbf{H}_0$, i.e. $\mathbf{H}_0 = \mathcal{P}_0 \bullet \mathbf{H}_0$ and an off-diagonal $\mathbf{V}$, i.e. $\mathbf{V} = \mathcal{Q}_0 \bullet \mathbf{V}$, is given by

$$\mathbf{H}^{\text{eff}} = \mathbf{H}_0 + \frac{1}{2}[\mathbf{S}, \mathbf{V}] + \mathcal{O}(\mathbf{V}^3) \tag{S6}$$

with S defined by

$$\mathbf{V} + [\mathbf{S}, \mathbf{H}_0] = 0 \qquad . \tag{S7}$$

**Theorem:** For $\mathbf{V}_{\text{inner}} + \mathbf{V}_{\text{outer}} \coloneqq \mathcal{Q}_{\text{inner}} \bullet \mathbf{V} + \mathcal{Q}_{\text{outer}} \bullet \mathbf{V} = \mathbf{V}$, the first-order Schrieffer-Wolff transformation is given by $\mathbf{H}^{\text{eff}} = \mathbf{H}_0 + \frac{1}{2}[\mathbf{S}, \mathbf{V}] + \mathcal{O}(\mathbf{V}^3) = \mathbf{H}_0 + \frac{1}{2}[\mathbf{S}_{\text{inner}}, \mathbf{V}_{\text{inner}}] + \frac{1}{2}[\mathbf{S}_{\text{outer}}, \mathbf{V}_{\text{outer}}] + \mathcal{O}(\mathbf{V}^3)$ with $\mathbf{S} = \mathbf{S}_{\text{inner}} + \mathbf{S}_{\text{outer}}$.

**Proof:** $\mathbf{S}_{\text{inner}}$ and $\mathbf{S}_{\text{outer}}$ are given by $\mathbf{V}_{\text{inner}} + [\mathbf{S}_{\text{inner}}, \mathbf{H}_0] = 0$ and $\mathbf{V}_{\text{outer}} + [\mathbf{S}_{\text{outer}}, \mathbf{H}_0] = 0$, respectively. Since $\mathcal{P}_0 \bullet \mathbf{H}_0 = \mathbf{H}_0$, $\mathcal{Q}_{\text{inner}} \bullet [\mathbf{S}_{\text{inner}}, \mathbf{H}_0] = [\mathbf{S}_{\text{inner}}, \mathbf{H}_0]$ and $\mathcal{Q}_{\text{outer}} \bullet [\mathbf{S}_{\text{inner}}, \mathbf{H}_0] = 0$ as well as $\mathcal{Q}_{\text{inner}} \bullet [\mathbf{S}_{\text{outer}}, \mathbf{H}_0] = 0$ and $\mathcal{Q}_{\text{outer}} \bullet [\mathbf{S}_{\text{outer}}, \mathbf{H}_0] = [\mathbf{S}_{\text{inner}}, \mathbf{H}_0]$. Then

$$(\mathbf{S}_{\text{inner}}\mathbf{V}_{\text{inner}})_{jk} = \begin{cases} \sum_l (\mathbf{S}_{\text{inner}})_{jl} (\mathbf{V}_{\text{inner}})_{lk} & j, k \in \mathcal{N} \\ 0 & j \vee k \notin \mathcal{N} \end{cases} \tag{S8}$$

and

$$(\mathbf{S}_{\text{outer}}\mathbf{V}_{\text{outer}})_{jk} = \begin{cases} \sum_l (\mathbf{S}_{\text{outer}})_{jl} (\mathbf{V}_{\text{outer}})_{lk} & j, k \notin \mathcal{N} \\ 0 & j \vee k \in \mathcal{N} \end{cases} \tag{S9}$$

which results in $\mathbf{SV} = \mathbf{S}_{\text{inner}}\mathbf{V}_{\text{inner}} + \mathbf{S}_{\text{outer}}\mathbf{V}_{\text{outer}}$. As the same holds for $\mathbf{VS}$, $[\mathbf{S}, \mathbf{V}] = [\mathbf{S}_{\mathbf{inner}}, \mathbf{V}_{\mathbf{inner}}] + [\mathbf{S}_{\mathbf{outer}}, \mathbf{V}_{\mathbf{outer}}]$. □

We speculate that the above theorem could be extended to arbitrary order for the above construction of $\mathbf{V}$ as the Schrieffer-Wolff transformation is given by $\mathbf{H}^{\text{eff}} = e^{\mathbf{S}}\mathbf{H}e^{-\mathbf{S}}$ with a double Schrieffer-Wolff transformation taking the form $\mathbf{H}^{\text{eff}} = e^{\mathbf{S}_2}e^{\mathbf{S}_1}\mathbf{H}e^{-\mathbf{S}_1}e^{-\mathbf{S}_2}$ and for $[\mathbf{S}_1, \mathbf{S}_2] = 0$, $e^{\mathbf{S}_2}e^{\mathbf{S}_1} = e^{\mathbf{S}}$ (Baker-Campbell-Hausdorff formula). A proof for this is beyond the scope of the current manuscript.

# S2 Derivation of solid effect and resonant mixing DNP

We study a one-electron-one-nucleus spin system with MW irradiation to illustrate that the Schrieffer-Wolff transformation applied to this spin system can describe solid effect (SE) and resonant mixing (RM) DNP. Electron and nuclear Zeeman terms as well as the hyperfine coupling are as above. We add MW irradiation with the electron Rabi frequency $\omega_{1s}$ along the $x$-direction of the lab frame coordinate system ($\mathbf{H}_{\mathrm{MW}} = \omega_{1s} S^x \cos(\omega_{\mathrm{MW}} t + \varphi) = \omega_{1s}/2(S^+ + S^-)\cos(\omega_{\mathrm{MW}} t + \varphi)$). To avoid the time dependence of the MW irradiation, we transform into the rotating frame of the MW. To lowest order, this is can understood as a formalized approach to eliminate spin-spin processes that result in net electron flips as these violate energy conservation (*13*) and only the MW irradiation itself can cause net electron flips. In the rotating frame, the one-electron-one-nucleus Hamiltonian takes the form

$$\mathbf{H}^{\mathrm{1e1n,MW}} = \begin{pmatrix} E_1^{\mathrm{1e1n,MW}} & A^{z-}/2 & \omega_{1s}/2 & 0 \\ A^{z+}/2 & E_2^{\mathrm{1e1n,MW}} & 0 & \omega_{1s}/2 \\ \omega_{1s}/2 & 0 & E_3^{\mathrm{1e1n,MW}} & -A^{z-}/2 \\ 0 & \omega_{1s}/2 & -A^{z+}/2 & E_4^{\mathrm{1e1n,MW}} \end{pmatrix} \tag{S10}$$

with $E_1^{\mathrm{1e1n,MW}} = -(\omega_{\mathrm{e}} - \omega_{\mathrm{MW}}) - \omega_{\mathrm{n}} + A^{zz}$ and the other energies following the same approach as for the two-electron two-nucleus system in the lab frame (cf. Eq. (4)). The 'MW' in the superscript indicates the MW rotating frame in the following. Applying the Schrieffer-Wolff transformation as above for this smaller rotating frame system gives

$$H_{2,3}^{\mathrm{eff,1e1n,MW}} = \frac{A^{z+}\omega_{1s}/2}{2} \left[\frac{2\omega_{\mathrm{e}} - 2\omega_{\mathrm{MW}}}{(A^{zz})^2 - (2\omega_{\mathrm{e}} - 2\omega_{\mathrm{MW}})^2} + \frac{2\omega_{\mathrm{n}}}{(A^{zz})^2 - (2\omega_{\mathrm{n}})^2}\right] \tag{S11}$$

which simplifies for $A^{zz} \ll \omega_{\mathrm{n}},\ \omega_{\mathrm{e}} - \omega_{\mathrm{MW}}$ to

$$H_{2,3}^{\mathrm{eff,1e1n,MW}} \approx -\frac{A^{z+}\omega_{1s}/2}{2}\frac{\omega_{\mathrm{e}} - \omega_{\mathrm{MW}} + \omega_{\mathrm{n}}}{\omega_{\mathrm{n}}(\omega_{\mathrm{e}} - \omega_{\mathrm{MW}})} \tag{S12}$$

MW irradiation at $\omega_\mathrm{e} - \omega_\mathrm{MW} = \omega_\mathrm{n}$ in this limit gives

$$H_{\mathrm{SE,DQ}}^{\mathrm{eff,1e1n,MW}} = -\frac{A^{z+}\omega_{1s}/2}{2\omega_\mathrm{n}} \tag{S13}$$

which is the well known scaling of the SE matrix element (*6, 11, 13, 80*).

Assuming $\varepsilon := \omega_\mathrm{e} - \omega_\mathrm{MW} \to 0$, causes $A^{zz} \simeq \omega_\mathrm{e} - \omega_\mathrm{MW} \ll \omega_\mathrm{n}$. For Eq. (S11) this results in

$$H_{RM}^{\mathrm{eff,1e1n,MW}} \approx \frac{A^{z+}\omega_{1s}/2}{2}\left[\frac{2\varepsilon}{(A^{zz})^2 - (2\varepsilon)^2} - \frac{1}{2\omega_\mathrm{n}}\right] \tag{S14}$$

which gives half the transiton matrix amplitude of the solid effect (cf. Eq. (S13)) for $\varepsilon = 0$ and includes a resonance condition for $A^{zz} = \pm 2\varepsilon$. We identify this strong electron-nuclear transition as the recently introduced resonant mixing DNP (*81*). Resonant mixing can lead to a dispersive enhancement of the nuclear polarization around the electron resonance (single quantum EPR transition, $\mathrm{SQ_e}$, cf. Eq. (S14)). For more details on this including a discussion of experimental evidence, the reader is referred to Ref. (*81*).

If higher order Schrieffer-Wolff transformation (*48*) are applied, this might also describe the three-spin solid effect (electron flip with two nuclei flipping) if MW irradiation is included (*6*). At even higher orders, the four-spin solid effect (SE with three nuclei flipping) could be described (*71*).

# S3 Estimation of the electronic contribution to electron-nuclear four-spin flip-flops

To estimate if electron-nuclear four-spin flip-flops can occur, we assume a Gaussian shaped electron line width a broadening $\sigma_\mathrm{e} = 0.15\,\mathrm{GHz}$, i.e.

$$g_\mathrm{e}(\omega_\mathrm{e}) = \frac{1}{\sqrt{2\pi}\sigma_\mathrm{e}} \exp\left\{ -\frac{(\omega_\mathrm{e} - \omega_0)^2}{2\sigma_\mathrm{e}^2} \right\} \tag{S15}$$

centered at $\omega_0 = 197\,\mathrm{GHz}$ (7 T). The line broadening corresponds to a Gaussian full width at half maximum (FWHM) of 0.35 GHz, which resembles the electron line width of TEMPO at 7 T (*50*).

Several further assumptions are necessary to estimate the fraction of electrons that could perform an electron-nuclear four-spin flip-flop for an electron at the MW irradiation frequency $\omega_\mathrm{MW} = 196.9\,\mathrm{GHZ}$. First, a nuclear spin pair with a resonance frequency difference $\Delta\omega_\mathrm{n} = \Delta A_1^{zz} - \Delta A_2^{zz} = 5\,\mathrm{MHz}$ based on hyperfine computations of nitroxide radicals (*19*) is assumed. Thus, an electron with a resonance frequency around $\omega_\mathrm{MW} \pm \Delta\omega_\mathrm{n}$ is required for an electron-nuclear four-spin flip-flop. Assuming that the secondary electrons suitable for electron-nuclear four-spin flip-flops are given by the homogeneous Lorentzian electron line width $\sigma_\mathrm{L} \approx 3\,\mathrm{MHz}$ (FWHM is $2\sigma_\mathrm{L}$) for 40 mM TEMPOL (*67*), the fraction of electrons available for a electron-nuclear four-spin flip-flop is

$$p_\mathrm{e,eenn} = \int_{\omega_\mathrm{MW}+\Delta\omega_\mathrm{n}-\sigma_\mathrm{L}}^{\omega_\mathrm{MW}+\Delta\omega_\mathrm{n}+\sigma_\mathrm{L}} \mathrm{d}\omega_\mathrm{e}\, \frac{1}{\sqrt{2\pi}\sigma_\mathrm{e}} \exp\left\{ -\frac{(\omega_\mathrm{e} - \omega_0)^2}{2\sigma_\mathrm{e}^2} \right\} \approx 1\% \tag{S16}$$

with the Gaussian integral defined by the error functions.

We note that the number of electrons available for an electronic flip-flop is similar for triple spin flips and electron-nuclear four-spin flip-flops. For triple spin flips, the integral needs to be evaluated around $\omega_\mathrm{MW} \pm \omega_\mathrm{n}$ instead of $\omega_\mathrm{MW} \pm \Delta\omega_\mathrm{n}$. Hence, spin systems featuring triple spin flips are able to host electron-nuclear four-spin flip-flops electronically. Electron-nuclear

four-spin flip-flops might even be possible for spin systems with electron lines narrower than the nuclear Larmor frequency, e.g. trityl and $^{1}$H.

A second approach to study the prevalence of electron-nuclear four-spin flip-flops is based on Eq. (15). Expanding the three-spin system from Eq. (15) to many nuclei and electrons yields (*13, 72*)

$$\frac{\partial P_{\mathrm{n}}}{\partial t} = -\frac{1}{\tau_{een}} \left(P_{\mathrm{n}} - P_{0\mathrm{n}}\right) \tag{S17}$$

with the triple spin flip rate

$$\tau_{een} \propto \mathcal{I} = \int_{-\infty}^{+\infty} \mathrm{d}\omega_{\mathrm{e}}\, g_{\mathrm{e}}(\omega_{\mathrm{e}}) g_{\mathrm{e}}(\omega_{\mathrm{e}} - \omega_{\mathrm{n}}) \left[1 - P_{\mathrm{e}}(\omega_{\mathrm{e}}) P_{\mathrm{e}}(\omega_{\mathrm{e}} - \omega_{\mathrm{n}})\right] \tag{S18}$$

and the electron spectral line shape $g_{\mathrm{e}}(\omega_{\mathrm{e}})$. The nuclear steady-state polarization is given by

$$P_{0\mathrm{n}} = \frac{1}{\mathcal{I}} \int_{-\infty}^{+\infty} \mathrm{d}\omega_{\mathrm{e}} g_{\mathrm{e}}(\omega_{\mathrm{e}}) g_{\mathrm{e}}(\omega_{\mathrm{e}} - \omega_{\mathrm{n}}) \left[P_{\mathrm{e}}(\omega_{\mathrm{e}}) - P_{\mathrm{e}}(\omega_{\mathrm{e}} - \omega_{\mathrm{n}})\right] \quad . \tag{S19}$$

Compared to the three-spin system in Eq. (15), the resulting expressions for a macroscopically large spin system are modified by the electron line shape evaluated for two electrons at the triple spin flip condition.

Extending the four-spin system in Eq. (16) to many nuclei and electrons yields for the electron-nuclear four-spin flip-flop rate

$$\begin{aligned} \tau_{\mathrm{eenn}} \propto \int_{-\infty}^{+\infty} \mathrm{d}\omega_{\mathrm{e}} \int_{-\infty}^{+\infty} \mathrm{d}\omega_{\mathrm{n}}\, & g_{\mathrm{e}}(\omega_{\mathrm{e}}) g_{\mathrm{e}}(\omega_{\mathrm{e}} - \Delta\omega_{\mathrm{n}}) \left[1 - P_{\mathrm{e}}(\omega_{\mathrm{e}}) P_{\mathrm{e}}(\omega_{\mathrm{e}} - \Delta\omega_{\mathrm{n}})\right] ... \\ & g_{\mathrm{n}}(\omega_{\mathrm{n}}) g_{\mathrm{n}}(\omega_{\mathrm{n}} - \Delta\omega_{\mathrm{n}}) \left[1 - P_{\mathrm{n}}(\omega_{\mathrm{n}}) P_{\mathrm{n}}(\omega_{\mathrm{n}} - \Delta\omega_{\mathrm{n}})\right] \end{aligned} \tag{S20}$$

with $\Delta\omega_{\mathrm{n}} = \Delta A_1^{zz} - \Delta A_2^{zz}$ and the nuclear spectral line shape $g_{\mathrm{n}}$, which is predominantly broadened by the hyperfine couplings.

Both Eqs. (S18) and (S20) depend on the convolution of the electron spectral line shape with itself (autoconvolution). Fig. S1 shows a Gaussian electron line width its autoconvolution. The

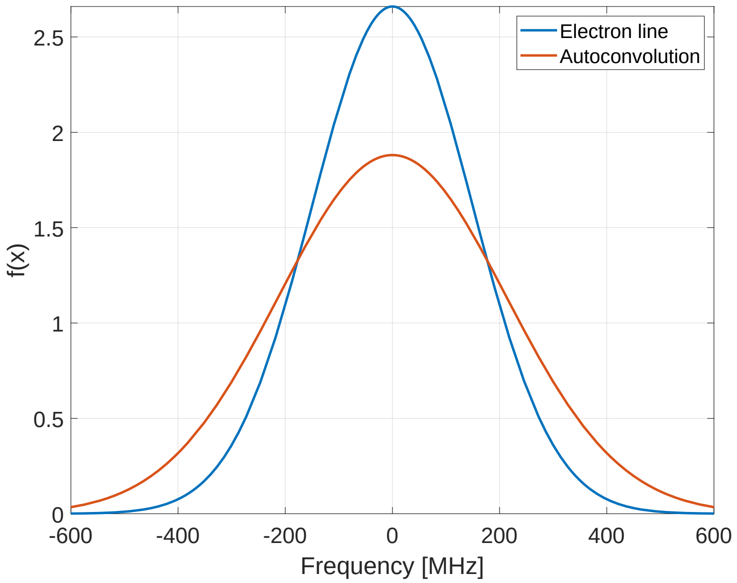


Figure S1: **Gaussian electron line and its autoconvolution.** A Gaussian electron line width 150 MHz broadening and its autoconvolution.

convolution of two Gaussians (normal distribution) is a Gaussian itself broadened by a factor of $\sqrt{2}$. Thus, if the electron line shape provides enough spectral density for EPR detection or solid effect DNP, the $\sqrt{2}$ broader autoconvolution line should provide sufficient spectral density for electronic flip-flops if the frequency difference between the involved electron is similar or smaller than the electron line width.

## S4 Coupled two-compartment model

We start with a recap of the previously introduced single homogeneous compartment model (*49*): The hyperpolarization build-up can be described through a first-order differential equation with a hyperpolarization injection rate constant $k_{\mathrm{W}}$ and a relaxation rate constant of the build-up $k_{\mathrm{R}}^{\mathrm{bup}}$

$$\frac{\mathrm{d}P}{\mathrm{d}t} = (A - P)k_{\mathrm{W}} - k_{\mathrm{R}}^{\mathrm{bup}} P \tag{S21}$$

with $A$ describing the theoretical maximum of hyperpolarization achievable, i.e., the thermal electron polarization in DNP. The solution of Eq. (S21) is a mono-exponential curve which can be compared with the phenomenological description of the build-up curve by $P(t) = P_0(1 - e^{-t/\tau_{\mathrm{bup}}})$ to express the experimental parameters in terms of model parameters. Here, $P_0$ is the steady-state polarization and $\tau_{\mathrm{bup}}$ the build-up time.

$$\tau_{\mathrm{bup}}^{-1} = k_{\mathrm{W}} + k_{\mathrm{R}}^{\mathrm{bup}} \tag{S22a}$$

$$P_0 = \frac{A k_{\mathrm{W}}}{k_W + k_{\mathrm{R}}^{\mathrm{bup}}} = A k_{\mathrm{W}} \tau_{\mathrm{bup}} \tag{S22b}$$

For the decay, $k_{\mathrm{W}}$ would be set to zero (MW off), leading to $\tau_{\mathrm{decay}}^{-1} = k_{\mathrm{R}}^{\mathrm{decay}}$.

Extending the one-compartment model to two uncoupled compartments with separate injection and relaxation rates is straightforward. Such a situation might be realized for a material consisting of two phases with different compositions (radical concentration, NMR-active spin concentration) such that each compartment follows its own mono-exponential build-up (cf. Eqs. (S21), (S22)). Crucially, spin diffusion between the two compartments needs to be suppressed, e.g., through a resonance frequency difference rendering inter-compartment nuclear flip-flops energy non-conserving. If the frequency difference between the two compartments is small compared to the NMR linewidth such that the two compartments cannot be clearly

discriminated through different peaks, the total measured signal describes the total magnetization created in the two compartments. In such a case, the resulting build-up would take a bi-exponential form

$$
\begin{aligned}
P &= P_{0,2}\left[\alpha\left(1-e^{-t/\tau_1}\right)+(1-\alpha)\left(1-e^{-t/\tau_2}\right)\right] \\
&= P_{0,2}\left[1-\alpha e^{-t/\tau_1}-(1-\alpha)e^{-t/\tau_2}\right] \qquad \text{(S23)}
\end{aligned}
$$

with the relative weight of the two time constants $\alpha$. Experimentally, four parameters are extracted from the build-up while in the theoretical model five parameters are required: two injection and relaxation rates each giving rise to the two steady-state polarizations and build-up times as well as the relative size of the compartments. Hence, for two uncoupled compartments, it is difficult to extract information about the individual compartments based on the above compartment model ansatz.

Hence, we focus on a coupled two-compartment model below: Fig. 1 sketches the basic idea of the model with DNP injection only into the first compartment (relative size $\xi$), a coupling between the two compartments and a separate relaxation rate constant for each compartment. The resulting coupled differential equation system takes the form

$$
\frac{\mathrm{d}P_1}{\mathrm{d}t} = (A-P_1)k_{W1} - k_{\mathrm{R}1}P_1 - \frac{k_\eta}{\xi}(P_1-P_2) \qquad \text{(S24a)}
$$

$$
\frac{\mathrm{d}P_2}{\mathrm{d}t} = -k_{\mathrm{R}2}P_2 + \frac{k_\eta}{1-\xi}(P_1-P_2) \qquad \text{(S24b)}
$$

with $P_1$, $P_2$, $k_{\mathrm{R}1}$ and $k_{\mathrm{R}1}$ being the polarizations and relaxation rates of the two compartments, respectively. DNP injection is only possible into the first compartment through the term $(A-P_1)k_{W1}$ with $k_{W1}$ being the DNP injection parameter. $A$ describes the theoretical maximum polarization, e.g., the thermal electron polarization in DNP. The idea behind this term

is discussed in more detail in (*49*). $k_\eta$ is the inter-compartment coupling parameter. $\xi$ defines the relative size of the two compartments. We note that the model can viewed as a generalization of the two-compartment model in (*8*) for build-ups and decays ($k_{W1} = 0$ and a non-zero starting polarization for decays) although it was intended to shed light on bi-exponential build-ups, e.g., as observed in silicon (*73, 74*). For simplicity and in analogy to the one-compartment model (*49*), we ignored a thermal equilibrium polarization as enhancements of 100 over the thermal equilibrium can be achieved in many materials, rendering the thermal polarization small compared to typical measurement uncertainties.

The coupling parameter $k_\eta$ in Eqs. (S24) is modulated by the compartment size $\xi$. $k_\eta$ describes a magnetization exchange between the two compartments and a large magnetization added to a small system leads to a drastic change of the polarization of the compartment as the polarization is a normalized magnetization. The total polarization of the system would be described by $P = \xi P_1 + (1 - \xi)P_2$.

The structure of the coupling $(P_1 - P_2)$ rather describes a polarization gradient and, hence, can be interpreted as a polarization flux according to Fick's first law of diffusion. Nuclear spin polarization usually spreads in a diffusive way (Fick's second law of diffusion):

$$\frac{\partial P}{\partial t} = \nabla \left[ D(x) \nabla P \right] = D \Delta P \tag{S25}$$

where an isotropic spin diffusion coefficient $D$ is assumed in the last step. $\Delta$ is the Laplace operator. Since our two-compartment model (see Fig. 1 and Eqs. (S24)) is independent of spatial variables, the Laplace operator is not defined, leaving us with the above two-compartment model. The spatially-dependent case (Eq. (S25)) including relaxation and DNP injection has been solved in a numerically efficient way by Pinon and co-workers (*75*) and successfully applied to understand several complex materials better (*76–79*). The spin diffusion model by

Pinon and co-workers is rather focused on understanding the microscopic details, whereas the two-coupled compartment model is very macroscopic but offering intuitive understanding. Hence, the discussion of this model should give some general understanding of relationships between injection rate, relaxation and coupling on the one side and experimentally measured parameters on the other.

For simplicity, we first solve the coupled differential equation system with both compartments having the same size ($\xi = 0.5$) as this eliminates $\xi$s from the equations and with it simplifying the notation. Extension to the general case with arbitrary $\xi$ is straightforward. Thus, we start from

$$\frac{\mathrm{d}P_1}{\mathrm{d}t} = (A - P_1)k_{W1} - k_{\mathrm{R1}}P_1 - k_\eta(P_1 - P_2) \tag{S26a}$$
$$\frac{\mathrm{d}P_2}{\mathrm{d}t} = -k_{\mathrm{R2}}P_2 + k_\eta(P_1 - P_2) \tag{S26b}$$

with the same parameters as in the main part except for assuming $\xi = 0.5$ such that the two compartments have equal sizes.

We start by solving the differential equation system by rewriting the second equation. (S26b), to

$$P_1 = \frac{\mathrm{d}P_2}{\mathrm{d}t} + k_{\mathrm{R2}}P_2 + k_\eta P_2 \tag{S27}$$

and inserting this into the first equation, (S26a) to have a differential equation for $P_2$. We make and exponential ansatz $e^{rt}$ to solve the homogenous equation which gives us a polynomial equation for $r$ which we can easily solve to

$$r_{1/2} = -\frac{1}{2}\left(k_{\mathrm{R1}} + k_{\mathrm{R2}} + k_{W1} + 2k_\eta\right) \pm \frac{1}{2}\sqrt{\left(k_{\mathrm{R1}} - k_{\mathrm{R2}} + k_{W1}\right)^2 + 4k_\eta^2} \tag{S28}$$

From the inhomogenous case we find $C_0 = \frac{(k_{\mathrm{R2}}+k_\eta)Ak_{W1}}{(k_{W1}+k_{\mathrm{R1}}+k_\eta)(k_{\mathrm{R2}}+k_\eta)-k_\eta^2}$. We now can insert our solution for $P_2$ into the second equation, (S26b), to find our solution for $P_1$. Our total polariza-

tion, which we measure experimentally, is given by $P = (P_1 + P_2)/2$ as we have to average over the two compartments, and, thus, we find

$$\begin{aligned} P =& \frac{c_1}{2}\left(1 + \frac{r_1 + k_{W1} + k_{\mathrm{R1}} + k_\eta}{k_\eta}\right) e^{r_1 t} + \frac{c_2}{2}\left(1 + \frac{r_2 + k_{W1} + k_{\mathrm{R1}} + k_\eta}{k_\eta}\right) e^{r_2 t} + ... \\ &+ \frac{1}{2}\frac{(k_{\mathrm{R2}} + 2k_\eta)Ak_{W1}}{(k_{W1} + k_{\mathrm{R1}} + k_\eta)(k_{\mathrm{R2}} + k_\eta) - k_\eta^2} \end{aligned} \tag{S29}$$

with $c_1$ and $c_2$ being constants of integration. We can set these by comparing our solution with an experimentally used model, e.g., a bi-exponential build-up (cf. (S23))

$$\begin{aligned} P &= P_{0,2}\left[\alpha\left(1 - e^{-t/\tau_1}\right) + (1-\alpha)\left(1 - e^{-t/\tau_2}\right)\right] \\ &= P_{0,2}\left[1 - \alpha e^{-t/\tau_1} - (1-\alpha)e^{-t/\tau_2}\right] \end{aligned} \tag{S30}$$

for which we dropped the "bup" subscript for convenience. We can immediately read off

$$P_{0,2} = \frac{1}{2}\frac{(k_{\mathrm{R2}} + 2k_\eta)Ak_{W1}}{(k_{W1} + k_{\mathrm{R1}} + k_\eta)(k_{\mathrm{R2}} + k_\eta) - k_\eta^2} \tag{S31a}$$

$$\tau_1^{-1} = -r_1 \tag{S31b}$$

$$\tau_2^{-1} = -r_2 \tag{S31c}$$

Furthermore, we can choose $c_1$ and $c_2$ such that it reproduces the $-\alpha$ and $-(1-\alpha)$ prefactors of the exponentials. Our fourth equation for the theory-experimental correspondence comes from

$$\frac{\mathrm{d}P_1}{\mathrm{d}t}(0) = -r_1\alpha P_{0,2} = Ak_{W1} \tag{S32}$$

as we assume both compartments to be completely unpolarized initially. With these four equations at hand, we could write the four theory parameters in terms of the four experimental parameters. However, this provides little insight as we encounter some complicated complex-valued fourth-order polynomial equations stemming from the square roots in the time constants. Thus, we take a different approach and find the parameter correspondence through analysis of the coupled differential equation system in the steady-state as well as boundary conditions.

For large times our system is in a steady-state with the total polarization being $P_{0,2}$ and experimentally the two compartments having a polarization equal to $P_{1s} = 2\alpha P_{0,2}$ and $P_{2s} = 2(1-\alpha)P_{0,2}$. From equation (S26b) we find for large times

$$k_\eta(P_{1s} - P_{2s}) = k_{\mathrm{R2}}P_{2s} \Leftrightarrow k_{\mathrm{R2}} = k_\eta \frac{\alpha - (1-\alpha)}{1-\alpha} \tag{S33a}$$

which restrains $\alpha$ to be larger than $0.5$ to ensure that $k_{\mathrm{R2}} \geq 0$. Additionally, we notice that equation (S26b) is for large times identical to the one compartment rate equation discussed in (*49*) $\left(\frac{\mathrm{d}P}{\mathrm{d}t} = (A-P)k_{\mathrm{W}} - k_{\mathrm{R}}P\right)$ upon substitution of $P_{1s}$ with $A$ and $k_\eta$ with $k_{W1}$. Thus, we find

$$P_{2s} = k_\eta P_{1s}\tau_2 \Leftrightarrow \eta = \frac{1-\alpha}{\alpha}\tau_2^{-1} \tag{S34a}$$

$$\Rightarrow k_{\mathrm{R2}} = \tau_2^{-1}\left(2 - \alpha^{-1}\right) \tag{S34b}$$

Again we can use

$$\frac{\mathrm{d}P_1}{\mathrm{d}t}(0) = \alpha P_{0,2}\tau_1^{-1} = Ak_{W1} \Leftrightarrow k_{W1} = \frac{P_{0,2}}{A}\alpha\tau_1^{-1} \tag{S35}$$

For the relaxation rate of the first compartment we can use the steady-state condition of the overall system

$$(A - P_{1s})k_{W1} = k_{\mathrm{R1}}P_{1s} + k_{\mathrm{R2}}P_{2s} \tag{S36a}$$

$$k_{\mathrm{R1}} = k_{W1}\left(\frac{A}{2\alpha P_{0,2}} - 1\right) - \frac{1-\alpha}{\alpha}R_2 \tag{S36b}$$

We note that inserting these parameters into the exact solution for the time constants of the coupled differential equation system, equation (S28), does not reproduce the experimental time constants exactly. Such a discrepancy was to be expected as the differential equation system is not describing the experimentally occurring spin diffusion mediated build-up of polarization but rather describes a spin flux. A comparison between the experimental model and its corresponding theoretical build-up is shown in Fig. S2a. The discrepancy of the theoretical model is

due to the structure of our coupling between the two compartments. We first need to build up a polarization difference between the two compartments before polarization can be transferred into the second compartment. This leads to a very slow initial build-up compared to the experimental model. Furthermore, this acts back on the first compartment as this is drained by the large polarization difference once it developed, leading to a slower build-up of polarization in the first compartment. Interestingly, once we fit the total polarization of our theoretical model, the faster time constant is smaller than in the experimental case but at the expense of a smaller balance parameter $\alpha$. This is a result of the internal interaction between the two compartments and fitting them jointly. Interestingly, the build-up of polarization within each compartment cannot be fitted well with a mono-exponential as shown in Fig. S2a whereas the total polarization can be fitted accurately with a bi-exponential as given by equation (S30), underlining that the description of the exchange of polarization between the two compartments in our model is not trivial.

We can find the same parameters for the experiment-theory correspondence with an alternative approach. For this we solve our coupled differential equation ((S26a)-(S26b)) under the approximation that $P_1$ in the differential equation for $P_2$ ((S26b)) is time-independent and vice versa. This is similar to the Born-Oppenheimer approximation in quantum chemistry where the dynamics of electrons and nuclei is assumed to be at different time scales such that the electrons experience the nuclei as being at rest and vice versa. If the two time scales in the build-up are vastly different, this assumption would be valid as the polarization in the first compartment is already fully developed while the second compartment is still nearly unpolarized. This is shown and discussed in Fig. S2. In practice, this assumption will not fully hold as it is difficult to observe a component that builds-up orders of magnitude slower than the other.

We start solving the differential equation system by first solving the second equation ((S26b)).

This gives us

$$P_2 = \frac{\eta}{k_{\mathrm{R2}} + k_\eta} P_1 + c_2 e^{-(k_{\mathrm{R2}}+k_\eta)t} \tag{S37}$$

We can insert this into the first equation (Eq. (S26a)) and get

$$\begin{aligned} P_1 =& \frac{A k_{W1}}{k_{W1} + k_{\mathrm{R1}} + k_\eta - \frac{k_\eta^2}{k_{\mathrm{R2}}+k_\eta}} + c_1 e^{-\left(k_{W1}+k_{\mathrm{R1}}+k_\eta - \frac{k_\eta^2}{k_{\mathrm{R2}}+k_\eta}\right)t} + \\ & \frac{k_\eta}{k_{W1} + k_{\mathrm{R1}} - k_{\mathrm{R2}} - \frac{k_\eta^2}{k_{\mathrm{R2}}+k_\eta}} c_2 e^{-(k_{\mathrm{R2}}+k_\eta)t} \end{aligned} \tag{S38}$$

The total polarization $P$ of the system, as measured experimentally, is given by the weighted

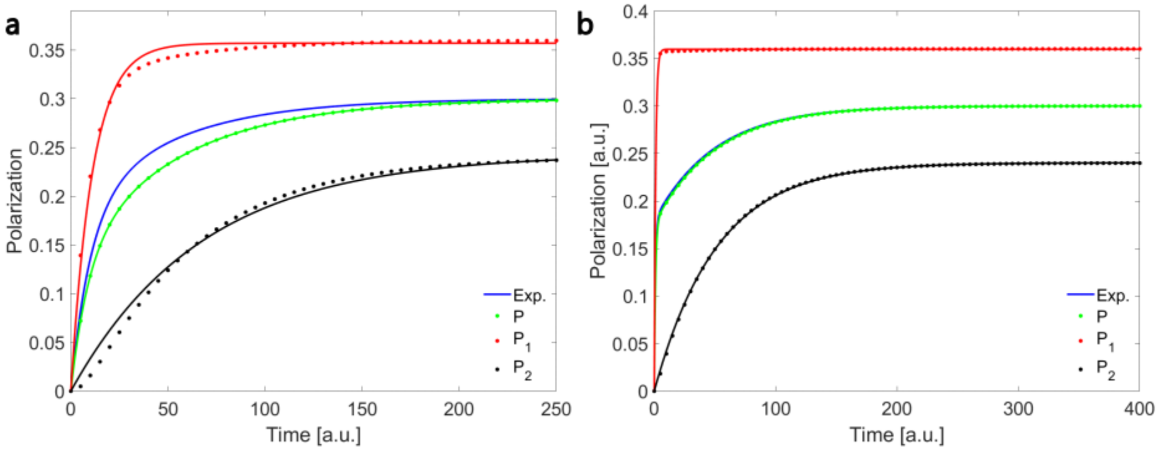


Figure S2: **Simulated two-compartment build-ups.** The differential equation model reproduces the experimental bi-exponential build-up for vastly different time constants between the two compartments but leads to imperfections for time constants on the same order of magnitude. (a) Simulation parameters: $P_{0,2} = 0.3$, $\tau_1 = 1$, $\tau_2 = 50$, $\alpha = 0.6$, $A = 1$, $\xi = 0.5$. Fitting the bi-exponential build-up resulting from numerical integration of the differential equation system gives $P_{0,2} = 0.3$, $\alpha = 0.4523$, $\tau_1 = 9.00$, $\tau_2 = 55.41$. Note that the polarizations of the individual compartments cannot be fitted by mono-exponential models due to the interaction between the two. (b) Setting the fast build-up time to 1, leads to an excellent agreement between the theoretical and experimental model. The assumption that the two build-ups are at different time scales such that one is always at equilibrium on the time scale of the other, can be considered fulfilled in this case. The slow second compartment builds up exponentially as the initial delay to build up the polarization difference $P_1 - P_2$ is negligible. Simulation parameters: $P_0 = 0.3$, $\tau_1 = 1$, $\tau_2 = 50$, $\alpha = 0.6$, $A = 1$, $\xi = 0.5$

sum of $P_1$ and $P_2$.

$$P = \frac{P_1 + P_2}{2} = \frac{1}{2}\left(1 + \frac{k_\eta}{k_{\mathrm{R2}} + k_\eta}\right) P_1 + \frac{c_2}{2} e^{-(k_{\mathrm{R2}}+k_\eta)t}$$

$$= \frac{1}{2}\left(1 + \frac{k_\eta}{k_{\mathrm{R2}} + k_\eta}\right)\left[\frac{Ak_{W1}}{k_{W1} + k_{\mathrm{R1}} + k_\eta - \frac{k_\eta^2}{k_{\mathrm{R2}}+k_\eta}} + \frac{c_1}{2} e^{-\left(k_{W1}+k_{\mathrm{R1}}+k_\eta-\frac{k_\eta^2}{k_{\mathrm{R2}}+k_\eta}\right)t}\right] + ...$$

$$+ \left[1 + \frac{k_\eta}{k_{W1} + k_{\mathrm{R1}} - k_{\mathrm{R2}} - \frac{k_\eta^2}{k_{\mathrm{R2}}+k_\eta}}\right] \frac{c_2}{2} e^{-(k_{\mathrm{R2}}+k_\eta)t} \tag{S39a}$$

$$= \frac{1}{2}\left(1 + \frac{k_\eta}{k_{\mathrm{R2}} + k_\eta}\right) \frac{Ak_{W1}}{k_{W1} + k_{\mathrm{R2}} + k_\eta - \frac{k_\eta^2}{k_{\mathrm{R2}}+k_\eta}} \left[1 + \frac{k_{W1} + k_{\mathrm{R1}} + k_\eta - \frac{k_\eta^2}{k_{\mathrm{R2}}+k_\eta}}{Ak_{W1}} ...\right.$$

$$\left[c_1 e^{-\left(k_{W1}+k_{\mathrm{R1}}+k_\eta-\frac{k_\eta^2}{k_{\mathrm{R2}}+k_\eta}\right)t} + \left(1 + \frac{k_\eta}{k_{\mathrm{R2}} + k_\eta}\right)^{-1} ...\right.$$

$$\left.\left.\left(1 + \frac{k_\eta}{k_{W1} + k_{\mathrm{R1}} - k_{\mathrm{R2}} - \frac{k_\eta^2}{k_{\mathrm{R2}}+k_\eta}}\right) c_2 e^{-(k_{\mathrm{R2}}+k_\eta)t}\right]\right] \tag{S39b}$$

To find the constants of integration, we rewrite equation (S39b)

$$P = P_{0,2}\left[1 - \frac{1}{Ak_{W1}\tau_1}\left[c_1 e^{-t/\tau_1} + \frac{1}{1 + k_\eta\tau_2}\left(1 + \frac{k_\eta}{\tau_1^{-1} - \tau_2^{-1}}\right) c_2 e^{-t/\tau_2}\right]\right] \tag{S40}$$

and can find as a boundary condition for large $t$

$$\frac{1}{Ak_{W1}\tau_1}\left[\frac{1}{1 + k_\eta\tau_2}\left(1 + \frac{k_\eta}{\tau_1^{-1} - \tau_2^{-1}}\right) c_2 + c_1\right] = -(1-\alpha) - \alpha = -1 \tag{S41}$$

and choose

$$\alpha = \frac{c_1}{Ak_{W1}\tau_1} \tag{S42a}$$

$$-(1-\alpha) = \frac{c_2}{Ak_{W1}\tau_1} \frac{1}{1 + k_\eta\tau_2}\left(1 + \frac{k_\eta}{\tau_1^{-1} - \tau_2^{-1}}\right) \tag{S42b}$$

If we compare (S39b) with our bi-exponential, experimental model (Eq. (S30)), we can read

off equations for both time constants and the steady-state polarization $P_{0,2}$.

$$\tau_1^{-1} = k_{W1} + k_{\mathrm{R}1} + k_\eta - \frac{k_\eta^2}{k_{\mathrm{R}2} + k_\eta} \tag{S43a}$$

$$\tau_2^{-1} = k_{\mathrm{R}2} + k_\eta \tag{S43b}$$

$$P_{0,2} = \frac{1}{2}\left(1 + \frac{k_\eta}{k_{\mathrm{R}2} + k_\eta}\right) \frac{A k_{W1}}{k_{W1} + k_{\mathrm{R}1} + k_\eta - \frac{k_\eta^2}{k_{\mathrm{R}2}+k_\eta}} = \frac{1}{2}(1 + k_\eta \tau_2) A k_{W1} \tau_1 \tag{S43c}$$

$$\alpha = (1 + k_\eta \tau_2)^{-1} \tag{S43d}$$

We can rewrite these four equations to

$$k_{\mathrm{R}1} = \tau_1^{-1} - k_{W1} - k_\eta + k_\eta^2 \tau_2 \tag{S44a}$$

$$k_{\mathrm{R}2} = \tau_2^{-1} - k_\eta \tag{S44b}$$

$$k_{W1} = \frac{2P_{0,2}}{(1 + k_\eta \tau_2) A \tau_1} \tag{S44c}$$

$$k_\eta = \frac{1-\alpha}{\alpha} \tau_2^{-1} \tag{S44d}$$

where we used the same arguments for $k_\eta$ as for equation (S34a). These four equations give the same differential equation parameters as our above approach with the boundary conditions and the steady-state analysis of the differential equation system.

From here it is straightforward to include the size of the first compartment $\xi$ resulting in

$$\alpha = \left(1 + \frac{k_\eta}{\xi}\tau_2\right)^{-1} \tag{S45a}$$

$$\tau_2^{-1} = k_{\mathrm{R}2} + \frac{k_\eta}{1-\xi} \tag{S45b}$$

$$\begin{aligned} \tau_1^{-1} &= k_{W1} + k_{\mathrm{R}1} + \frac{k_\eta}{\xi} - \frac{k_\eta^2}{\xi(1-\xi)}\left(k_{\mathrm{R}2} + \frac{k_\eta}{1-\xi}\right)^{-1} \\ &= k_{W1} + k_{\mathrm{R}1} + \frac{k_\eta}{\xi} - \frac{k_\eta^2}{\xi(1-\xi)}\tau_2 \end{aligned} \tag{S45c}$$

$$\begin{aligned} P_{0,2} &= \frac{A k_{W1}\left[\xi + k_\eta \left(k_{\mathrm{R}2} + \frac{k_\eta}{1-\xi}\right)^{-1}\right]}{k_{W1} + k_{\mathrm{R}1} + \frac{k_\eta}{\xi} - \frac{k_\eta^2}{\xi(1-\xi)}\left(k_{\mathrm{R}2} + \frac{k_\eta}{1-\xi}\right)^{-1}} \\ &= A k_{W1} \tau_1 \left[\xi + k_\eta \tau_2\right] \quad . \end{aligned} \tag{S45d}$$

For the decay, the injection term from equation (S26a) is eliminated. If we follow the above approach with different time scales of the compartments, the time constants are identical to the build-up case apart from the vanishing $k_{W1}$. Under the assumption of long build-up times before the decay such that both compartments reach their steady-state polarization, the initial polarization and coupling constant are the same as for the build-up. The exact solution for the decay case is given in (*8*) to understand the interaction between hypershifted spins with the RF-visible bulk.

# S5 Two compartment HypRes-on fits

The simulations shown in Figs. 3 and S1 were performed with MATLAB. For the simulations of the HypRes-on (MW-on HypRes experiments) data from (*9*), provided to us from Quentin Stern, we numerically integrated Eqs. (S24) (time slicing) and performed a grid search over 1 million iterations after pre-scanning the parameter ranges. The model is dominated by two competing processes: (i) Transport of polarization stored in the hypershifted spins with compartment size $\xi$ into the RF saturated bulk and (ii) DNP injection of the other DNP lobe with respect to the lobe used for the initial polarization build-up. For this, we set the polarization of the hidden compartment ($P_1$) to the estimated build-up polarization of 70%. Since the experiments were performed at 7 T and 1.2 K, we set $A = -1$ (the minus sign stems from the choice of the DNP lobe). We assumed all relaxation being mediated by the electrons, hence, the relaxation of the second compartment $k_{\mathrm{R}2}$ was set to zero (in agreement with the data from (*8*)). The model parameters being varied in the grid search were initially the DNP injection parameter $k_{W1}$, relaxation rate $k_{\mathrm{R}1}$ and the size of the first hidden compartment $\xi$ as well as the inter-compartment coupling parameter $k_\eta$ (compare Eqs. (S24)). The model was found to be insensitive to any relaxation, likely due to the short experimental build-up duration of 25 s. Thus, we set $k_{\mathrm{R}1} = 0$ for the simulations shown in this work. In a similar way, the first compartment size $\xi$ was initially used as a fit parameter but values were around 7% and thus fixed to 7% to simplify the final fits with finer parameter grids. The parameter combinations with the lowest least square value are chosen and shown in Fig. S1. The polarization of the second compartment at the beginning of the simulation (time zero) was set to zero, explaining the discrepancies for very short times. The best fit parameters are summarized in Fig. 3.

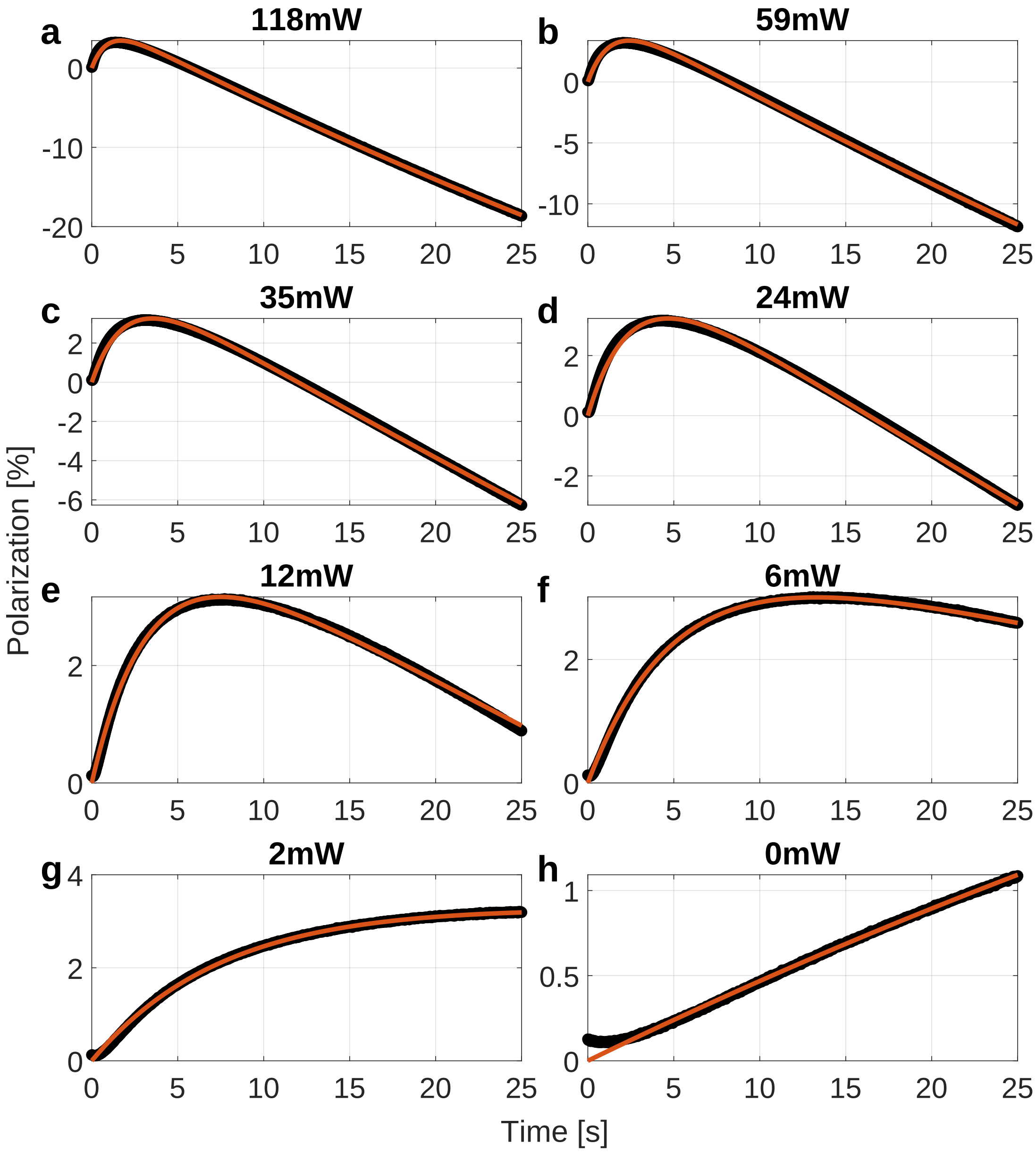


Figure S3: **Simulated and experimental HypRes-on data** Simulated HypRes-on in red and experimental data from (*9*) in black.

## S6 Model for the electron saturation

To fit the best model parameters as shown in Fig. 3, we adopted the Torrey model (*82*) of damped Rabi oscillations (spin Rabi oscillations in the presence of relaxation) to describe the reduced electron polarization under MW irradiation (partial saturation of the electrons). For long time scales as in CW (continuous wave) DNP build-ups, only the time-independent terms need to be considered, although other terms might play a role in non-CW MW irradiation which potentially could result in higher electron recruitment. The time-independent part of the Torrey model describing the ratio between the electron polarization at infinite time under MW irradiation ($P_{\mathrm{e},\infty}$) to the thermal electron polarization of the system after rewriting from (*82*) takes the form

$$\frac{P_{\mathrm{e},\infty}}{P_{0,\mathrm{e}}} = \frac{\left(1-\frac{\omega_{\mathrm{MW}}}{\omega_{0,\mathrm{e}}}\right)^2 \gamma_{\mathrm{e}}^2 B_{1,\mathrm{MW}}^2 T_{2,\mathrm{e}}^2 + 1}{\gamma_{\mathrm{e}}^2 B_{1,\mathrm{MW}}^2 T_{2,\mathrm{e}} \left(\left(1-\frac{\omega_{\mathrm{MW}}}{\omega_{0,\mathrm{e}}}\right)^2 T_{2,\mathrm{e}} + T_{1,\mathrm{e}}\right) + 1} \tag{S46}$$

with the electron gyromagnetic ratio $\gamma_e$, its associated resonance frequency $\omega_{0,\mathrm{e}} = \gamma_e B_0$, relaxation times $T_{1,\mathrm{e}}$ and $T_{2,\mathrm{e}}$ as well as the MW frequency $\omega_{\mathrm{MW}}$ and the MW field strength $B_{1,\mathrm{MW}}$. The resulting expression is identical to the steady-state solution of the z-magnetization of the Bloch equations for the electrons. Electron spectral diffusion and electron line broadening are not explicitly included in the approach but might give rise to electron relaxation times differing from those observed in typical EPR measurements.

Since DNP employs (near-) resonant MW irradiation and $T_{2,\mathrm{e}} \ll T_{1,\mathrm{e}}$ at low temperatures and high electron concentrations, Eq. (S46) can be simplified to

$$1 - \frac{P_{\mathrm{e},\infty}}{P_{0,\mathrm{e}}} = 1 - \frac{1}{\gamma_{\mathrm{e}}^2 B_{1,\mathrm{MW}}^2 T_{2,\mathrm{e}} T_{1,\mathrm{e}} + 1} \tag{S47}$$

# S7 Mathematica notebook of two-electron two-nucleus four-spin system

The Schrieffer-Wolff transformations discussed in this work were computed with Mathamatica. The Mathematica notebook of the lab frame two-electron two-nucleus four-spin system is found below. Mathematica notebooks for the rotating frame and the one-electron one-nucleus two spin system with MW irradiation were derived from the notebook below.

```
In[•]:= << NMR11/StartUpNMR.m
<< mathematica/NMRmaer.m;
<< Notation`;
```

# NMR with Mathematica

*Version 1.1*

*Marlies Brinksma*

*Email: mabi@solidmr.kun.nl*

© 1998, Marlies Brinksma, Matthias Ernst and Beat Meier, Department of Physical Chemistry, University of Nijmegen, The Netherlands

```
C:\Program Files\Wolfram Research\Mathematica\13.3\AddOns\Applications\NMR11\Spinsystem.m
  loaded...
```

NormalForm: $Pre is set to: CheckTimes

```
C:\Program Files\Wolfram Research\Mathematica\13.3\AddOns\Applications\NMR11\NormalForm.m
  loaded...

C:\Program Files\Wolfram Research\Mathematica\13.3\AddOns\Applications\NMR11\SOFunctions.m
  loaded...
```

SetDelayed: Tag SquareMatrixQ in SquareMatrixQ[mat_?MatrixQ] is Protected.

SetDelayed: Tag SquareMatrixQ in SquareMatrixQ[expr_] is Protected.

```
C:\Program Files\Wolfram Research\Mathematica\13.3\AddOns\Applications\NMR11\Matrix.m
  loaded...

C:\Program Files\Wolfram Research\Mathematica\13.3\AddOns\Applications\NMR11\QMFunctions.m
  loaded...

C:\Program Files\Wolfram Research\Mathematica\13.3\AddOns\Applications\NMR11\NMRFunctions.m
  loaded...
```

Get: Cannot open mathematica/NMRmaer.m.

In[•]:= Symbolize[$\omega_{e2}$] (*$\omega_e$=-$\gamma_e B_0$>0 as $\gamma_e$<0 *)
Symbolize[$\omega_{e1}$] (*$\omega_e$=-$\gamma_e B_0$>0 as $\gamma_e$<0 *)
Symbolize[$\omega_n$] (*$\omega_n$=-$\gamma_n B_0$<0 as $\gamma_n$>0 *)
Symbolize[$D^{++}$]
Symbolize[$D^{+-}$]
Symbolize[$D^{-+}$]
Symbolize[$D^{--}$]
Symbolize[$D^{z-}$]
Symbolize[$D^{z+}$]
Symbolize[$D^{+z}$]
Symbolize[$D^{-z}$]
Symbolize[$D^{zz}$]

Symbolize[$d^{zz}$]
Symbolize[$d^{++}$]
Symbolize[$d^{+-}$]
Symbolize[$d^{-+}$]
Symbolize[$d^{--}$]
Symbolize[$d^{z-}$]
Symbolize[$d^{z+}$]

```
Symbolize[d^{+z}]
Symbolize[d^{-z}]

Symbolize[A_{13}^{++}]
Symbolize[A_{13}^{+-}]
Symbolize[A_{13}^{-+}]
Symbolize[A_{13}^{--}]
Symbolize[A_{13}^{+z}]
Symbolize[A_{13}^{-z}]
Symbolize[A_{13}^{z+}]
Symbolize[A_{13}^{z-}]
Symbolize[A_{13}^{zz}]

Symbolize[A_{23}^{++}]
Symbolize[A_{23}^{+-}]
Symbolize[A_{23}^{-+}]
Symbolize[A_{23}^{--}]
Symbolize[A_{23}^{+z}]
Symbolize[A_{23}^{-z}]
Symbolize[A_{23}^{z+}]
Symbolize[A_{23}^{z-}]
Symbolize[A_{23}^{zz}]

Symbolize[A_{14}^{++}]
Symbolize[A_{14}^{+-}]
Symbolize[A_{14}^{-+}]
Symbolize[A_{14}^{--}]
Symbolize[A_{14}^{+z}]
Symbolize[A_{14}^{-z}]
Symbolize[A_{14}^{z+}]
Symbolize[A_{14}^{z-}]
Symbolize[A_{14}^{zz}]

Symbolize[A_{24}^{++}]
Symbolize[A_{24}^{+-}]
Symbolize[A_{24}^{-+}]
Symbolize[A_{24}^{--}]
Symbolize[A_{24}^{+z}]
Symbolize[A_{24}^{-z}]
Symbolize[A_{24}^{z+}]
Symbolize[A_{24}^{z-}]
Symbolize[A_{24}^{zz}]

Symbolize[A_{13}]
Symbolize[A_{23}]
Symbolize[A_{14}]
Symbolize[A_{24}]
```

```
Symbolize[D_12]
Symbolize[d_34]

Symbolize[S_1]
Symbolize[S_2]
Symbolize[I_3]
Symbolize[I_4]

Symbolize[S_1z]
Symbolize[S_2z]
Symbolize[I_3z]
Symbolize[I_4z]

Symbolize[H_0]
```

QMFunctions: Calculation done in the subspace: {}

In[ ]:=
```
RegisterSpin[{1, 2, 3, 4}, {1 / 2, 1 / 2, 1 / 2, 1 / 2}]
```

Spinsystem: Spin 1 has been registered...

Spinsystem: Spin 2 has been registered...

Spinsystem: Spin 3 has been registered...

General: Further output of Spinsystem::Registered will be suppressed during this calculation.

In[ ]:=
```
SetNucleus[{1, 2, 3, 4}, {"S", "S", "I", "I"}]
```

ima·· imagir

In[ ]:=

$$A_{13} = \{\{A_{13}^{++}, A_{13}^{+-}, A_{13}^{+z}\}, \{A_{13}^{-+}, A_{13}^{--}, A_{13}^{-z}\}, \{A_{13}^{z+}, A_{13}^{z-}, A_{13}^{zz}\}\};$$
$$A_{14} = \{\{A_{14}^{++}, A_{14}^{+-}, A_{14}^{+z}\}, \{A_{14}^{-+}, A_{14}^{--}, A_{14}^{-z}\}, \{A_{14}^{z+}, A_{14}^{z-}, A_{14}^{zz}\}\};$$
$$A_{23} = \{\{A_{23}^{++}, A_{23}^{+-}, A_{23}^{+z}\}, \{A_{23}^{-+}, A_{23}^{--}, A_{23}^{-z}\}, \{A_{23}^{z+}, A_{23}^{z-}, A_{23}^{zz}\}\};$$
$$A_{24} = \{\{A_{24}^{++}, A_{24}^{+-}, A_{24}^{+z}\}, \{A_{24}^{-+}, A_{24}^{--}, A_{24}^{-z}\}, \{A_{24}^{z+}, A_{24}^{z-}, A_{24}^{zz}\}\};$$
$$D_{12} = \{\{D^{++}, D^{+-}, D^{+z}\}, \{D^{-+}, D^{--}, D^{-z}\}, \{D^{z+}, D^{z-}, D^{zz}\}\};$$
$$d_{34} = \{\{d^{++}, d^{+-}, d^{+z}\}, \{d^{-+}, d^{--}, d^{-z}\}, \{d^{z+}, d^{z-}, d^{zz}\}\};$$

In[ ]:=
```
S_1z = {0, 0, S[1, z]};
S_2z = {0, 0, S[2, z]};
I_3z = {0, 0, S[3, z]};
I_4z = {0, 0, S[4, z]};
```

In[ ]:=
```
S_1 = {S[1, "+"], S[1, "-"], S[1, z]};
S_2 = {S[2, "+"], S[2, "-"], S[2, z]};
I_3 = {S[3, "+"], S[3, "-"], S[3, z]};
I_4 = {S[4, "+"], S[4, "-"], S[4, z]};
```

In[ ]:= (* $\omega_e$=-$\gamma_e B_0$>0 as $\gamma_e$<0 and $\omega_n$=-$\gamma_n B_0$<0 as $\gamma_n$>0 throughout this work, requiring a - sign in front of the nuclear Zeeman energy term to have - $\omega_n$ as the ground state energy*)

$$\mathrm{Ham} = \omega_{e1}\, S[1, z] + \omega_{e2}\, S[2, z] - \omega_n\, (S[3, z] + S[4, z]) + S_1 . D_{12} . S_2 + I_3 . d_{34} . I_4 + S_1 . A_{13} . I_3 + S_1 . A_{14} . I_4 + S_2 . A_{23} . I_3 + S_2 . A_{24} . I_4$$

Out[ ]=

$$\begin{aligned}
&\omega_{e1} S_{1,z} + (D^{--} S_{1,-} + D^{+-} S_{1,+} + D^{z-} S_{1,z})\, S_{2,-} + (D^{-+} S_{1,-} + D^{++} S_{1,+} + D^{z+} S_{1,z})\, S_{2,+} + \\
&\omega_{e2} S_{2,z} + (D^{-z} S_{1,-} + D^{+z} S_{1,+} + D^{zz} S_{1,z})\, S_{2,z} + (A_{13}^{--} S_{1,-} + A_{13}^{+-} S_{1,+} + A_{13}^{z-} S_{1,z})\, I_{3,-} + \\
&(A_{23}^{--} S_{2,-} + A_{23}^{+-} S_{2,+} + A_{23}^{z-} S_{2,z})\, I_{3,-} + (A_{13}^{-+} S_{1,-} + A_{13}^{++} S_{1,+} + A_{13}^{z+} S_{1,z})\, I_{3,+} + \\
&(A_{23}^{-+} S_{2,-} + A_{23}^{++} S_{2,+} + A_{23}^{z+} S_{2,z})\, I_{3,+} + (A_{13}^{-z} S_{1,-} + A_{13}^{+z} S_{1,+} + A_{13}^{zz} S_{1,z})\, I_{3,z} + \\
&(A_{23}^{-z} S_{2,-} + A_{23}^{+z} S_{2,+} + A_{23}^{zz} S_{2,z})\, I_{3,z} + (A_{14}^{--} S_{1,-} + A_{14}^{+-} S_{1,+} + A_{14}^{z-} S_{1,z})\, I_{4,-} + \\
&(A_{24}^{--} S_{2,-} + A_{24}^{+-} S_{2,+} + A_{24}^{z-} S_{2,z})\, I_{4,-} + (d^{--} I_{3,-} + d^{+-} I_{3,+} + d^{z-} I_{3,z})\, I_{4,-} + \\
&(A_{14}^{-+} S_{1,-} + A_{14}^{++} S_{1,+} + A_{14}^{z+} S_{1,z})\, I_{4,+} + (A_{24}^{-+} S_{2,-} + A_{24}^{++} S_{2,+} + A_{24}^{z+} S_{2,z})\, I_{4,+} + \\
&(d^{-+} I_{3,-} + d^{++} I_{3,+} + d^{z+} I_{3,z})\, I_{4,+} + (A_{14}^{-z} S_{1,-} + A_{14}^{+z} S_{1,+} + A_{14}^{zz} S_{1,z})\, I_{4,z} + \\
&(A_{24}^{-z} S_{2,-} + A_{24}^{+z} S_{2,+} + A_{24}^{zz} S_{2,z})\, I_{4,z} + (d^{-z} I_{3,-} + d^{+z} I_{3,+} + d^{zz} I_{3,z})\, I_{4,z} - \omega_n\, (I_{3,z} + I_{4,z})
\end{aligned}$$

In[ ]:=

$$H_0 = \omega_{e1}\, S[1, z] + \omega_{e2}\, S[2, z] - \omega_n\, (S[3, z] + S[4, z]) + S_{1\,z} . D_{12} . S_{2\,z} + I_{3\,z} . d_{34} . I_{4\,z} + S_{1\,z} . A_{13} . I_{3\,z} + S_{1\,z} . A_{14} . I_{4\,z} + S_{2\,z} . A_{23} . I_{3\,z} + S_{2\,z} . A_{24} . I_{4\,z}$$

Out[ ]=

$$\begin{aligned}
&\omega_{e1} S_{1,z} + \omega_{e2} S_{2,z} + D^{zz} S_{1,z} S_{2,z} + A_{13}^{zz} S_{1,z} I_{3,z} + \\
&A_{23}^{zz} S_{2,z} I_{3,z} + A_{14}^{zz} S_{1,z} I_{4,z} + A_{24}^{zz} S_{2,z} I_{4,z} + d^{zz} I_{3,z} I_{4,z} - \omega_n\, (I_{3,z} + I_{4,z})
\end{aligned}$$

In[ ]:=

```
V = Simplify[Ham - H0]
```

vereinfache

Out[ ]=

$$\begin{aligned}
&D^{z-} S_{1,z} S_{2,-} + D^{z+} S_{1,z} S_{2,+} + A_{13}^{z-} S_{1,z} I_{3,-} + A_{23}^{--} S_{2,-} I_{3,-} + A_{23}^{+-} S_{2,+} I_{3,-} + A_{23}^{z-} S_{2,z} I_{3,-} + \\
&A_{13}^{z+} S_{1,z} I_{3,+} + A_{23}^{-+} S_{2,-} I_{3,+} + A_{23}^{++} S_{2,+} I_{3,+} + A_{23}^{z+} S_{2,z} I_{3,+} + A_{23}^{-z} S_{2,-} I_{3,z} + A_{23}^{+z} S_{2,+} I_{3,z} + \\
&A_{14}^{z-} S_{1,z} I_{4,-} + A_{24}^{--} S_{2,-} I_{4,-} + A_{24}^{+-} S_{2,+} I_{4,-} + A_{24}^{z-} S_{2,z} I_{4,-} + d^{--} I_{3,-} I_{4,-} + d^{+-} I_{3,+} I_{4,-} + \\
&d^{z-} I_{3,z} I_{4,-} + A_{14}^{z+} S_{1,z} I_{4,+} + A_{24}^{-+} S_{2,-} I_{4,+} + A_{24}^{++} S_{2,+} I_{4,+} + A_{24}^{z+} S_{2,z} I_{4,+} + d^{-+} I_{3,-} I_{4,+} + \\
&d^{++} I_{3,+} I_{4,+} + d^{z+} I_{3,z} I_{4,+} + A_{24}^{-z} S_{2,-} I_{4,z} + A_{24}^{+z} S_{2,+} I_{4,z} + d^{-z} I_{3,-} I_{4,z} + d^{+z} I_{3,+} I_{4,z} + \\
&S_{1,-}\, (D^{--} S_{2,-} + D^{-+} S_{2,+} + D^{-z} S_{2,z} + A_{13}^{--} I_{3,-} + A_{13}^{-+} I_{3,+} + A_{13}^{-z} I_{3,z} + A_{14}^{--} I_{4,-} + A_{14}^{-+} I_{4,+} + A_{14}^{-z} I_{4,z}) + \\
&S_{1,+}\, (D^{+-} S_{2,-} + D^{++} S_{2,+} + D^{+z} S_{2,z} + A_{13}^{+-} I_{3,-} + A_{13}^{++} I_{3,+} + A_{13}^{+z} I_{3,z} + A_{14}^{+-} I_{4,-} + A_{14}^{++} I_{4,+} + A_{14}^{+z} I_{4,z})
\end{aligned}$$

In[ ]:=

```
VMx = MatrixRepresentation[V, {1, 2, 3, 4}];
```

In[ ]:=

```
H0Mx = MatrixRepresentation[H0, {1, 2, 3, 4}];
```

In[•]:=

```
P = {
    {0, 0, 0, 0, 0, 0, 0, 0, 0, 0, 0, 0, 1, 0, 0, 0},
    {0, 0, 0, 0, 0, 0, 0, 0, 0, 0, 0, 0, 0, 0, 1, 0},
    {0, 0, 0, 0, 0, 0, 0, 0, 0, 0, 0, 0, 0, 1, 0, 0},
    {0, 0, 0, 0, 0, 0, 0, 0, 0, 0, 0, 0, 0, 0, 0, 1},
    {0, 0, 0, 0, 1, 0, 0, 0, 0, 0, 0, 0, 0, 0, 0, 0},
    {0, 0, 0, 0, 0, 0, 1, 0, 0, 0, 0, 0, 0, 0, 0, 0},
    {0, 0, 0, 0, 0, 1, 0, 0, 0, 0, 0, 0, 0, 0, 0, 0},
    {0, 0, 0, 0, 0, 0, 0, 1, 0, 0, 0, 0, 0, 0, 0, 0},
    {0, 0, 0, 0, 0, 0, 0, 0, 0, 1, 0, 0, 0, 0, 0, 0},
    {0, 0, 0, 0, 0, 0, 0, 0, 1, 0, 0, 0, 0, 0, 0, 0},
    {0, 0, 0, 0, 0, 0, 0, 0, 0, 0, 1, 0, 0, 0, 0, 0},
    {0, 0, 0, 0, 0, 0, 0, 0, 0, 0, 0, 1, 0, 0, 0, 0},
    {1, 0, 0, 0, 0, 0, 0, 0, 0, 0, 0, 0, 0, 0, 0, 0},
    {0, 0, 1, 0, 0, 0, 0, 0, 0, 0, 0, 0, 0, 0, 0, 0},
    {0, 1, 0, 0, 0, 0, 0, 0, 0, 0, 0, 0, 0, 0, 0, 0},
    {0, 0, 0, 1, 0, 0, 0, 0, 0, 0, 0, 0, 0, 0, 0, 0}};
Bs = {αααα, αααβ, ααβα, ααββ, αβαα, αβαβ,
    αββα, αβββ, βααα, βααβ, βαβα, βαββ, ββαα, ββαβ, βββα, ββββ};
P.Bs
```

Out[•]=

{ββαα, βββα, ββαβ, ββββ, αβαα, αββα, αβαβ, αβββ, βααβ, βααα, βαβα, βαββ, αααα, ααβα, αααβ, ααββ}

In[•]:=

```
VM = P.VMx.Inverse[P]
```

inverse Matrix

Out[•]=

$$\Big\{\Big\{0, -\frac{A_{13}^{z+}}{2}-\frac{A_{23}^{z+}}{2}+\frac{d^{+z}}{2}, -\frac{A_{14}^{z+}}{2}-\frac{A_{24}^{z+}}{2}+\frac{d^{z+}}{2}, d^{++}, \frac{A_{13}^{-z}}{2}+\frac{A_{14}^{-z}}{2}-\frac{D^{-z}}{2}, A_{13}^{-+}, A_{14}^{-+}, 0, A_{24}^{-+},$$

$$\frac{A_{23}^{-z}}{2}+\frac{A_{24}^{-z}}{2}-\frac{D^{z-}}{2}, A_{23}^{-+}, 0, D^{--}, 0, 0, 0\Big\}, \Big\{-\frac{A_{13}^{z-}}{2}-\frac{A_{23}^{z-}}{2}+\frac{d^{-z}}{2}, 0, d^{-+}, -\frac{A_{14}^{z+}}{2}-\frac{A_{24}^{z+}}{2}-\frac{d^{z+}}{2},$$

$$A_{13}^{--}, -\frac{A_{13}^{-z}}{2}+\frac{A_{14}^{-z}}{2}-\frac{D^{-z}}{2}, 0, A_{14}^{-+}, 0, A_{23}^{--}, -\frac{A_{23}^{-z}}{2}+\frac{A_{24}^{-z}}{2}-\frac{D^{z-}}{2}, A_{24}^{-+}, 0, D^{--}, 0, 0\Big\},$$

$$\Big\{-\frac{A_{14}^{z-}}{2}-\frac{A_{24}^{z-}}{2}+\frac{d^{z-}}{2}, d^{+-}, 0, -\frac{A_{13}^{z+}}{2}-\frac{A_{23}^{z+}}{2}-\frac{d^{+z}}{2}, A_{14}^{--}, 0, \frac{A_{13}^{-z}}{2}-\frac{A_{14}^{-z}}{2}-\frac{D^{-z}}{2}, A_{13}^{-+},$$

$$\frac{A_{23}^{-z}}{2}-\frac{A_{24}^{-z}}{2}-\frac{D^{z-}}{2}, A_{24}^{--}, 0, A_{23}^{-+}, 0, 0, D^{--}, 0\Big\}, \Big\{d^{--}, -\frac{A_{14}^{z-}}{2}-\frac{A_{24}^{z-}}{2}-\frac{d^{z-}}{2}, -\frac{A_{13}^{z-}}{2}-\frac{A_{23}^{z-}}{2}-\frac{d^{-z}}{2},$$

$$0, 0, A_{14}^{--}, A_{13}^{--}, -\frac{A_{13}^{-z}}{2}-\frac{A_{14}^{-z}}{2}-\frac{D^{-z}}{2}, A_{23}^{--}, 0, A_{24}^{--}, -\frac{A_{23}^{-z}}{2}-\frac{A_{24}^{-z}}{2}-\frac{D^{z-}}{2}, 0, 0, 0, D^{--}\Big\},$$

$$\Big\{\frac{A_{13}^{+z}}{2}+\frac{A_{14}^{+z}}{2}-\frac{D^{+z}}{2}, A_{13}^{++}, A_{14}^{++}, 0, 0, \frac{A_{13}^{z+}}{2}-\frac{A_{23}^{z+}}{2}+\frac{d^{+z}}{2}, \frac{A_{14}^{z+}}{2}-\frac{A_{24}^{z+}}{2}+\frac{d^{z+}}{2}, d^{++}, 0, D^{+-}, 0,$$

$$0, \frac{A_{23}^{-z}}{2}+\frac{A_{24}^{-z}}{2}+\frac{D^{z-}}{2}, A_{23}^{-+}, A_{24}^{-+}, 0\Big\}, \Big\{A_{13}^{+-}, -\frac{A_{13}^{+z}}{2}+\frac{A_{14}^{+z}}{2}-\frac{D^{+z}}{2}, 0, A_{14}^{++}, \frac{A_{13}^{z-}}{2}-\frac{A_{23}^{z-}}{2}+\frac{d^{-z}}{2},$$

$$0, d^{-+}, \frac{A_{14}^{z+}}{2}-\frac{A_{24}^{z+}}{2}-\frac{d^{z+}}{2}, 0, 0, D^{+-}, 0, A_{23}^{--}, -\frac{A_{23}^{-z}}{2}+\frac{A_{24}^{-z}}{2}+\frac{D^{z-}}{2}, 0, A_{24}^{-+}\Big\},$$

$$\Big\{A_{14}^{+-}, 0, \frac{A_{13}^{+z}}{2}-\frac{A_{14}^{+z}}{2}-\frac{D^{+z}}{2}, A_{13}^{++}, \frac{A_{14}^{z-}}{2}-\frac{A_{24}^{z-}}{2}+\frac{d^{z-}}{2}, d^{+-}, 0, \frac{A_{13}^{z+}}{2}-\frac{A_{23}^{z+}}{2}-\frac{d^{+z}}{2}, D^{+-},$$

$$0, 0, 0, A_{24}^{--}, 0, \frac{A_{23}^{-z}}{2}-\frac{A_{24}^{-z}}{2}+\frac{D^{z-}}{2}, A_{23}^{-+}\Big\}, \Big\{0, A_{14}^{+-}, A_{13}^{+-}, -\frac{A_{13}^{+z}}{2}-\frac{A_{14}^{+z}}{2}-\frac{D^{+z}}{2}, d^{--},$$

$$\frac{A^{z-}_{14}}{2} - \frac{A^{z-}_{24}}{2} - \frac{d^{z-}}{2}, \frac{A^{z-}_{13}}{2} - \frac{A^{z-}_{23}}{2} - \frac{d^{-z}}{2}, 0, 0, 0, 0, D^{+-}, 0, A^{--}_{24}, A^{--}_{23}, -\frac{A^{-z}_{23}}{2} - \frac{A^{-z}_{24}}{2} + \frac{D^{z-}}{2}\Big\},$$

$$\Big\{A^{+-}_{24}, 0, \frac{A^{+z}_{23}}{2} - \frac{A^{+z}_{24}}{2} - \frac{D^{z+}}{2}, A^{++}_{23}, 0, 0, D^{-+}, 0, 0, -\frac{A^{z-}_{14}}{2} + \frac{A^{z-}_{24}}{2} + \frac{d^{z-}}{2}, d^{+-}, -\frac{A^{z+}_{13}}{2} + \frac{A^{z+}_{23}}{2} - \frac{d^{+z}}{2},$$

$$A^{--}_{14}, 0, \frac{A^{-z}_{13}}{2} - \frac{A^{-z}_{14}}{2} + \frac{D^{-z}}{2}, A^{-+}_{13}\Big\}, \Big\{\frac{A^{+z}_{23}}{2} + \frac{A^{+z}_{24}}{2} - \frac{D^{z+}}{2}, A^{++}_{23}, A^{++}_{24}, 0, D^{-+}, 0, 0, 0,$$

$$-\frac{A^{z+}_{14}}{2} + \frac{A^{z+}_{24}}{2} + \frac{d^{z+}}{2}, 0, -\frac{A^{z+}_{13}}{2} + \frac{A^{z+}_{23}}{2} + \frac{d^{+z}}{2}, d^{++}, \frac{A^{-z}_{13}}{2} + \frac{A^{-z}_{14}}{2} + \frac{D^{-z}}{2}, A^{-+}_{13}, A^{-+}_{14}, 0\Big\},$$

$$\Big\{A^{+-}_{23}, -\frac{A^{+z}_{23}}{2} + \frac{A^{+z}_{24}}{2} - \frac{D^{z+}}{2}, 0, A^{++}_{24}, 0, D^{-+}, 0, 0, d^{-+}, -\frac{A^{z-}_{13}}{2} + \frac{A^{z-}_{23}}{2} + \frac{d^{-z}}{2}, 0, -\frac{A^{z+}_{14}}{2} + \frac{A^{z+}_{24}}{2} - \frac{d^{z+}}{2},$$

$$A^{--}_{13}, -\frac{A^{-z}_{13}}{2} + \frac{A^{-z}_{14}}{2} + \frac{D^{-z}}{2}, 0, A^{-+}_{14}\Big\}, \Big\{0, A^{+-}_{24}, A^{+-}_{23}, -\frac{A^{+z}_{23}}{2} - \frac{A^{+z}_{24}}{2} - \frac{D^{z+}}{2}, 0, 0, 0, D^{-+},$$

$$-\frac{A^{z-}_{13}}{2} + \frac{A^{z-}_{23}}{2} - \frac{d^{-z}}{2}, d^{--}, -\frac{A^{z-}_{14}}{2} + \frac{A^{z-}_{24}}{2} - \frac{d^{z-}}{2}, 0, 0, A^{--}_{14}, A^{--}_{13}, -\frac{A^{-z}_{13}}{2} - \frac{A^{-z}_{14}}{2} + \frac{D^{-z}}{2}\Big\},$$

$$\Big\{D^{++}, 0, 0, 0, \frac{A^{+z}_{23}}{2} + \frac{A^{+z}_{24}}{2} + \frac{D^{z+}}{2}, A^{++}_{23}, A^{++}_{24}, 0, A^{++}_{14}, \frac{A^{+z}_{13}}{2} + \frac{A^{+z}_{14}}{2} + \frac{D^{+z}}{2}, A^{++}_{13}, 0, 0,$$

$$\frac{A^{z+}_{13}}{2} + \frac{A^{z+}_{23}}{2} + \frac{d^{+z}}{2}, \frac{A^{z+}_{14}}{2} + \frac{A^{z+}_{24}}{2} + \frac{d^{z+}}{2}, d^{++}\Big\}, \Big\{0, D^{++}, 0, 0, A^{+-}_{23}, -\frac{A^{+z}_{23}}{2} + \frac{A^{+z}_{24}}{2} + \frac{D^{z+}}{2}, 0,$$

$$A^{++}_{24}, 0, A^{+-}_{13}, -\frac{A^{+z}_{13}}{2} + \frac{A^{+z}_{14}}{2} + \frac{D^{+z}}{2}, A^{++}_{14}, \frac{A^{z-}_{13}}{2} + \frac{A^{z-}_{23}}{2} + \frac{d^{-z}}{2}, 0, d^{-+}, \frac{A^{z+}_{14}}{2} + \frac{A^{z+}_{24}}{2} - \frac{d^{z+}}{2}\Big\},$$

$$\Big\{0, 0, D^{++}, 0, A^{+-}_{24}, 0, \frac{A^{+z}_{23}}{2} - \frac{A^{+z}_{24}}{2} + \frac{D^{z+}}{2}, A^{++}_{23}, \frac{A^{+z}_{13}}{2} - \frac{A^{+z}_{14}}{2} + \frac{D^{+z}}{2}, A^{+-}_{14}, 0, A^{++}_{13},$$

$$\frac{A^{z-}_{14}}{2} + \frac{A^{z-}_{24}}{2} + \frac{d^{z-}}{2}, d^{+-}, 0, \frac{A^{z+}_{13}}{2} + \frac{A^{z+}_{23}}{2} - \frac{d^{+z}}{2}\Big\}, \Big\{0, 0, 0, D^{++}, 0, A^{+-}_{24}, A^{+-}_{23}, -\frac{A^{+z}_{23}}{2} - \frac{A^{+z}_{24}}{2} + \frac{D^{z+}}{2},$$

$$A^{+-}_{13}, 0, A^{+-}_{14}, -\frac{A^{+z}_{13}}{2} - \frac{A^{+z}_{14}}{2} + \frac{D^{+z}}{2}, d^{--}, \frac{A^{z-}_{14}}{2} + \frac{A^{z-}_{24}}{2} - \frac{d^{z-}}{2}, \frac{A^{z-}_{13}}{2} + \frac{A^{z-}_{23}}{2} - \frac{d^{-z}}{2}, 0\Big\}\Big\}$$

In[∘]:= **H0M = P.H0Mx.Inverse[P]**

inverse Matrix

Out[ ]=

$$\left\{\left\{-\frac{A^{zz}_{13}}{4}-\frac{A^{zz}_{14}}{4}-\frac{A^{zz}_{23}}{4}-\frac{A^{zz}_{24}}{4}+\frac{d^{zz}}{4}+\frac{D^{zz}}{4}-\frac{\omega_{e1}}{2}-\frac{\omega_{e2}}{2}-\omega_n,0,0,0,0,0,0,0,0,0,0,0,0,0,0,0\right\},\right.$$

$$\left\{0,\frac{A^{zz}_{13}}{4}-\frac{A^{zz}_{14}}{4}+\frac{A^{zz}_{23}}{4}-\frac{A^{zz}_{24}}{4}-\frac{d^{zz}}{4}+\frac{D^{zz}}{4}-\frac{\omega_{e1}}{2}-\frac{\omega_{e2}}{2},0,0,0,0,0,0,0,0,0,0,0,0,0,0\right\},$$

$$\left\{0,0,-\frac{A^{zz}_{13}}{4}+\frac{A^{zz}_{14}}{4}-\frac{A^{zz}_{23}}{4}+\frac{A^{zz}_{24}}{4}-\frac{d^{zz}}{4}+\frac{D^{zz}}{4}-\frac{\omega_{e1}}{2}-\frac{\omega_{e2}}{2},0,0,0,0,0,0,0,0,0,0,0,0,0\right\},$$

$$\left\{0,0,0,\frac{A^{zz}_{13}}{4}+\frac{A^{zz}_{14}}{4}+\frac{A^{zz}_{23}}{4}+\frac{A^{zz}_{24}}{4}+\frac{d^{zz}}{4}+\frac{D^{zz}}{4}-\frac{\omega_{e1}}{2}-\frac{\omega_{e2}}{2}+\omega_n,0,0,0,0,0,0,0,0,0,0,0,0\right\},$$

$$\left\{0,0,0,0,\frac{A^{zz}_{13}}{4}+\frac{A^{zz}_{14}}{4}-\frac{A^{zz}_{23}}{4}-\frac{A^{zz}_{24}}{4}+\frac{d^{zz}}{4}-\frac{D^{zz}}{4}+\frac{\omega_{e1}}{2}-\frac{\omega_{e2}}{2}-\omega_n,0,0,0,0,0,0,0,0,0,0,0\right\},$$

$$\left\{0,0,0,0,0,-\frac{A^{zz}_{13}}{4}+\frac{A^{zz}_{14}}{4}+\frac{A^{zz}_{23}}{4}-\frac{A^{zz}_{24}}{4}-\frac{d^{zz}}{4}-\frac{D^{zz}}{4}+\frac{\omega_{e1}}{2}-\frac{\omega_{e2}}{2},0,0,0,0,0,0,0,0,0,0\right\},$$

$$\left\{0,0,0,0,0,0,\frac{A^{zz}_{13}}{4}-\frac{A^{zz}_{14}}{4}-\frac{A^{zz}_{23}}{4}+\frac{A^{zz}_{24}}{4}-\frac{d^{zz}}{4}-\frac{D^{zz}}{4}+\frac{\omega_{e1}}{2}-\frac{\omega_{e2}}{2},0,0,0,0,0,0,0,0,0\right\},$$

$$\left\{0,0,0,0,0,0,0,-\frac{A^{zz}_{13}}{4}-\frac{A^{zz}_{14}}{4}+\frac{A^{zz}_{23}}{4}+\frac{A^{zz}_{24}}{4}+\frac{d^{zz}}{4}-\frac{D^{zz}}{4}+\frac{\omega_{e1}}{2}-\frac{\omega_{e2}}{2}+\omega_n,0,0,0,0,0,0,0,0\right\},$$

$$\left\{0,0,0,0,0,0,0,0,-\frac{A^{zz}_{13}}{4}+\frac{A^{zz}_{14}}{4}+\frac{A^{zz}_{23}}{4}-\frac{A^{zz}_{24}}{4}-\frac{d^{zz}}{4}-\frac{D^{zz}}{4}-\frac{\omega_{e1}}{2}+\frac{\omega_{e2}}{2},0,0,0,0,0,0,0\right\},$$

$$\left\{0,0,0,0,0,0,0,0,0,-\frac{A^{zz}_{13}}{4}-\frac{A^{zz}_{14}}{4}+\frac{A^{zz}_{23}}{4}+\frac{A^{zz}_{24}}{4}+\frac{d^{zz}}{4}-\frac{D^{zz}}{4}-\frac{\omega_{e1}}{2}+\frac{\omega_{e2}}{2}-\omega_n,0,0,0,0,0,0\right\},$$

$$\left\{0,0,0,0,0,0,0,0,0,0,\frac{A^{zz}_{13}}{4}-\frac{A^{zz}_{14}}{4}-\frac{A^{zz}_{23}}{4}+\frac{A^{zz}_{24}}{4}-\frac{d^{zz}}{4}-\frac{D^{zz}}{4}-\frac{\omega_{e1}}{2}+\frac{\omega_{e2}}{2},0,0,0,0,0\right\},$$

$$\left\{0,0,0,0,0,0,0,0,0,0,0,\frac{A^{zz}_{13}}{4}+\frac{A^{zz}_{14}}{4}-\frac{A^{zz}_{23}}{4}-\frac{A^{zz}_{24}}{4}+\frac{d^{zz}}{4}-\frac{D^{zz}}{4}-\frac{\omega_{e1}}{2}+\frac{\omega_{e2}}{2}+\omega_n,0,0,0,0\right\},$$

$$\left\{0,0,0,0,0,0,0,0,0,0,0,0,\frac{A^{zz}_{13}}{4}+\frac{A^{zz}_{14}}{4}+\frac{A^{zz}_{23}}{4}+\frac{A^{zz}_{24}}{4}+\frac{d^{zz}}{4}+\frac{D^{zz}}{4}+\frac{\omega_{e1}}{2}+\frac{\omega_{e2}}{2}-\omega_n,0,0,0\right\},$$

$$\left\{0,0,0,0,0,0,0,0,0,0,0,0,0,-\frac{A^{zz}_{13}}{4}+\frac{A^{zz}_{14}}{4}-\frac{A^{zz}_{23}}{4}+\frac{A^{zz}_{24}}{4}-\frac{d^{zz}}{4}+\frac{D^{zz}}{4}+\frac{\omega_{e1}}{2}+\frac{\omega_{e2}}{2},0,0\right\},$$

$$\left\{0,0,0,0,0,0,0,0,0,0,0,0,0,0,\frac{A^{zz}_{13}}{4}-\frac{A^{zz}_{14}}{4}+\frac{A^{zz}_{23}}{4}-\frac{A^{zz}_{24}}{4}-\frac{d^{zz}}{4}+\frac{D^{zz}}{4}+\frac{\omega_{e1}}{2}+\frac{\omega_{e2}}{2},0\right\},$$

$$\left.\left\{0,0,0,0,0,0,0,0,0,0,0,0,0,0,0,-\frac{A^{zz}_{13}}{4}-\frac{A^{zz}_{14}}{4}-\frac{A^{zz}_{23}}{4}-\frac{A^{zz}_{24}}{4}+\frac{d^{zz}}{4}+\frac{D^{zz}}{4}+\frac{\omega_{e1}}{2}+\frac{\omega_{e2}}{2}+\omega_n\right\}\right\}$$

In[*]:=
```
SM = Table[SMe[i, k], {i, 1, 16}, {k, 1, 16}]
```
Tabelle

Out[*]=
```
{{SMe[1, 1], SMe[1, 2], SMe[1, 3], SMe[1, 4], SMe[1, 5],
  SMe[1, 6], SMe[1, 7], SMe[1, 8], SMe[1, 9], SMe[1, 10], SMe[1, 11],
  SMe[1, 12], SMe[1, 13], SMe[1, 14], SMe[1, 15], SMe[1, 16]},
 {SMe[2, 1], SMe[2, 2], SMe[2, 3], SMe[2, 4], SMe[2, 5], SMe[2, 6],
  SMe[2, 7], SMe[2, 8], SMe[2, 9], SMe[2, 10], SMe[2, 11],
  SMe[2, 12], SMe[2, 13], SMe[2, 14], SMe[2, 15], SMe[2, 16]},
 {SMe[3, 1], SMe[3, 2], SMe[3, 3], SMe[3, 4], SMe[3, 5], SMe[3, 6],
  SMe[3, 7], SMe[3, 8], SMe[3, 9], SMe[3, 10], SMe[3, 11],
  SMe[3, 12], SMe[3, 13], SMe[3, 14], SMe[3, 15], SMe[3, 16]},
 {SMe[4, 1], SMe[4, 2], SMe[4, 3], SMe[4, 4], SMe[4, 5], SMe[4, 6],
  SMe[4, 7], SMe[4, 8], SMe[4, 9], SMe[4, 10], SMe[4, 11],
  SMe[4, 12], SMe[4, 13], SMe[4, 14], SMe[4, 15], SMe[4, 16]},
 {SMe[5, 1], SMe[5, 2], SMe[5, 3], SMe[5, 4], SMe[5, 5], SMe[5, 6],
  SMe[5, 7], SMe[5, 8], SMe[5, 9], SMe[5, 10], SMe[5, 11],
  SMe[5, 12], SMe[5, 13], SMe[5, 14], SMe[5, 15], SMe[5, 16]},
 {SMe[6, 1], SMe[6, 2], SMe[6, 3], SMe[6, 4], SMe[6, 5], SMe[6, 6], SMe[6, 7],
  SMe[6, 8], SMe[6, 9], SMe[6, 10], SMe[6, 11], SMe[6, 12], SMe[6, 13],
  SMe[6, 14], SMe[6, 15], SMe[6, 16]}, {SMe[7, 1], SMe[7, 2], SMe[7, 3],
  SMe[7, 4], SMe[7, 5], SMe[7, 6], SMe[7, 7], SMe[7, 8], SMe[7, 9], SMe[7, 10],
  SMe[7, 11], SMe[7, 12], SMe[7, 13], SMe[7, 14], SMe[7, 15], SMe[7, 16]},
 {SMe[8, 1], SMe[8, 2], SMe[8, 3], SMe[8, 4], SMe[8, 5], SMe[8, 6], SMe[8, 7],
  SMe[8, 8], SMe[8, 9], SMe[8, 10], SMe[8, 11], SMe[8, 12], SMe[8, 13],
  SMe[8, 14], SMe[8, 15], SMe[8, 16]}, {SMe[9, 1], SMe[9, 2], SMe[9, 3],
  SMe[9, 4], SMe[9, 5], SMe[9, 6], SMe[9, 7], SMe[9, 8], SMe[9, 9], SMe[9, 10],
  SMe[9, 11], SMe[9, 12], SMe[9, 13], SMe[9, 14], SMe[9, 15], SMe[9, 16]},
 {SMe[10, 1], SMe[10, 2], SMe[10, 3], SMe[10, 4], SMe[10, 5], SMe[10, 6],
  SMe[10, 7], SMe[10, 8], SMe[10, 9], SMe[10, 10], SMe[10, 11],
  SMe[10, 12], SMe[10, 13], SMe[10, 14], SMe[10, 15], SMe[10, 16]},
 {SMe[11, 1], SMe[11, 2], SMe[11, 3], SMe[11, 4], SMe[11, 5], SMe[11, 6],
  SMe[11, 7], SMe[11, 8], SMe[11, 9], SMe[11, 10], SMe[11, 11],
  SMe[11, 12], SMe[11, 13], SMe[11, 14], SMe[11, 15], SMe[11, 16]},
 {SMe[12, 1], SMe[12, 2], SMe[12, 3], SMe[12, 4], SMe[12, 5], SMe[12, 6],
  SMe[12, 7], SMe[12, 8], SMe[12, 9], SMe[12, 10], SMe[12, 11],
  SMe[12, 12], SMe[12, 13], SMe[12, 14], SMe[12, 15], SMe[12, 16]},
 {SMe[13, 1], SMe[13, 2], SMe[13, 3], SMe[13, 4], SMe[13, 5], SMe[13, 6],
  SMe[13, 7], SMe[13, 8], SMe[13, 9], SMe[13, 10], SMe[13, 11],
  SMe[13, 12], SMe[13, 13], SMe[13, 14], SMe[13, 15], SMe[13, 16]},
 {SMe[14, 1], SMe[14, 2], SMe[14, 3], SMe[14, 4], SMe[14, 5], SMe[14, 6],
  SMe[14, 7], SMe[14, 8], SMe[14, 9], SMe[14, 10], SMe[14, 11],
  SMe[14, 12], SMe[14, 13], SMe[14, 14], SMe[14, 15], SMe[14, 16]},
 {SMe[15, 1], SMe[15, 2], SMe[15, 3], SMe[15, 4], SMe[15, 5], SMe[15, 6],
  SMe[15, 7], SMe[15, 8], SMe[15, 9], SMe[15, 10], SMe[15, 11],
  SMe[15, 12], SMe[15, 13], SMe[15, 14], SMe[15, 15], SMe[15, 16]},
 {SMe[16, 1], SMe[16, 2], SMe[16, 3], SMe[16, 4], SMe[16, 5], SMe[16, 6],
  SMe[16, 7], SMe[16, 8], SMe[16, 9], SMe[16, 10], SMe[16, 11],
  SMe[16, 12], SMe[16, 13], SMe[16, 14], SMe[16, 15], SMe[16, 16]}}
```

In[ ]:=
```
solution = Solve[VM + SM.H0M - H0M.SM == 0 , Flatten[SM]]
```
löse ebne ein

Solve: Equations may not give solutions for all "solve" variables.

Out[ ]=

$$\Big\{\Big\{SMe[1,2] \to -\frac{-A_{13}^{z+} - A_{23}^{z+} + d^{+z}}{A_{13}^{zz} + A_{23}^{zz} - d^{zz} + 2\,\omega_n},\; SMe[1,3] \to -\frac{-A_{14}^{z+} - A_{24}^{z+} + d^{z+}}{A_{14}^{zz} + A_{24}^{zz} - d^{zz} + 2\,\omega_n},$$

$$SMe[1,4] \to -\frac{2\,d^{++}}{A_{13}^{zz} + A_{14}^{zz} + A_{23}^{zz} + A_{24}^{zz} + 4\,\omega_n},\; SMe[1,5] \to -\frac{A_{13}^{-z} + A_{14}^{-z} - D^{-z}}{A_{13}^{zz} + A_{14}^{zz} - D^{zz} + 2\,\omega_{e1}},$$

$$SMe[1,6] \to -\frac{2\,A_{13}^{-+}}{A_{14}^{zz} + A_{23}^{zz} - d^{zz} - D^{zz} + 2\,\omega_{e1} + 2\,\omega_n},\; SMe[1,7] \to -\frac{2\,A_{14}^{-+}}{A_{13}^{zz} + A_{24}^{zz} - d^{zz} - D^{zz} + 2\,\omega_{e1} + 2\,\omega_n},$$

$$SMe[1,8] \to 0,\; SMe[1,9] \to -\frac{2\,A_{24}^{-+}}{A_{14}^{zz} + A_{23}^{zz} - d^{zz} - D^{zz} + 2\,\omega_{e2} + 2\,\omega_n},$$

$$SMe[1,10] \to -\frac{A_{23}^{-z} + A_{24}^{-z} - D^{z-}}{A_{23}^{zz} + A_{24}^{zz} - D^{zz} + 2\,\omega_{e2}},\; SMe[1,11] \to -\frac{2\,A_{23}^{-+}}{A_{13}^{zz} + A_{24}^{zz} - d^{zz} - D^{zz} + 2\,\omega_{e2} + 2\,\omega_n},$$

$$SMe[1,12] \to 0,\; SMe[1,13] \to -\frac{2\,D^{--}}{A_{13}^{zz} + A_{14}^{zz} + A_{23}^{zz} + A_{24}^{zz} + 2\,\omega_{e1} + 2\,\omega_{e2}},\; SMe[1,14] \to 0,$$

$$SMe[1,15] \to 0,\; SMe[1,16] \to 0,\; SMe[2,1] \to -\frac{A_{13}^{z-} + A_{23}^{z-} - d^{-z}}{A_{13}^{zz} + A_{23}^{zz} - d^{zz} + 2\,\omega_n},$$

$$SMe[2,3] \to \frac{2\,d^{-+}}{A_{13}^{zz} - A_{14}^{zz} + A_{23}^{zz} - A_{24}^{zz}},\; SMe[2,4] \to -\frac{-A_{14}^{z+} - A_{24}^{z+} - d^{z+}}{A_{14}^{zz} + A_{24}^{zz} + d^{zz} + 2\,\omega_n},$$

$$SMe[2,5] \to -\frac{2\,A_{13}^{--}}{A_{14}^{zz} - A_{23}^{zz} + d^{zz} - D^{zz} + 2\,\omega_{e1} - 2\,\omega_n},\; SMe[2,6] \to -\frac{A_{13}^{-z} - A_{14}^{-z} + D^{-z}}{A_{13}^{zz} - A_{14}^{zz} + D^{zz} - 2\,\omega_{e1}},$$

$$SMe[2,7] \to 0,\; SMe[2,8] \to \frac{2\,A_{14}^{-+}}{A_{13}^{zz} - A_{24}^{zz} - d^{zz} + D^{zz} - 2\,\omega_{e1} - 2\,\omega_n},$$

$$SMe[2,9] \to 0,\; SMe[2,10] \to \frac{2\,A_{23}^{--}}{A_{13}^{zz} - A_{24}^{zz} - d^{zz} + D^{zz} - 2\,\omega_{e2} + 2\,\omega_n},$$

$$SMe[2,11] \to -\frac{A_{23}^{-z} - A_{24}^{-z} + D^{z-}}{A_{23}^{zz} - A_{24}^{zz} + D^{zz} - 2\,\omega_{e2}},\; SMe[2,12] \to -\frac{2\,A_{24}^{-+}}{A_{14}^{zz} - A_{23}^{zz} + d^{zz} - D^{zz} + 2\,\omega_{e2} + 2\,\omega_n},$$

$$SMe[2,13] \to 0,\; SMe[2,14] \to \frac{2\,D^{--}}{A_{13}^{zz} - A_{14}^{zz} + A_{23}^{zz} - A_{24}^{zz} - 2\,\omega_{e1} - 2\,\omega_{e2}},$$

$$SMe[2,15] \to 0,\; SMe[2,16] \to 0,\; SMe[3,1] \to -\frac{A_{14}^{z-} + A_{24}^{z-} - d^{z-}}{A_{14}^{zz} + A_{24}^{zz} - d^{zz} + 2\,\omega_n},$$

$$SMe[3,2] \to -\frac{2\,d^{+-}}{A_{13}^{zz} - A_{14}^{zz} + A_{23}^{zz} - A_{24}^{zz}},\; SMe[3,4] \to -\frac{-A_{13}^{z+} - A_{23}^{z+} - d^{+z}}{A_{13}^{zz} + A_{23}^{zz} + d^{zz} + 2\,\omega_n},$$

$$SMe[3,5] \to -\frac{2\,A_{14}^{--}}{A_{13}^{zz} - A_{24}^{zz} + d^{zz} - D^{zz} + 2\,\omega_{e1} - 2\,\omega_n},\; SMe[3,6] \to 0,$$

$$SMe[3,7] \to -\frac{A_{13}^{-z} - A_{14}^{-z} - D^{-z}}{A_{13}^{zz} - A_{14}^{zz} - D^{zz} + 2\,\omega_{e1}},\; SMe[3,8] \to \frac{2\,A_{13}^{-+}}{A_{14}^{zz} - A_{23}^{zz} - d^{zz} + D^{zz} - 2\,\omega_{e1} - 2\,\omega_n},$$

$$SMe[3,9] \to -\frac{A_{23}^{-z} - A_{24}^{-z} - D^{z-}}{A_{23}^{zz} - A_{24}^{zz} - D^{zz} + 2\,\omega_{e2}},\; SMe[3,10] \to \frac{2\,A_{24}^{--}}{A_{14}^{zz} - A_{23}^{zz} - d^{zz} + D^{zz} - 2\,\omega_{e2} + 2\,\omega_n},$$

$$SMe[3,11] \to 0,\; SMe[3,12] \to -\frac{2\,A_{23}^{-+}}{A_{13}^{zz} - A_{24}^{zz} + d^{zz} - D^{zz} + 2\,\omega_{e2} + 2\,\omega_n},\; SMe[3,13] \to 0,$$

$$\text{SMe}[3,14]\to 0,\ \text{SMe}[3,15]\to -\frac{2\,D^{--}}{A^{zz}_{13}-A^{zz}_{14}+A^{zz}_{23}-A^{zz}_{24}+2\,\omega_{e1}+2\,\omega_{e2}},$$

$$\text{SMe}[3,16]\to 0,\ \text{SMe}[4,1]\to \frac{2\,d^{--}}{A^{zz}_{13}+A^{zz}_{14}+A^{zz}_{23}+A^{zz}_{24}+4\,\omega_{n}},$$

$$\text{SMe}[4,2]\to -\frac{A^{z-}_{14}+A^{z-}_{24}+d^{z-}}{A^{zz}_{14}+A^{zz}_{24}+d^{zz}+2\,\omega_{n}},\ \text{SMe}[4,3]\to -\frac{A^{z-}_{13}+A^{z-}_{23}+d^{-z}}{A^{zz}_{13}+A^{zz}_{23}+d^{zz}+2\,\omega_{n}},\ \text{SMe}[4,5]\to 0,$$

$$\text{SMe}[4,6]\to \frac{2\,A^{--}_{14}}{A^{zz}_{13}+A^{zz}_{24}+d^{zz}+D^{zz}-2\,\omega_{e1}+2\,\omega_{n}},\ \text{SMe}[4,7]\to \frac{2\,A^{--}_{13}}{A^{zz}_{14}+A^{zz}_{23}+d^{zz}+D^{zz}-2\,\omega_{e1}+2\,\omega_{n}},$$

$$\text{SMe}[4,8]\to -\frac{A^{-z}_{13}+A^{-z}_{14}+D^{-z}}{A^{zz}_{13}+A^{zz}_{14}+D^{zz}-2\,\omega_{e1}},\ \text{SMe}[4,9]\to \frac{2\,A^{--}_{23}}{A^{zz}_{13}+A^{zz}_{24}+d^{zz}+D^{zz}-2\,\omega_{e2}+2\,\omega_{n}},$$

$$\text{SMe}[4,10]\to 0,\ \text{SMe}[4,11]\to \frac{2\,A^{--}_{24}}{A^{zz}_{14}+A^{zz}_{23}+d^{zz}+D^{zz}-2\,\omega_{e2}+2\,\omega_{n}},$$

$$\text{SMe}[4,12]\to -\frac{A^{-z}_{23}+A^{-z}_{24}+D^{z-}}{A^{zz}_{23}+A^{zz}_{24}+D^{zz}-2\,\omega_{e2}},\ \text{SMe}[4,13]\to 0,\ \text{SMe}[4,14]\to 0,$$

$$\text{SMe}[4,15]\to 0,\ \text{SMe}[4,16]\to \frac{2\,D^{--}}{A^{zz}_{13}+A^{zz}_{14}+A^{zz}_{23}+A^{zz}_{24}-2\,\omega_{e1}-2\,\omega_{e2}},$$

$$\text{SMe}[5,1]\to -\frac{-A^{+z}_{13}-A^{+z}_{14}+D^{+z}}{A^{zz}_{13}+A^{zz}_{14}-D^{zz}+2\,\omega_{e1}},\ \text{SMe}[5,2]\to \frac{2\,A^{++}_{13}}{A^{zz}_{14}-A^{zz}_{23}+d^{zz}-D^{zz}+2\,\omega_{e1}-2\,\omega_{n}},$$

$$\text{SMe}[5,3]\to \frac{2\,A^{++}_{14}}{A^{zz}_{13}-A^{zz}_{24}+d^{zz}-D^{zz}+2\,\omega_{e1}-2\,\omega_{n}},\ \text{SMe}[5,4]\to 0,\ \text{SMe}[5,6]\to -\frac{-A^{z+}_{13}+A^{z+}_{23}-d^{+z}}{A^{zz}_{13}-A^{zz}_{23}+d^{zz}-2\,\omega_{n}},$$

$$\text{SMe}[5,7]\to -\frac{-A^{z+}_{14}+A^{z+}_{24}-d^{z+}}{A^{zz}_{14}-A^{zz}_{24}+d^{zz}-2\,\omega_{n}},\ \text{SMe}[5,8]\to \frac{2\,d^{++}}{A^{zz}_{13}+A^{zz}_{14}-A^{zz}_{23}-A^{zz}_{24}-4\,\omega_{n}},\ \text{SMe}[5,9]\to 0,$$

$$\text{SMe}[5,10]\to \frac{2\,D^{+-}}{A^{zz}_{13}+A^{zz}_{14}-A^{zz}_{23}-A^{zz}_{24}+2\,\omega_{e1}-2\,\omega_{e2}},\ \text{SMe}[5,11]\to 0,\ \text{SMe}[5,12]\to 0,$$

$$\text{SMe}[5,13]\to -\frac{A^{-z}_{23}+A^{-z}_{24}+D^{z-}}{A^{zz}_{23}+A^{zz}_{24}+D^{zz}+2\,\omega_{e2}},\ \text{SMe}[5,14]\to \frac{2\,A^{-+}_{23}}{A^{zz}_{13}-A^{zz}_{24}+d^{zz}-D^{zz}-2\,\omega_{e2}-2\,\omega_{n}},$$

$$\text{SMe}[5,15]\to \frac{2\,A^{-+}_{24}}{A^{zz}_{14}-A^{zz}_{23}+d^{zz}-D^{zz}-2\,\omega_{e2}-2\,\omega_{n}},\ \text{SMe}[5,16]\to 0,$$

$$\text{SMe}[6,1]\to \frac{2\,A^{+-}_{13}}{A^{zz}_{14}+A^{zz}_{23}-d^{zz}-D^{zz}+2\,\omega_{e1}+2\,\omega_{n}},\ \text{SMe}[6,2]\to -\frac{-A^{+z}_{13}+A^{+z}_{14}-D^{+z}}{A^{zz}_{13}-A^{zz}_{14}+D^{zz}-2\,\omega_{e1}},$$

$$\text{SMe}[6,3]\to 0,\ \text{SMe}[6,4]\to -\frac{2\,A^{++}_{14}}{A^{zz}_{13}+A^{zz}_{24}+d^{zz}+D^{zz}-2\,\omega_{e1}+2\,\omega_{n}},$$

$$\text{SMe}[6,5]\to -\frac{A^{z-}_{13}-A^{z-}_{23}+d^{-z}}{A^{zz}_{13}-A^{zz}_{23}+d^{zz}-2\,\omega_{n}},\ \text{SMe}[6,7]\to -\frac{2\,d^{-+}}{A^{zz}_{13}-A^{zz}_{14}-A^{zz}_{23}+A^{zz}_{24}},$$

$$\text{SMe}[6,8]\to -\frac{-A^{z+}_{14}+A^{z+}_{24}+d^{z+}}{A^{zz}_{14}-A^{zz}_{24}-d^{zz}-2\,\omega_{n}},\ \text{SMe}[6,9]\to 0,\ \text{SMe}[6,10]\to 0,$$

$$\text{SMe}[6,11]\to -\frac{2\,D^{+-}}{A^{zz}_{13}-A^{zz}_{14}-A^{zz}_{23}+A^{zz}_{24}-2\,\omega_{e1}+2\,\omega_{e2}},\ \text{SMe}[6,12]\to 0,$$

$$\text{SMe}[6,13]\to -\frac{2\,A^{--}_{23}}{A^{zz}_{13}+A^{zz}_{24}+d^{zz}+D^{zz}+2\,\omega_{e2}-2\,\omega_{n}},\ \text{SMe}[6,14]\to -\frac{A^{-z}_{23}-A^{-z}_{24}-D^{z-}}{A^{zz}_{23}-A^{zz}_{24}-D^{zz}-2\,\omega_{e2}},$$

$$\text{SMe}[6,15]\to 0,\ \text{SMe}[6,16]\to \frac{2\,A^{-+}_{24}}{A^{zz}_{14}+A^{zz}_{23}-d^{zz}-D^{zz}-2\,\omega_{e2}-2\,\omega_{n}},$$

$\text{SMe}[7, 1] \to \frac{2 A_{14}^{+-}}{A_{13}^{zz} + A_{24}^{zz} - d^{zz} - D^{zz} + 2\,\omega_{e1} + 2\,\omega_n}$, $\text{SMe}[7, 2] \to 0$,

$\text{SMe}[7, 3] \to -\frac{-A_{13}^{+z} + A_{14}^{+z} + D^{+z}}{A_{13}^{zz} - A_{14}^{zz} - D^{zz} + 2\,\omega_{e1}}$, $\text{SMe}[7, 4] \to -\frac{2 A_{13}^{++}}{A_{14}^{zz} + A_{23}^{zz} + d^{zz} + D^{zz} - 2\,\omega_{e1} + 2\,\omega_n}$,

$\text{SMe}[7, 5] \to -\frac{A_{14}^{z-} - A_{24}^{z-} + d^{z-}}{A_{14}^{zz} - A_{24}^{zz} + d^{zz} - 2\,\omega_n}$, $\text{SMe}[7, 6] \to \frac{2 d^{+-}}{A_{13}^{zz} - A_{14}^{zz} - A_{23}^{zz} + A_{24}^{zz}}$,

$\text{SMe}[7, 8] \to -\frac{-A_{13}^{z+} + A_{23}^{z+} + d^{+z}}{A_{13}^{zz} - A_{23}^{zz} - d^{zz} - 2\,\omega_n}$, $\text{SMe}[7, 9] \to \frac{2 D^{+-}}{A_{13}^{zz} - A_{14}^{zz} - A_{23}^{zz} + A_{24}^{zz} + 2\,\omega_{e1} - 2\,\omega_{e2}}$,

$\text{SMe}[7, 10] \to 0$, $\text{SMe}[7, 11] \to 0$, $\text{SMe}[7, 12] \to 0$,

$\text{SMe}[7, 13] \to -\frac{2 A_{24}^{--}}{A_{14}^{zz} + A_{23}^{zz} + d^{zz} + D^{zz} + 2\,\omega_{e2} - 2\,\omega_n}$, $\text{SMe}[7, 14] \to 0$,

$\text{SMe}[7, 15] \to -\frac{A_{23}^{-z} - A_{24}^{-z} + D^{z-}}{A_{23}^{zz} - A_{24}^{zz} + D^{zz} + 2\,\omega_{e2}}$, $\text{SMe}[7, 16] \to \frac{2 A_{23}^{-+}}{A_{13}^{zz} + A_{24}^{zz} - d^{zz} - D^{zz} - 2\,\omega_{e2} - 2\,\omega_n}$,

$\text{SMe}[8, 1] \to 0$, $\text{SMe}[8, 2] \to -\frac{2 A_{14}^{+-}}{A_{13}^{zz} - A_{24}^{zz} - d^{zz} + D^{zz} - 2\,\omega_{e1} - 2\,\omega_n}$,

$\text{SMe}[8, 3] \to -\frac{2 A_{13}^{+-}}{A_{14}^{zz} - A_{23}^{zz} - d^{zz} + D^{zz} - 2\,\omega_{e1} - 2\,\omega_n}$, $\text{SMe}[8, 4] \to -\frac{-A_{13}^{+z} - A_{14}^{+z} - D^{+z}}{A_{13}^{zz} + A_{14}^{zz} + D^{zz} - 2\,\omega_{e1}}$,

$\text{SMe}[8, 5] \to -\frac{2 d^{--}}{A_{13}^{zz} + A_{14}^{zz} - A_{23}^{zz} - A_{24}^{zz} - 4\,\omega_n}$, $\text{SMe}[8, 6] \to -\frac{A_{14}^{z-} - A_{24}^{z-} - d^{z-}}{A_{14}^{zz} - A_{24}^{zz} - d^{zz} - 2\,\omega_n}$,

$\text{SMe}[8, 7] \to -\frac{A_{13}^{z-} - A_{23}^{z-} - d^{-z}}{A_{13}^{zz} - A_{23}^{zz} - d^{zz} - 2\,\omega_n}$, $\text{SMe}[8, 9] \to 0$, $\text{SMe}[8, 10] \to 0$,

$\text{SMe}[8, 11] \to 0$, $\text{SMe}[8, 12] \to -\frac{2 D^{+-}}{A_{13}^{zz} + A_{14}^{zz} - A_{23}^{zz} - A_{24}^{zz} - 2\,\omega_{e1} + 2\,\omega_{e2}}$,

$\text{SMe}[8, 13] \to 0$, $\text{SMe}[8, 14] \to -\frac{2 A_{24}^{--}}{A_{14}^{zz} - A_{23}^{zz} - d^{zz} + D^{zz} + 2\,\omega_{e2} - 2\,\omega_n}$,

$\text{SMe}[8, 15] \to -\frac{2 A_{23}^{--}}{A_{13}^{zz} - A_{24}^{zz} - d^{zz} + D^{zz} + 2\,\omega_{e2} - 2\,\omega_n}$, $\text{SMe}[8, 16] \to -\frac{A_{23}^{-z} + A_{24}^{-z} - D^{z-}}{A_{23}^{zz} + A_{24}^{zz} - D^{zz} - 2\,\omega_{e2}}$,

$\text{SMe}[9, 1] \to \frac{2 A_{24}^{+-}}{A_{14}^{zz} + A_{23}^{zz} - d^{zz} - D^{zz} + 2\,\omega_{e2} + 2\,\omega_n}$, $\text{SMe}[9, 2] \to 0$,

$\text{SMe}[9, 3] \to -\frac{-A_{23}^{+z} + A_{24}^{+z} + D^{z+}}{A_{23}^{zz} - A_{24}^{zz} - D^{zz} + 2\,\omega_{e2}}$, $\text{SMe}[9, 4] \to -\frac{2 A_{23}^{++}}{A_{13}^{zz} + A_{24}^{zz} + d^{zz} + D^{zz} - 2\,\omega_{e2} + 2\,\omega_n}$,

$\text{SMe}[9, 5] \to 0$, $\text{SMe}[9, 6] \to 0$, $\text{SMe}[9, 7] \to -\frac{2 D^{-+}}{A_{13}^{zz} - A_{14}^{zz} - A_{23}^{zz} + A_{24}^{zz} + 2\,\omega_{e1} - 2\,\omega_{e2}}$,

$\text{SMe}[9, 8] \to 0$, $\text{SMe}[9, 10] \to -\frac{A_{14}^{z-} - A_{24}^{z-} - d^{z-}}{A_{14}^{zz} - A_{24}^{zz} - d^{zz} + 2\,\omega_n}$, $\text{SMe}[9, 11] \to -\frac{2 d^{+-}}{A_{13}^{zz} - A_{14}^{zz} - A_{23}^{zz} + A_{24}^{zz}}$,

$\text{SMe}[9, 12] \to -\frac{-A_{13}^{z+} + A_{23}^{z+} - d^{+z}}{A_{13}^{zz} - A_{23}^{zz} + d^{zz} + 2\,\omega_n}$, $\text{SMe}[9, 13] \to -\frac{2 A_{14}^{--}}{A_{13}^{zz} + A_{24}^{zz} + d^{zz} + D^{zz} + 2\,\omega_{e1} - 2\,\omega_n}$,

$\text{SMe}[9, 14] \to 0$, $\text{SMe}[9, 15] \to -\frac{A_{13}^{-z} - A_{14}^{-z} + D^{-z}}{A_{13}^{zz} - A_{14}^{zz} + D^{zz} + 2\,\omega_{e1}}$,

$\text{SMe}[9, 16] \to \frac{2 A_{13}^{-+}}{A_{14}^{zz} + A_{23}^{zz} - d^{zz} - D^{zz} - 2\,\omega_{e1} - 2\,\omega_n}$, $\text{SMe}[10, 1] \to -\frac{-A_{23}^{+z} - A_{24}^{+z} + D^{z+}}{A_{23}^{zz} + A_{24}^{zz} - D^{zz} + 2\,\omega_{e2}}$,

$$\text{SMe}[10,2]\to-\frac{2A_{23}^{++}}{A_{13}^{zz}-A_{24}^{zz}-d^{zz}+D^{zz}-2\,\omega_{e2}+2\,\omega_n},\ \text{SMe}[10,3]\to-\frac{2A_{24}^{++}}{A_{14}^{zz}-A_{23}^{zz}-d^{zz}+D^{zz}-2\,\omega_{e2}+2\,\omega_n},$$

$$\text{SMe}[10,4]\to0,\ \text{SMe}[10,5]\to-\frac{2D^{-+}}{A_{13}^{zz}+A_{14}^{zz}-A_{23}^{zz}-A_{24}^{zz}+2\,\omega_{e1}-2\,\omega_{e2}},\ \text{SMe}[10,6]\to0,$$

$$\text{SMe}[10,7]\to0,\ \text{SMe}[10,8]\to0,\ \text{SMe}[10,9]\to-\frac{-A_{14}^{z+}+A_{24}^{z+}+d^{z+}}{A_{14}^{zz}-A_{24}^{zz}-d^{zz}+2\,\omega_n},$$

$$\text{SMe}[10,11]\to-\frac{-A_{13}^{z+}+A_{23}^{z+}+d^{+z}}{A_{13}^{zz}-A_{23}^{zz}-d^{zz}+2\,\omega_n},\ \text{SMe}[10,12]\to-\frac{2d^{++}}{A_{13}^{zz}+A_{14}^{zz}-A_{23}^{zz}-A_{24}^{zz}+4\,\omega_n},$$

$$\text{SMe}[10,13]\to-\frac{A_{13}^{-z}+A_{14}^{-z}+D^{-z}}{A_{13}^{zz}+A_{14}^{zz}+D^{zz}+2\,\omega_{e1}},\ \text{SMe}[10,14]\to-\frac{2A_{13}^{-+}}{A_{14}^{zz}-A_{23}^{zz}-d^{zz}+D^{zz}+2\,\omega_{e1}+2\,\omega_n},$$

$$\text{SMe}[10,15]\to-\frac{2A_{14}^{-+}}{A_{13}^{zz}-A_{24}^{zz}-d^{zz}+D^{zz}+2\,\omega_{e1}+2\,\omega_n},\ \text{SMe}[10,16]\to0,$$

$$\text{SMe}[11,1]\to\frac{2A_{23}^{+-}}{A_{13}^{zz}+A_{24}^{zz}-d^{zz}-D^{zz}+2\,\omega_{e2}+2\,\omega_n},\ \text{SMe}[11,2]\to-\frac{-A_{23}^{+z}+A_{24}^{+z}-D^{z+}}{A_{23}^{zz}-A_{24}^{zz}+D^{zz}-2\,\omega_{e2}},$$

$$\text{SMe}[11,3]\to0,\ \text{SMe}[11,4]\to-\frac{2A_{24}^{++}}{A_{14}^{zz}+A_{23}^{zz}+d^{zz}+D^{zz}-2\,\omega_{e2}+2\,\omega_n},$$

$$\text{SMe}[11,5]\to0,\ \text{SMe}[11,6]\to\frac{2D^{-+}}{A_{13}^{zz}-A_{14}^{zz}-A_{23}^{zz}+A_{24}^{zz}-2\,\omega_{e1}+2\,\omega_{e2}},$$

$$\text{SMe}[11,7]\to0,\ \text{SMe}[11,8]\to0,\ \text{SMe}[11,9]\to\frac{2d^{-+}}{A_{13}^{zz}-A_{14}^{zz}-A_{23}^{zz}+A_{24}^{zz}},$$

$$\text{SMe}[11,10]\to-\frac{A_{13}^{z-}-A_{23}^{z-}-d^{-z}}{A_{13}^{zz}-A_{23}^{zz}-d^{zz}+2\,\omega_n},\ \text{SMe}[11,12]\to-\frac{-A_{14}^{z+}+A_{24}^{z+}-d^{z+}}{A_{14}^{zz}-A_{24}^{zz}+d^{zz}+2\,\omega_n},$$

$$\text{SMe}[11,13]\to-\frac{2A_{13}^{--}}{A_{14}^{zz}+A_{23}^{zz}+d^{zz}+D^{zz}+2\,\omega_{e1}-2\,\omega_n},\ \text{SMe}[11,14]\to-\frac{A_{13}^{-z}-A_{14}^{-z}-D^{-z}}{A_{13}^{zz}-A_{14}^{zz}-D^{zz}-2\,\omega_{e1}},$$

$$\text{SMe}[11,15]\to0,\ \text{SMe}[11,16]\to\frac{2A_{14}^{-+}}{A_{13}^{zz}+A_{24}^{zz}-d^{zz}-D^{zz}-2\,\omega_{e1}-2\,\omega_n},\ \text{SMe}[12,1]\to0,$$

$$\text{SMe}[12,2]\to\frac{2A_{24}^{+-}}{A_{14}^{zz}-A_{23}^{zz}+d^{zz}-D^{zz}+2\,\omega_{e2}+2\,\omega_n},\ \text{SMe}[12,3]\to\frac{2A_{23}^{+-}}{A_{13}^{zz}-A_{24}^{zz}+d^{zz}-D^{zz}+2\,\omega_{e2}+2\,\omega_n},$$

$$\text{SMe}[12,4]\to-\frac{-A_{23}^{+z}-A_{24}^{+z}-D^{z+}}{A_{23}^{zz}+A_{24}^{zz}+D^{zz}-2\,\omega_{e2}},\ \text{SMe}[12,5]\to0,\ \text{SMe}[12,6]\to0,\ \text{SMe}[12,7]\to0,$$

$$\text{SMe}[12,8]\to\frac{2D^{-+}}{A_{13}^{zz}+A_{14}^{zz}-A_{23}^{zz}-A_{24}^{zz}-2\,\omega_{e1}+2\,\omega_{e2}},\ \text{SMe}[12,9]\to-\frac{A_{13}^{z-}-A_{23}^{z-}+d^{-z}}{A_{13}^{zz}-A_{23}^{zz}+d^{zz}+2\,\omega_n},$$

$$\text{SMe}[12,10]\to\frac{2d^{--}}{A_{13}^{zz}+A_{14}^{zz}-A_{23}^{zz}-A_{24}^{zz}+4\,\omega_n},\ \text{SMe}[12,11]\to-\frac{A_{14}^{z-}-A_{24}^{z-}+d^{z-}}{A_{14}^{zz}-A_{24}^{zz}+d^{zz}+2\,\omega_n},$$

$$\text{SMe}[12,13]\to0,\ \text{SMe}[12,14]\to\frac{2A_{14}^{--}}{A_{13}^{zz}-A_{24}^{zz}+d^{zz}-D^{zz}-2\,\omega_{e1}+2\,\omega_n},$$

$$\text{SMe}[12,15]\to\frac{2A_{13}^{--}}{A_{14}^{zz}-A_{23}^{zz}+d^{zz}-D^{zz}-2\,\omega_{e1}+2\,\omega_n},\ \text{SMe}[12,16]\to-\frac{A_{13}^{-z}+A_{14}^{-z}-D^{-z}}{A_{13}^{zz}+A_{14}^{zz}-D^{zz}-2\,\omega_{e1}},$$

$$\text{SMe}[13,1]\to\frac{2D^{++}}{A_{13}^{zz}+A_{14}^{zz}+A_{23}^{zz}+A_{24}^{zz}+2\,\omega_{e1}+2\,\omega_{e2}},\ \text{SMe}[13,2]\to0,$$

$$\text{SMe}[13,3]\to0,\ \text{SMe}[13,4]\to0,\ \text{SMe}[13,5]\to-\frac{-A_{23}^{+z}-A_{24}^{+z}-D^{z+}}{A_{23}^{zz}+A_{24}^{zz}+D^{zz}+2\,\omega_{e2}},$$

$$\text{SMe}[13, 6] \to \frac{2 A_{23}^{++}}{A_{13}^{zz} + A_{24}^{zz} + d^{zz} + D^{zz} + 2 \omega_{e2} - 2 \omega_n}, \text{SMe}[13, 7] \to \frac{2 A_{24}^{++}}{A_{14}^{zz} + A_{23}^{zz} + d^{zz} + D^{zz} + 2 \omega_{e2} - 2 \omega_n},$$

$$\text{SMe}[13, 8] \to 0, \text{SMe}[13, 9] \to \frac{2 A_{14}^{++}}{A_{13}^{zz} + A_{24}^{zz} + d^{zz} + D^{zz} + 2 \omega_{e1} - 2 \omega_n},$$

$$\text{SMe}[13, 10] \to -\frac{-A_{13}^{+z} - A_{14}^{+z} - D^{+z}}{A_{13}^{zz} + A_{14}^{zz} + D^{zz} + 2 \omega_{e1}}, \text{SMe}[13, 11] \to \frac{2 A_{13}^{++}}{A_{14}^{zz} + A_{23}^{zz} + d^{zz} + D^{zz} + 2 \omega_{e1} - 2 \omega_n},$$

$$\text{SMe}[13, 12] \to 0, \text{SMe}[13, 14] \to -\frac{-A_{13}^{z+} - A_{23}^{z+} - d^{+z}}{A_{13}^{zz} + A_{23}^{zz} + d^{zz} - 2 \omega_n}, \text{SMe}[13, 15] \to -\frac{-A_{14}^{z+} - A_{24}^{z+} - d^{z+}}{A_{14}^{zz} + A_{24}^{zz} + d^{zz} - 2 \omega_n},$$

$$\text{SMe}[13, 16] \to \frac{2 d^{++}}{A_{13}^{zz} + A_{14}^{zz} + A_{23}^{zz} + A_{24}^{zz} - 4 \omega_n}, \text{SMe}[14, 1] \to 0,$$

$$\text{SMe}[14, 2] \to -\frac{2 D^{++}}{A_{13}^{zz} - A_{14}^{zz} + A_{23}^{zz} - A_{24}^{zz} - 2 \omega_{e1} - 2 \omega_{e2}}, \text{SMe}[14, 3] \to 0, \text{SMe}[14, 4] \to 0,$$

$$\text{SMe}[14, 5] \to -\frac{2 A_{23}^{+-}}{A_{13}^{zz} - A_{24}^{zz} + d^{zz} - D^{zz} - 2 \omega_{e2} - 2 \omega_n}, \text{SMe}[14, 6] \to -\frac{-A_{23}^{+z} + A_{24}^{+z} + D^{z+}}{A_{23}^{zz} - A_{24}^{zz} - D^{zz} - 2 \omega_{e2}},$$

$$\text{SMe}[14, 7] \to 0, \text{SMe}[14, 8] \to \frac{2 A_{24}^{++}}{A_{14}^{zz} - A_{23}^{zz} - d^{zz} + D^{zz} + 2 \omega_{e2} - 2 \omega_n}, \text{SMe}[14, 9] \to 0,$$

$$\text{SMe}[14, 10] \to \frac{2 A_{13}^{+-}}{A_{14}^{zz} - A_{23}^{zz} - d^{zz} + D^{zz} + 2 \omega_{e1} + 2 \omega_n}, \text{SMe}[14, 11] \to -\frac{-A_{13}^{+z} + A_{14}^{+z} + D^{+z}}{A_{13}^{zz} - A_{14}^{zz} - D^{zz} - 2 \omega_{e1}},$$

$$\text{SMe}[14, 12] \to -\frac{2 A_{14}^{++}}{A_{13}^{zz} - A_{24}^{zz} + d^{zz} - D^{zz} - 2 \omega_{e1} + 2 \omega_n}, \text{SMe}[14, 13] \to -\frac{A_{13}^{z-} + A_{23}^{z-} + d^{-z}}{A_{13}^{zz} + A_{23}^{zz} + d^{zz} - 2 \omega_n},$$

$$\text{SMe}[14, 15] \to -\frac{2 d^{-+}}{A_{13}^{zz} - A_{14}^{zz} + A_{23}^{zz} - A_{24}^{zz}}, \text{SMe}[14, 16] \to -\frac{-A_{14}^{z+} - A_{24}^{z+} + d^{z+}}{A_{14}^{zz} + A_{24}^{zz} - d^{zz} - 2 \omega_n},$$

$$\text{SMe}[15, 1] \to 0, \text{SMe}[15, 2] \to 0, \text{SMe}[15, 3] \to \frac{2 D^{++}}{A_{13}^{zz} - A_{14}^{zz} + A_{23}^{zz} - A_{24}^{zz} + 2 \omega_{e1} + 2 \omega_{e2}},$$

$$\text{SMe}[15, 4] \to 0, \text{SMe}[15, 5] \to -\frac{2 A_{24}^{+-}}{A_{14}^{zz} - A_{23}^{zz} + d^{zz} - D^{zz} - 2 \omega_{e2} - 2 \omega_n}, \text{SMe}[15, 6] \to 0,$$

$$\text{SMe}[15, 7] \to -\frac{-A_{23}^{+z} + A_{24}^{+z} - D^{z+}}{A_{23}^{zz} - A_{24}^{zz} + D^{zz} + 2 \omega_{e2}}, \text{SMe}[15, 8] \to \frac{2 A_{23}^{++}}{A_{13}^{zz} - A_{24}^{zz} - d^{zz} + D^{zz} + 2 \omega_{e2} - 2 \omega_n},$$

$$\text{SMe}[15, 9] \to -\frac{-A_{13}^{+z} + A_{14}^{+z} - D^{+z}}{A_{13}^{zz} - A_{14}^{zz} + D^{zz} + 2 \omega_{e1}}, \text{SMe}[15, 10] \to \frac{2 A_{14}^{+-}}{A_{13}^{zz} - A_{24}^{zz} - d^{zz} + D^{zz} + 2 \omega_{e1} + 2 \omega_n},$$

$$\text{SMe}[15, 11] \to 0, \text{SMe}[15, 12] \to -\frac{2 A_{13}^{++}}{A_{14}^{zz} - A_{23}^{zz} + d^{zz} - D^{zz} - 2 \omega_{e1} + 2 \omega_n},$$

$$\text{SMe}[15, 13] \to -\frac{A_{14}^{z-} + A_{24}^{z-} + d^{z-}}{A_{14}^{zz} + A_{24}^{zz} + d^{zz} - 2 \omega_n}, \text{SMe}[15, 14] \to \frac{2 d^{+-}}{A_{13}^{zz} - A_{14}^{zz} + A_{23}^{zz} - A_{24}^{zz}},$$

$$\text{SMe}[15, 16] \to -\frac{-A_{13}^{z+} - A_{23}^{z+} + d^{+z}}{A_{13}^{zz} + A_{23}^{zz} - d^{zz} - 2 \omega_n}, \text{SMe}[16, 1] \to 0, \text{SMe}[16, 2] \to 0,$$

$$\text{SMe}[16, 3] \to 0, \text{SMe}[16, 4] \to -\frac{2 D^{++}}{A_{13}^{zz} + A_{14}^{zz} + A_{23}^{zz} + A_{24}^{zz} - 2 \omega_{e1} - 2 \omega_{e2}}, \text{SMe}[16, 5] \to 0,$$

$$\text{SMe}[16, 6] \to -\frac{2 A_{24}^{+-}}{A_{14}^{zz} + A_{23}^{zz} - d^{zz} - D^{zz} - 2 \omega_{e2} - 2 \omega_n}, \text{SMe}[16, 7] \to -\frac{2 A_{23}^{+-}}{A_{13}^{zz} + A_{24}^{zz} - d^{zz} - D^{zz} - 2 \omega_{e2} - 2 \omega_n},$$

$$\text{SMe}[16, 8] \to -\frac{-A_{23}^{+z} - A_{24}^{+z} + D^{z+}}{A_{23}^{zz} + A_{24}^{zz} - D^{zz} - 2 \omega_{e2}}, \text{SMe}[16, 9] \to -\frac{2 A_{13}^{+-}}{A_{14}^{zz} + A_{23}^{zz} - d^{zz} - D^{zz} - 2 \omega_{e1} - 2 \omega_n},$$

$$\text{SMe}[16,10] \to 0,\ \text{SMe}[16,11] \to -\frac{2\,A_{14}^{+-}}{A_{13}^{zz}+A_{24}^{zz}-d^{zz}-D^{zz}-2\,\omega_{e1}-2\,\omega_n},$$

$$\text{SMe}[16,12] \to -\frac{-A_{13}^{+z}-A_{14}^{+z}+D^{+z}}{A_{13}^{zz}+A_{14}^{zz}-D^{zz}-2\,\omega_{e1}},\ \text{SMe}[16,13] \to -\frac{2\,d^{--}}{A_{13}^{zz}+A_{14}^{zz}+A_{23}^{zz}+A_{24}^{zz}-4\,\omega_n},$$

$$\text{SMe}[16,14] \to -\frac{A_{14}^{z-}+A_{24}^{z-}-d^{z-}}{A_{14}^{zz}+A_{24}^{zz}-d^{zz}-2\,\omega_n},\ \text{SMe}[16,15] \to -\frac{A_{13}^{z-}+A_{23}^{z-}-d^{-z}}{A_{13}^{zz}+A_{23}^{zz}-d^{zz}-2\,\omega_n}\}\}$$

In[∘]:=
```
SMsol = SM //. solution;
```

In[∘]:=
```
SMsol = SMsol[[1]] //. Table[SMe[i, i] → 0, {i, 1, 16}];
```
Tabelle

In[∘]:=
```
HamM2 = H0M + 1 / 2 Commutator[SMsol, VM]
```

Out[∘]=

$$\Big\{\Big\{\cdots 15\cdots + \frac{1}{2}\Bigg(-\frac{(A_{13}^{-z}+A_{14}^{-z}-D^{-z})\left(\frac{A_{13}^{+z}}{2}+\frac{A_{14}^{+z}}{2}-\frac{D^{+z}}{2}\right)}{A_{13}^{zz}+A_{14}^{zz}-D^{zz}+2\,\omega_{e1}} + \frac{\left(\frac{A_{13}^{-z}}{2}+\frac{A_{14}^{-z}}{2}-\frac{D^{-z}}{2}\right)(-A_{13}^{+z}-A_{14}^{+z}+D^{+z})}{A_{13}^{zz}+A_{14}^{zz}-D^{zz}+2\,\omega_{e1}} - \frac{(A_{23}^{-z}+A_{24}^{-z}-D^{z-})\left(\frac{A_{23}^{+z}}{2}+\frac{A_{24}^{+z}}{2}-\frac{D^{z+}}{2}\right)}{A_{23}^{zz}+A_{24}^{zz}-D^{zz}+2\,\omega_{e2}} + \frac{\left(\frac{A_{23}^{-z}}{2}+\frac{A_{24}^{-z}}{2}-\frac{D^{z-}}{2}\right)(-A_{23}^{+z}-A_{24}^{+z}+D^{z+})}{A_{23}^{zz}+A_{24}^{zz}-D^{zz}+2\,\omega_{e2}} -$$

$$\frac{4\,D^{--}\,D^{++}}{A_{13}^{zz}+A_{14}^{zz}+A_{23}^{zz}+A_{24}^{zz}+2\,\omega_{e1}+2\,\omega_{e2}} + \frac{(A_{13}^{z-}+A_{23}^{z-}-d^{-z})\left(-\frac{A_{13}^{\cdots 1\cdots}}{2}-\frac{A_{23}^{\cdots 1\cdots}}{2}+\frac{d^{\cdots 1\cdots}}{2}\right)}{A_{13}^{zz}+A_{23}^{zz}-d^{zz}+2\,\omega_n} - \frac{\cdots 1\cdots}{\cdots 1\cdots} + \cdots 1\cdots - \frac{\left(-\frac{A_{14}^{\cdots 1\cdots}}{2}-\frac{\cdots 1\cdots}{2}+\frac{\cdots 1\cdots}{2}\right)\cdots 1\cdots}{A_{14}^{zz}+A_{24}^{zz}-d^{zz}+2\,\omega_n} -$$

$$\frac{4\,A_{13}^{-+}\,A_{13}^{+-}}{A_{14}^{zz}+A_{23}^{zz}-d^{zz}-D^{zz}+2\,\omega_{e1}+2\,\omega_n} - \frac{4\,A_{14}^{-+}\,A_{14}^{+-}}{A_{13}^{zz}+A_{24}^{zz}-d^{zz}-D^{zz}+2\,\omega_{e1}+2\,\omega_n} - \frac{4\,A_{24}^{-+}\,A_{24}^{+-}}{A_{14}^{zz}+A_{23}^{zz}-d^{zz}-D^{zz}+2\,\omega_{e2}+2\,\omega_n} - \frac{4\,A_{23}^{-+}\,A_{23}^{+-}}{A_{13}^{zz}+A_{24}^{zz}-d^{zz}-D^{zz}+2\,\omega_{e2}+2\,\omega_n} - \frac{4\,d^{--}\,d^{++}}{A_{13}^{zz}+A_{14}^{zz}+A_{23}^{zz}+A_{24}^{zz}+4\,\omega_n}\Bigg),$$

$$\cdots 14\cdots,\ \frac{1}{2}(\cdots 1\cdots)\Big\},\ \cdots 14\cdots,\ \{\cdots 1\cdots\}\Big\}$$

Full expression not available (original memory size: 2.2 MB)

In[∘]:=
```
HamM2[[2, 2]]
HamM2[[3, 3]]
HamM2[[2, 2]] - HamM2[[3, 3]]
```

Out[•]=

$$\frac{A_{13}^{zz}}{4}-\frac{A_{14}^{zz}}{4}+\frac{A_{23}^{zz}}{4}-\frac{A_{24}^{zz}}{4}-\frac{d^{zz}}{4}+\frac{D^{zz}}{4}-\frac{\omega_{e1}}{2}-\frac{\omega_{e2}}{2}+$$

$$\frac{1}{2}\left(\frac{4\,d^{-+}\,d^{+-}}{A_{13}^{zz}-A_{14}^{zz}+A_{23}^{zz}-A_{24}^{zz}}+\frac{\left(-\frac{A_{13}^{-z}}{2}+\frac{A_{14}^{-z}}{2}-\frac{D^{-z}}{2}\right)\left(-A_{13}^{+z}+A_{14}^{+z}-D^{+z}\right)}{A_{13}^{zz}-A_{14}^{zz}+D^{zz}-2\,\omega_{e1}}-\right.$$

$$\frac{\left(A_{13}^{-z}-A_{14}^{-z}+D^{-z}\right)\left(-\frac{A_{13}^{+z}}{2}+\frac{A_{14}^{+z}}{2}-\frac{D^{+z}}{2}\right)}{A_{13}^{zz}-A_{14}^{zz}+D^{zz}-2\,\omega_{e1}}+\frac{\left(-\frac{A_{23}^{-z}}{2}+\frac{A_{24}^{-z}}{2}-\frac{D^{z-}}{2}\right)\left(-A_{23}^{+z}+A_{24}^{+z}-D^{z+}\right)}{A_{23}^{zz}-A_{24}^{zz}+D^{zz}-2\,\omega_{e2}}-$$

$$\frac{\left(A_{23}^{-z}-A_{24}^{-z}+D^{z-}\right)\left(-\frac{A_{23}^{+z}}{2}+\frac{A_{24}^{+z}}{2}-\frac{D^{z+}}{2}\right)}{A_{23}^{zz}-A_{24}^{zz}+D^{zz}-2\,\omega_{e2}}+\frac{4\,D^{--}\,D^{++}}{A_{13}^{zz}-A_{14}^{zz}+A_{23}^{zz}-A_{24}^{zz}-2\,\omega_{e1}-2\,\omega_{e2}}+$$

$$\frac{4\,A_{14}^{-+}\,A_{14}^{+-}}{A_{13}^{zz}-A_{24}^{zz}-d^{zz}+D^{zz}-2\,\omega_{e1}-2\,\omega_{n}}-\frac{4\,A_{13}^{--}\,A_{13}^{++}}{A_{14}^{zz}-A_{23}^{zz}+d^{zz}-D^{zz}+2\,\omega_{e1}-2\,\omega_{n}}-$$

$$\frac{\left(A_{13}^{z-}+A_{23}^{z-}-d^{-z}\right)\left(-\frac{A_{13}^{z+}}{2}-\frac{A_{23}^{z+}}{2}+\frac{d^{+z}}{2}\right)}{A_{13}^{zz}+A_{23}^{zz}-d^{zz}+2\,\omega_{n}}+\frac{\left(-\frac{A_{13}^{z-}}{2}-\frac{A_{23}^{z-}}{2}+\frac{d^{-z}}{2}\right)\left(-A_{13}^{z+}-A_{23}^{z+}+d^{+z}\right)}{A_{13}^{zz}+A_{23}^{zz}-d^{zz}+2\,\omega_{n}}-$$

$$\frac{\left(-\frac{A_{14}^{z-}}{2}-\frac{A_{24}^{z-}}{2}-\frac{d^{z-}}{2}\right)\left(-A_{14}^{z+}-A_{24}^{z+}-d^{z+}\right)}{A_{14}^{zz}+A_{24}^{zz}+d^{zz}+2\,\omega_{n}}+\frac{\left(A_{14}^{z-}+A_{24}^{z-}+d^{z-}\right)\left(-\frac{A_{14}^{z+}}{2}-\frac{A_{24}^{z+}}{2}-\frac{d^{z+}}{2}\right)}{A_{14}^{zz}+A_{24}^{zz}+d^{zz}+2\,\omega_{n}}+$$

$$\left.\frac{4\,A_{23}^{--}\,A_{23}^{++}}{A_{13}^{zz}-A_{24}^{zz}-d^{zz}+D^{zz}-2\,\omega_{e2}+2\,\omega_{n}}-\frac{4\,A_{24}^{-+}\,A_{24}^{+-}}{A_{14}^{zz}-A_{23}^{zz}+d^{zz}-D^{zz}+2\,\omega_{e2}+2\,\omega_{n}}\right)$$

Out[•]=

$$-\frac{A_{13}^{zz}}{4}+\frac{A_{14}^{zz}}{4}-\frac{A_{23}^{zz}}{4}+\frac{A_{24}^{zz}}{4}-\frac{d^{zz}}{4}+\frac{D^{zz}}{4}-\frac{\omega_{e1}}{2}-\frac{\omega_{e2}}{2}+$$

$$\frac{1}{2}\left(-\frac{4\,d^{-+}\,d^{+-}}{A_{13}^{zz}-A_{14}^{zz}+A_{23}^{zz}-A_{24}^{zz}}-\frac{\left(A_{13}^{-z}-A_{14}^{-z}-D^{-z}\right)\left(\frac{A_{13}^{+z}}{2}-\frac{A_{14}^{+z}}{2}-\frac{D^{+z}}{2}\right)}{A_{13}^{zz}-A_{14}^{zz}-D^{zz}+2\,\omega_{e1}}+\right.$$

$$\frac{\left(\frac{A_{13}^{-z}}{2}-\frac{A_{14}^{-z}}{2}-\frac{D^{-z}}{2}\right)\left(-A_{13}^{+z}+A_{14}^{+z}+D^{+z}\right)}{A_{13}^{zz}-A_{14}^{zz}-D^{zz}+2\,\omega_{e1}}-\frac{\left(A_{23}^{-z}-A_{24}^{-z}-D^{z-}\right)\left(\frac{A_{23}^{+z}}{2}-\frac{A_{24}^{+z}}{2}-\frac{D^{z+}}{2}\right)}{A_{23}^{zz}-A_{24}^{zz}-D^{zz}+2\,\omega_{e2}}+$$

$$\frac{\left(\frac{A_{23}^{-z}}{2}-\frac{A_{24}^{-z}}{2}-\frac{D^{z-}}{2}\right)\left(-A_{23}^{+z}+A_{24}^{+z}+D^{z+}\right)}{A_{23}^{zz}-A_{24}^{zz}-D^{zz}+2\,\omega_{e2}}-\frac{4\,D^{--}\,D^{++}}{A_{13}^{zz}-A_{14}^{zz}+A_{23}^{zz}-A_{24}^{zz}+2\,\omega_{e1}+2\,\omega_{e2}}+$$

$$\frac{4\,A_{13}^{-+}\,A_{13}^{+-}}{A_{14}^{zz}-A_{23}^{zz}-d^{zz}+D^{zz}-2\,\omega_{e1}-2\,\omega_{n}}-\frac{4\,A_{14}^{--}\,A_{14}^{++}}{A_{13}^{zz}-A_{24}^{zz}+d^{zz}-D^{zz}+2\,\omega_{e1}-2\,\omega_{n}}-$$

$$\frac{\left(A_{14}^{z-}+A_{24}^{z-}-d^{z-}\right)\left(-\frac{A_{14}^{z+}}{2}-\frac{A_{24}^{z+}}{2}+\frac{d^{z+}}{2}\right)}{A_{14}^{zz}+A_{24}^{zz}-d^{zz}+2\,\omega_{n}}+\frac{\left(-\frac{A_{14}^{z-}}{2}-\frac{A_{24}^{z-}}{2}+\frac{d^{z-}}{2}\right)\left(-A_{14}^{z+}-A_{24}^{z+}+d^{z+}\right)}{A_{14}^{zz}+A_{24}^{zz}-d^{zz}+2\,\omega_{n}}-$$

$$\frac{\left(-\frac{A_{13}^{z-}}{2}-\frac{A_{23}^{z-}}{2}-\frac{d^{-z}}{2}\right)\left(-A_{13}^{z+}-A_{23}^{z+}-d^{+z}\right)}{A_{13}^{zz}+A_{23}^{zz}+d^{zz}+2\,\omega_{n}}+\frac{\left(A_{13}^{z-}+A_{23}^{z-}+d^{-z}\right)\left(-\frac{A_{13}^{z+}}{2}-\frac{A_{23}^{z+}}{2}-\frac{d^{+z}}{2}\right)}{A_{13}^{zz}+A_{23}^{zz}+d^{zz}+2\,\omega_{n}}+$$

$$\left.\frac{4\,A_{24}^{--}\,A_{24}^{++}}{A_{14}^{zz}-A_{23}^{zz}-d^{zz}+D^{zz}-2\,\omega_{e2}+2\,\omega_{n}}-\frac{4\,A_{23}^{-+}\,A_{23}^{+-}}{A_{13}^{zz}-A_{24}^{zz}+d^{zz}-D^{zz}+2\,\omega_{e2}+2\,\omega_{n}}\right)$$

*Out[ ]=*

$$\frac{A_{13}^{zz}}{2}-\frac{A_{14}^{zz}}{2}+\frac{A_{23}^{zz}}{2}-\frac{A_{24}^{zz}}{2}+$$

$$\frac{1}{2}\Bigg(\frac{4\,d^{-+}\,d^{+-}}{A_{13}^{zz}-A_{14}^{zz}+A_{23}^{zz}-A_{24}^{zz}}+\frac{\left(-\frac{A_{13}^{-z}}{2}+\frac{A_{14}^{-z}}{2}-\frac{D^{-z}}{2}\right)\left(-A_{13}^{+z}+A_{14}^{+z}-D^{+z}\right)}{A_{13}^{zz}-A_{14}^{zz}+D^{zz}-2\,\omega_{e1}}-\frac{\left(A_{13}^{-z}-A_{14}^{-z}+D^{-z}\right)\left(-\frac{A_{13}^{+z}}{2}+\frac{A_{14}^{+z}}{2}-\frac{D^{+z}}{2}\right)}{A_{13}^{zz}-A_{14}^{zz}+D^{zz}-2\,\omega_{e1}}+$$

$$\frac{\left(-\frac{A_{23}^{-z}}{2}+\frac{A_{24}^{-z}}{2}-\frac{D^{z-}}{2}\right)\left(-A_{23}^{+z}+A_{24}^{+z}-D^{z+}\right)}{A_{23}^{zz}-A_{24}^{zz}+D^{zz}-2\,\omega_{e2}}-\frac{\left(A_{23}^{-z}-A_{24}^{-z}+D^{z-}\right)\left(-\frac{A_{23}^{+z}}{2}+\frac{A_{24}^{+z}}{2}-\frac{D^{z+}}{2}\right)}{A_{23}^{zz}-A_{24}^{zz}+D^{zz}-2\,\omega_{e2}}+$$

$$\frac{4\,D^{--}\,D^{++}}{A_{13}^{zz}-A_{14}^{zz}+A_{23}^{zz}-A_{24}^{zz}-2\,\omega_{e1}-2\,\omega_{e2}}+\frac{4\,A_{14}^{-+}\,A_{14}^{+-}}{A_{13}^{zz}-A_{24}^{zz}-d^{zz}+D^{zz}-2\,\omega_{e1}-2\,\omega_{n}}-$$

$$\frac{4\,A_{13}^{--}\,A_{13}^{++}}{A_{14}^{zz}-A_{23}^{zz}+d^{zz}-D^{zz}+2\,\omega_{e1}-2\,\omega_{n}}-\frac{\left(A_{13}^{z-}+A_{23}^{z-}-d^{-z}\right)\left(-\frac{A_{13}^{z+}}{2}-\frac{A_{23}^{z+}}{2}+\frac{d^{+z}}{2}\right)}{A_{13}^{zz}+A_{23}^{zz}-d^{zz}+2\,\omega_{n}}+$$

$$\frac{\left(-\frac{A_{13}^{z-}}{2}-\frac{A_{23}^{z-}}{2}+\frac{d^{-z}}{2}\right)\left(-A_{13}^{z+}-A_{23}^{z+}+d^{+z}\right)}{A_{13}^{zz}+A_{23}^{zz}-d^{zz}+2\,\omega_{n}}-$$

$$\frac{\left(-\frac{A_{14}^{z-}}{2}-\frac{A_{24}^{z-}}{2}-\frac{d^{z-}}{2}\right)\left(-A_{14}^{z+}-A_{24}^{z+}-d^{z+}\right)}{A_{14}^{zz}+A_{24}^{zz}+d^{zz}+2\,\omega_{n}}+\frac{\left(A_{14}^{z-}+A_{24}^{z-}+d^{z-}\right)\left(-\frac{A_{14}^{z+}}{2}-\frac{A_{24}^{z+}}{2}-\frac{d^{z+}}{2}\right)}{A_{14}^{zz}+A_{24}^{zz}+d^{zz}+2\,\omega_{n}}+$$

$$\frac{4\,A_{23}^{--}\,A_{23}^{++}}{A_{13}^{zz}-A_{24}^{zz}-d^{zz}+D^{zz}-2\,\omega_{e2}+2\,\omega_{n}}-\frac{4\,A_{24}^{-+}\,A_{24}^{+-}}{A_{14}^{zz}-A_{23}^{zz}+d^{zz}-D^{zz}+2\,\omega_{e2}+2\,\omega_{n}}\Bigg)+$$

$$\frac{1}{2}\Bigg(\frac{4\,d^{-+}\,d^{+-}}{A_{13}^{zz}-A_{14}^{zz}+A_{23}^{zz}-A_{24}^{zz}}+\frac{\left(A_{13}^{-z}-A_{14}^{-z}-D^{-z}\right)\left(\frac{A_{13}^{+z}}{2}-\frac{A_{14}^{+z}}{2}-\frac{D^{+z}}{2}\right)}{A_{13}^{zz}-A_{14}^{zz}-D^{zz}+2\,\omega_{e1}}-$$

$$\frac{\left(\frac{A_{13}^{-z}}{2}-\frac{A_{14}^{-z}}{2}-\frac{D^{-z}}{2}\right)\left(-A_{13}^{+z}+A_{14}^{+z}+D^{+z}\right)}{A_{13}^{zz}-A_{14}^{zz}-D^{zz}+2\,\omega_{e1}}+\frac{\left(A_{23}^{-z}-A_{24}^{-z}-D^{z-}\right)\left(\frac{A_{23}^{+z}}{2}-\frac{A_{24}^{+z}}{2}-\frac{D^{z+}}{2}\right)}{A_{23}^{zz}-A_{24}^{zz}-D^{zz}+2\,\omega_{e2}}-$$

$$\frac{\left(\frac{A_{23}^{-z}}{2}-\frac{A_{24}^{-z}}{2}-\frac{D^{z-}}{2}\right)\left(-A_{23}^{+z}+A_{24}^{+z}+D^{z+}\right)}{A_{23}^{zz}-A_{24}^{zz}-D^{zz}+2\,\omega_{e2}}+\frac{4\,D^{--}\,D^{++}}{A_{13}^{zz}-A_{14}^{zz}+A_{23}^{zz}-A_{24}^{zz}+2\,\omega_{e1}+2\,\omega_{e2}}-$$

$$\frac{4\,A_{13}^{-+}\,A_{13}^{+-}}{A_{14}^{zz}-A_{23}^{zz}-d^{zz}+D^{zz}-2\,\omega_{e1}-2\,\omega_{n}}+\frac{4\,A_{14}^{--}\,A_{14}^{++}}{A_{13}^{zz}-A_{24}^{zz}+d^{zz}-D^{zz}+2\,\omega_{e1}-2\,\omega_{n}}+$$

$$\frac{\left(A_{14}^{z-}+A_{24}^{z-}-d^{z-}\right)\left(-\frac{A_{14}^{z+}}{2}-\frac{A_{24}^{z+}}{2}+\frac{d^{z+}}{2}\right)}{A_{14}^{zz}+A_{24}^{zz}-d^{zz}+2\,\omega_{n}}-\frac{\left(-\frac{A_{14}^{z-}}{2}-\frac{A_{24}^{z-}}{2}+\frac{d^{z-}}{2}\right)\left(-A_{14}^{z+}-A_{24}^{z+}+d^{z+}\right)}{A_{14}^{zz}+A_{24}^{zz}-d^{zz}+2\,\omega_{n}}+$$

$$\frac{\left(-\frac{A_{13}^{z-}}{2}-\frac{A_{23}^{z-}}{2}-\frac{d^{-z}}{2}\right)\left(-A_{13}^{z+}-A_{23}^{z+}-d^{+z}\right)}{A_{13}^{zz}+A_{23}^{zz}+d^{zz}+2\,\omega_{n}}-\frac{\left(A_{13}^{z-}+A_{23}^{z-}+d^{-z}\right)\left(-\frac{A_{13}^{z+}}{2}-\frac{A_{23}^{z+}}{2}-\frac{d^{+z}}{2}\right)}{A_{13}^{zz}+A_{23}^{zz}+d^{zz}+2\,\omega_{n}}-$$

$$\frac{4\,A_{24}^{--}\,A_{24}^{++}}{A_{14}^{zz}-A_{23}^{zz}-d^{zz}+D^{zz}-2\,\omega_{e2}+2\,\omega_{n}}+\frac{4\,A_{23}^{-+}\,A_{23}^{+-}}{A_{13}^{zz}-A_{24}^{zz}+d^{zz}-D^{zz}+2\,\omega_{e2}+2\,\omega_{n}}\Bigg)$$

In[∘]:= **HamM2⟦3, 2⟧**

Out[∘]=

$$\frac{1}{2}\left(\frac{2 A_{13}^{-+} A_{14}^{+-}}{A_{14}^{zz} - A_{23}^{zz} - d^{zz} + D^{zz} - 2\,\omega_{e1} - 2\,\omega_n} + \frac{2 A_{13}^{-+} A_{14}^{+-}}{A_{13}^{zz} - A_{24}^{zz} - d^{zz} + D^{zz} - 2\,\omega_{e1} - 2\,\omega_n} - \right.$$

$$\frac{2 A_{13}^{++} A_{14}^{--}}{A_{14}^{zz} - A_{23}^{zz} + d^{zz} - D^{zz} + 2\,\omega_{e1} - 2\,\omega_n} - \frac{2 A_{13}^{++} A_{14}^{--}}{A_{13}^{zz} - A_{24}^{zz} + d^{zz} - D^{zz} + 2\,\omega_{e1} - 2\,\omega_n} +$$

$$\frac{\left(-A_{13}^{z+} - A_{23}^{z+} + d^{+z}\right)\left(-\frac{A_{14}^{z-}}{2} - \frac{A_{24}^{z-}}{2} + \frac{d^{z-}}{2}\right)}{A_{13}^{zz} + A_{23}^{zz} - d^{zz} + 2\,\omega_n} - \frac{\left(-\frac{A_{13}^{z+}}{2} - \frac{A_{23}^{z+}}{2} + \frac{d^{+z}}{2}\right)\left(A_{14}^{z-} + A_{24}^{z-} - d^{z-}\right)}{A_{14}^{zz} + A_{24}^{zz} - d^{zz} + 2\,\omega_n} -$$

$$\frac{\left(-A_{13}^{z+} - A_{23}^{z+} - d^{+z}\right)\left(-\frac{A_{14}^{z-}}{2} - \frac{A_{24}^{z-}}{2} - \frac{d^{z-}}{2}\right)}{A_{13}^{zz} + A_{23}^{zz} + d^{zz} + 2\,\omega_n} + \frac{\left(-\frac{A_{13}^{z+}}{2} - \frac{A_{23}^{z+}}{2} - \frac{d^{+z}}{2}\right)\left(A_{14}^{z-} + A_{24}^{z-} + d^{z-}\right)}{A_{14}^{zz} + A_{24}^{zz} + d^{zz} + 2\,\omega_n} +$$

$$\frac{2 A_{23}^{++} A_{24}^{--}}{A_{14}^{zz} - A_{23}^{zz} - d^{zz} + D^{zz} - 2\,\omega_{e2} + 2\,\omega_n} + \frac{2 A_{23}^{++} A_{24}^{--}}{A_{13}^{zz} - A_{24}^{zz} - d^{zz} + D^{zz} - 2\,\omega_{e2} + 2\,\omega_n} -$$

$$\left.\frac{2 A_{23}^{-+} A_{24}^{+-}}{A_{14}^{zz} - A_{23}^{zz} + d^{zz} - D^{zz} + 2\,\omega_{e2} + 2\,\omega_n} - \frac{2 A_{23}^{-+} A_{24}^{+-}}{A_{13}^{zz} - A_{24}^{zz} + d^{zz} - D^{zz} + 2\,\omega_{e2} + 2\,\omega_n}\right)$$

In[ ]:= **HamM2⟦6, 6⟧ − HamM2⟦9, 9⟧**

Out[ ]=

$$\omega_{e1} - \omega_{e2} +$$

$$\frac{1}{2}\Bigg( -\frac{4\, d^{-+}\, d^{+-}}{A^{zz}_{13} - A^{zz}_{14} - A^{zz}_{23} + A^{zz}_{24}} - \frac{\left(-\frac{A^{-z}_{13}}{2} + \frac{A^{-z}_{14}}{2} - \frac{D^{-z}}{2}\right)\left(-A^{+z}_{13} + A^{+z}_{14} - D^{+z}\right)}{A^{zz}_{13} - A^{zz}_{14} + D^{zz} - 2\,\omega_{e1}} + \frac{\left(A^{-z}_{13} - A^{-z}_{14} + D^{-z}\right)\left(-\frac{A^{+z}_{13}}{2} + \frac{A^{+z}_{14}}{2} - \frac{D^{+z}}{2}\right)}{A^{zz}_{13} - A^{zz}_{14} + D^{zz} - 2\,\omega_{e1}} -$$

$$\frac{\left(A^{-z}_{23} - A^{-z}_{24} - D^{z-}\right)\left(-\frac{A^{+z}_{23}}{2} + \frac{A^{+z}_{24}}{2} + \frac{D^{z+}}{2}\right)}{A^{zz}_{23} - A^{zz}_{24} - D^{zz} - 2\,\omega_{e2}} + \frac{\left(-\frac{A^{-z}_{23}}{2} + \frac{A^{-z}_{24}}{2} + \frac{D^{z-}}{2}\right)\left(-A^{+z}_{23} + A^{+z}_{24} + D^{z+}\right)}{A^{zz}_{23} - A^{zz}_{24} - D^{zz} - 2\,\omega_{e2}} -$$

$$\frac{4\, D^{-+}\, D^{+-}}{A^{zz}_{13} - A^{zz}_{14} - A^{zz}_{23} + A^{zz}_{24} - 2\,\omega_{e1} + 2\,\omega_{e2}} + \frac{\left(A^{z-}_{14} - A^{z-}_{24} - d^{z-}\right)\left(\frac{A^{z+}_{14}}{2} - \frac{A^{z+}_{24}}{2} - \frac{d^{z+}}{2}\right)}{A^{zz}_{14} - A^{zz}_{24} - d^{zz} - 2\,\omega_{n}} -$$

$$\frac{\left(\frac{A^{z-}_{14}}{2} - \frac{A^{z-}_{24}}{2} - \frac{d^{z-}}{2}\right)\left(-A^{z+}_{14} + A^{z+}_{24} + d^{z+}\right)}{A^{zz}_{14} - A^{zz}_{24} - d^{zz} - 2\,\omega_{n}} +$$

$$\frac{\left(\frac{A^{z-}_{13}}{2} - \frac{A^{z-}_{23}}{2} + \frac{d^{-z}}{2}\right)\left(-A^{z+}_{13} + A^{z+}_{23} - d^{+z}\right)}{A^{zz}_{13} - A^{zz}_{23} + d^{zz} - 2\,\omega_{n}} - \frac{\left(A^{z-}_{13} - A^{z-}_{23} + d^{-z}\right)\left(\frac{A^{z+}_{13}}{2} - \frac{A^{z+}_{23}}{2} + \frac{d^{+z}}{2}\right)}{A^{zz}_{13} - A^{zz}_{23} + d^{zz} - 2\,\omega_{n}} +$$

$$\frac{4\, A^{-+}_{24}\, A^{+-}_{24}}{A^{zz}_{14} + A^{zz}_{23} - d^{zz} - D^{zz} - 2\,\omega_{e2} - 2\,\omega_{n}} - \frac{4\, A^{--}_{23}\, A^{++}_{23}}{A^{zz}_{13} + A^{zz}_{24} + d^{zz} + D^{zz} + 2\,\omega_{e2} - 2\,\omega_{n}} -$$

$$\frac{4\, A^{--}_{14}\, A^{++}_{14}}{A^{zz}_{13} + A^{zz}_{24} + d^{zz} + D^{zz} - 2\,\omega_{e1} + 2\,\omega_{n}} + \frac{4\, A^{-+}_{13}\, A^{+-}_{13}}{A^{zz}_{14} + A^{zz}_{23} - d^{zz} - D^{zz} + 2\,\omega_{e1} + 2\,\omega_{n}}\Bigg) +$$

$$\frac{1}{2}\Bigg( \frac{4\, d^{-+}\, d^{+-}}{A^{zz}_{13} - A^{zz}_{14} - A^{zz}_{23} + A^{zz}_{24}} - \frac{\left(\frac{A^{-z}_{13}}{2} - \frac{A^{-z}_{14}}{2} + \frac{D^{-z}}{2}\right)\left(-A^{+z}_{13} + A^{+z}_{14} - D^{+z}\right)}{A^{zz}_{13} - A^{zz}_{14} + D^{zz} + 2\,\omega_{e1}} +$$

$$\frac{\left(A^{-z}_{13} - A^{-z}_{14} + D^{-z}\right)\left(\frac{A^{+z}_{13}}{2} - \frac{A^{+z}_{14}}{2} + \frac{D^{+z}}{2}\right)}{A^{zz}_{13} - A^{zz}_{14} + D^{zz} + 2\,\omega_{e1}} + \frac{4\, D^{-+}\, D^{+-}}{A^{zz}_{13} - A^{zz}_{14} - A^{zz}_{23} + A^{zz}_{24} + 2\,\omega_{e1} - 2\,\omega_{e2}} -$$

$$\frac{\left(A^{-z}_{23} - A^{-z}_{24} - D^{z-}\right)\left(\frac{A^{+z}_{23}}{2} - \frac{A^{+z}_{24}}{2} - \frac{D^{z+}}{2}\right)}{A^{zz}_{23} - A^{zz}_{24} - D^{zz} + 2\,\omega_{e2}} + \frac{\left(\frac{A^{-z}_{23}}{2} - \frac{A^{-z}_{24}}{2} - \frac{D^{z-}}{2}\right)\left(-A^{+z}_{23} + A^{+z}_{24} + D^{z+}\right)}{A^{zz}_{23} - A^{zz}_{24} - D^{zz} + 2\,\omega_{e2}} -$$

$$\frac{4\, A^{-+}_{13}\, A^{+-}_{13}}{A^{zz}_{14} + A^{zz}_{23} - d^{zz} - D^{zz} - 2\,\omega_{e1} - 2\,\omega_{n}} + \frac{4\, A^{--}_{14}\, A^{++}_{14}}{A^{zz}_{13} + A^{zz}_{24} + d^{zz} + D^{zz} + 2\,\omega_{e1} - 2\,\omega_{n}} +$$

$$\frac{\left(A^{z-}_{14} - A^{z-}_{24} - d^{z-}\right)\left(-\frac{A^{z+}_{14}}{2} + \frac{A^{z+}_{24}}{2} + \frac{d^{z+}}{2}\right)}{A^{zz}_{14} - A^{zz}_{24} - d^{zz} + 2\,\omega_{n}} - \frac{\left(-\frac{A^{z-}_{14}}{2} + \frac{A^{z-}_{24}}{2} + \frac{d^{z-}}{2}\right)\left(-A^{z+}_{14} + A^{z+}_{24} + d^{z+}\right)}{A^{zz}_{14} - A^{zz}_{24} - d^{zz} + 2\,\omega_{n}} +$$

$$\frac{\left(-\frac{A^{z-}_{13}}{2} + \frac{A^{z-}_{23}}{2} - \frac{d^{-z}}{2}\right)\left(-A^{z+}_{13} + A^{z+}_{23} - d^{+z}\right)}{A^{zz}_{13} - A^{zz}_{23} + d^{zz} + 2\,\omega_{n}} - \frac{\left(A^{z-}_{13} - A^{z-}_{23} + d^{-z}\right)\left(-\frac{A^{z+}_{13}}{2} + \frac{A^{z+}_{23}}{2} - \frac{d^{+z}}{2}\right)}{A^{zz}_{13} - A^{zz}_{23} + d^{zz} + 2\,\omega_{n}} +$$

$$\frac{4\, A^{--}_{23}\, A^{++}_{23}}{A^{zz}_{13} + A^{zz}_{24} + d^{zz} + D^{zz} - 2\,\omega_{e2} + 2\,\omega_{n}} - \frac{4\, A^{-+}_{24}\, A^{+-}_{24}}{A^{zz}_{14} + A^{zz}_{23} - d^{zz} - D^{zz} + 2\,\omega_{e2} + 2\,\omega_{n}}\Bigg)$$

In[]:= **HamM2[[5, 3]]**

Out[]=

$$\frac{1}{2}\left(-\frac{2 A_{13}^{++} d^{-+}}{A_{13}^{zz}-A_{14}^{zz}+A_{23}^{zz}-A_{24}^{zz}}+\frac{\left(-A_{13}^{+z}+A_{14}^{+z}+D^{+z}\right)\left(\frac{A_{14}^{z+}}{2}-\frac{A_{24}^{z+}}{2}+\frac{d^{z+}}{2}\right)}{A_{13}^{zz}-A_{14}^{zz}-D^{zz}+2\,\omega_{e1}}-\frac{\left(-A_{13}^{+z}-A_{14}^{+z}+D^{+z}\right)\left(-\frac{A_{14}^{z+}}{2}-\frac{A_{24}^{z+}}{2}+\frac{d^{z+}}{2}\right)}{A_{13}^{zz}+A_{14}^{zz}-D^{zz}+2\,\omega_{e1}}+\right.$$
$$\frac{2 A_{24}^{++} D^{+-}}{A_{13}^{zz}+A_{14}^{zz}-A_{23}^{zz}-A_{24}^{zz}+2\,\omega_{e1}-2\,\omega_{e2}}-\frac{2 A_{24}^{-+} D^{++}}{A_{13}^{zz}-A_{14}^{zz}+A_{23}^{zz}-A_{24}^{zz}+2\,\omega_{e1}+2\,\omega_{e2}}+\frac{2 A_{13}^{+-} d^{++}}{A_{13}^{zz}+A_{14}^{zz}-A_{23}^{zz}-A_{24}^{zz}-4\,\omega_n}-$$
$$\frac{\left(\frac{A_{13}^{+z}}{2}-\frac{A_{14}^{+z}}{2}-\frac{D^{+z}}{2}\right)\left(-A_{14}^{z+}+A_{24}^{z+}-d^{z+}\right)}{A_{14}^{zz}-A_{24}^{zz}+d^{zz}-2\,\omega_n}+\frac{2 A_{13}^{+-} d^{++}}{A_{14}^{zz}-A_{23}^{zz}-d^{zz}+D^{zz}-2\,\omega_{e1}-2\,\omega_n}+$$
$$\frac{2 A_{13}^{++} d^{-+}}{A_{14}^{zz}-A_{23}^{zz}+d^{zz}-D^{zz}+2\,\omega_{e1}-2\,\omega_n}+\frac{2 A_{24}^{-+} D^{++}}{A_{14}^{zz}-A_{23}^{zz}+d^{zz}-D^{zz}-2\,\omega_{e2}-2\,\omega_n}+$$
$$\left.\frac{\left(\frac{A_{13}^{+z}}{2}+\frac{A_{14}^{+z}}{2}-\frac{D^{+z}}{2}\right)\left(-A_{14}^{z+}-A_{24}^{z+}+d^{z+}\right)}{A_{14}^{zz}+A_{24}^{zz}-d^{zz}+2\,\omega_n}+\frac{2 A_{24}^{++} D^{+-}}{A_{14}^{zz}-A_{23}^{zz}-d^{zz}+D^{zz}-2\,\omega_{e2}+2\,\omega_n}\right)$$

In[]:= **HamM2[[7, 2]]**

Out[]=

$$\frac{1}{2}\left(\frac{2 d^{+-}\left(\frac{A_{13}^{+z}}{2}-\frac{A_{14}^{+z}}{2}-\frac{D^{+z}}{2}\right)}{A_{13}^{zz}-A_{14}^{zz}+A_{23}^{zz}-A_{24}^{zz}}+\frac{2 d^{+-}\left(-\frac{A_{13}^{+z}}{2}+\frac{A_{14}^{+z}}{2}-\frac{D^{+z}}{2}\right)}{A_{13}^{zz}-A_{14}^{zz}-A_{23}^{zz}+A_{24}^{zz}}+\frac{d^{+-}\left(-A_{13}^{+z}+A_{14}^{+z}-D^{+z}\right)}{A_{13}^{zz}-A_{14}^{zz}+D^{zz}-2\,\omega_{e1}}-\frac{d^{+-}\left(-A_{13}^{+z}+A_{14}^{+z}+D^{+z}\right)}{A_{13}^{zz}-A_{14}^{zz}-D^{zz}+2\,\omega_{e1}}-\right.$$
$$\frac{A_{14}^{+-}\left(-A_{13}^{z+}+A_{23}^{z+}+d^{+z}\right)}{A_{13}^{zz}-A_{23}^{zz}-d^{zz}-2\,\omega_n}-\frac{A_{13}^{++}\left(A_{14}^{z-}-A_{24}^{z-}+d^{z-}\right)}{A_{14}^{zz}-A_{24}^{zz}+d^{zz}-2\,\omega_n}+\frac{2 A_{14}^{+-}\left(\frac{A_{13}^{z+}}{2}-\frac{A_{23}^{z+}}{2}-\frac{d^{+z}}{2}\right)}{A_{13}^{zz}-A_{24}^{zz}-d^{zz}+D^{zz}-2\,\omega_{e1}-2\,\omega_n}-$$
$$\frac{2 A_{13}^{++}\left(\frac{A_{14}^{z-}}{2}-\frac{A_{24}^{z-}}{2}+\frac{d^{z-}}{2}\right)}{A_{14}^{zz}-A_{23}^{zz}+d^{zz}-D^{zz}+2\,\omega_{e1}-2\,\omega_n}+\frac{A_{14}^{+-}\left(-A_{13}^{z+}-A_{23}^{z+}+d^{+z}\right)}{A_{13}^{zz}+A_{23}^{zz}-d^{zz}+2\,\omega_n}+\frac{A_{13}^{++}\left(A_{14}^{z-}+A_{24}^{z-}+d^{z-}\right)}{A_{14}^{zz}+A_{24}^{zz}+d^{zz}+2\,\omega_n}-$$
$$\left.\frac{2 A_{13}^{++}\left(-\frac{A_{14}^{z-}}{2}-\frac{A_{24}^{z-}}{2}-\frac{d^{z-}}{2}\right)}{A_{14}^{zz}+A_{23}^{zz}+d^{zz}+D^{zz}-2\,\omega_{e1}+2\,\omega_n}+\frac{2 A_{14}^{+-}\left(-\frac{A_{13}^{z+}}{2}-\frac{A_{23}^{z+}}{2}+\frac{d^{+z}}{2}\right)}{A_{13}^{zz}+A_{24}^{zz}-d^{zz}-D^{zz}+2\,\omega_{e1}+2\,\omega_n}\right)$$

In[]:= **HamM2[[6, 9]]**

Out[]=

$$\frac{1}{2}\left(-\frac{4 d^{-+} D^{+-}}{A_{13}^{zz}-A_{14}^{zz}-A_{23}^{zz}+A_{24}^{zz}}-\frac{2 d^{-+} D^{+-}}{A_{13}^{zz}-A_{14}^{zz}-A_{23}^{zz}+A_{24}^{zz}+2\,\omega_{e1}-2\,\omega_{e2}}-\frac{2 d^{-+} D^{+-}}{A_{13}^{zz}-A_{14}^{zz}-A_{23}^{zz}+A_{24}^{zz}-2\,\omega_{e1}+2\,\omega_{e2}}+\right.$$
$$\frac{2 A_{13}^{+-} A_{24}^{-+}}{A_{14}^{zz}+A_{23}^{zz}-d^{zz}-D^{zz}-2\,\omega_{e1}-2\,\omega_n}-\frac{2 A_{14}^{++} A_{23}^{--}}{A_{13}^{zz}+A_{24}^{zz}+d^{zz}+D^{zz}+2\,\omega_{e1}-2\,\omega_n}+$$
$$\frac{2 A_{13}^{+-} A_{24}^{-+}}{A_{14}^{zz}+A_{23}^{zz}-d^{zz}-D^{zz}-2\,\omega_{e2}-2\,\omega_n}-\frac{2 A_{14}^{++} A_{23}^{--}}{A_{13}^{zz}+A_{24}^{zz}+d^{zz}+D^{zz}+2\,\omega_{e2}-2\,\omega_n}-$$
$$\frac{2 A_{14}^{++} A_{23}^{--}}{A_{13}^{zz}+A_{24}^{zz}+d^{zz}+D^{zz}-2\,\omega_{e1}+2\,\omega_n}+\frac{2 A_{13}^{+-} A_{24}^{-+}}{A_{14}^{zz}+A_{23}^{zz}-d^{zz}-D^{zz}+2\,\omega_{e1}+2\,\omega_n}-$$
$$\left.\frac{2 A_{14}^{++} A_{23}^{--}}{A_{13}^{zz}+A_{24}^{zz}+d^{zz}+D^{zz}-2\,\omega_{e2}+2\,\omega_n}+\frac{2 A_{13}^{+-} A_{24}^{-+}}{A_{14}^{zz}+A_{23}^{zz}-d^{zz}-D^{zz}+2\,\omega_{e2}+2\,\omega_n}\right)$$

In[∘]:= **HamM2⟦6, 10⟧**

Out[∘]=

$$\frac{1}{2}\left(-\frac{A_{23}^{--}\left(-A_{13}^{+z}+A_{14}^{+z}-D^{+z}\right)}{A_{13}^{zz}-A_{14}^{zz}+D^{zz}-2\,\omega_{e1}}+\frac{A_{23}^{--}\left(-A_{13}^{+z}-A_{14}^{+z}-D^{+z}\right)}{A_{13}^{zz}+A_{14}^{zz}+D^{zz}+2\,\omega_{e1}}-\frac{A_{13}^{+-}\left(A_{23}^{-z}-A_{24}^{-z}-D^{z-}\right)}{A_{23}^{zz}-A_{24}^{zz}-D^{zz}-2\,\omega_{e2}}-\frac{2\left(\frac{A_{13}^{z-}}{2}-\frac{A_{23}^{z-}}{2}+\frac{d^{-z}}{2}\right)D^{+-}}{A_{13}^{zz}+A_{14}^{zz}-A_{23}^{zz}-A_{24}^{zz}+2\,\omega_{e1}-2\,\omega_{e2}}+\frac{A_{13}^{+-}\left(A_{23}^{-z}+A_{24}^{-z}-D^{z-}\right)}{A_{23}^{zz}+A_{24}^{zz}-D^{zz}+2\,\omega_{e2}}-\frac{2\left(-\frac{A_{13}^{z-}}{2}+\frac{A_{23}^{z-}}{2}+\frac{d^{-z}}{2}\right)D^{+-}}{A_{13}^{zz}-A_{14}^{zz}-A_{23}^{zz}+A_{24}^{zz}-2\,\omega_{e1}+2\,\omega_{e2}}-\frac{\left(A_{13}^{z-}-A_{23}^{z-}+d^{-z}\right)D^{+-}}{A_{13}^{zz}-A_{23}^{zz}+d^{zz}-2\,\omega_{n}}-\frac{2\,A_{23}^{--}\left(\frac{A_{13}^{+z}}{2}+\frac{A_{14}^{+z}}{2}+\frac{D^{+z}}{2}\right)}{A_{13}^{zz}+A_{24}^{zz}+d^{zz}+D^{zz}+2\,\omega_{e2}-2\,\omega_{n}}+\frac{\left(A_{13}^{z-}-A_{23}^{z-}-d^{-z}\right)D^{+-}}{A_{13}^{zz}-A_{23}^{zz}-d^{zz}+2\,\omega_{n}}+\frac{2\,A_{13}^{+-}\left(\frac{A_{23}^{-z}}{2}+\frac{A_{24}^{-z}}{2}-\frac{D^{z-}}{2}\right)}{A_{14}^{zz}+A_{23}^{zz}-d^{zz}-D^{zz}+2\,\omega_{e1}+2\,\omega_{n}}-\frac{2\,A_{13}^{+-}\left(-\frac{A_{23}^{-z}}{2}+\frac{A_{24}^{-z}}{2}+\frac{D^{z-}}{2}\right)}{A_{14}^{zz}-A_{23}^{zz}-d^{zz}+D^{zz}+2\,\omega_{e1}+2\,\omega_{n}}-\frac{2\,A_{23}^{--}\left(-\frac{A_{13}^{+z}}{2}+\frac{A_{14}^{+z}}{2}-\frac{D^{+z}}{2}\right)}{A_{13}^{zz}-A_{24}^{zz}-d^{zz}+D^{zz}-2\,\omega_{e2}+2\,\omega_{n}}\right)$$

In[∘]:= **HamM⟦3, 3⟧ - HamM⟦2, 2⟧**

••• Part: Part specification HamM⟦2, 2⟧ is longer than depth of object.

••• Part: Part specification HamM⟦3, 3⟧ is longer than depth of object.

In[∘]:= **H0M⟦2, 2⟧ - H0M⟦3, 3⟧**
**H0M⟦4, 4⟧ - H0M⟦3, 3⟧**

Out[∘]=

$$\frac{A_{13}^{zz}}{2}-\frac{A_{14}^{zz}}{2}+\frac{A_{23}^{zz}}{2}-\frac{A_{24}^{zz}}{2}$$

Out[∘]=

$$\frac{A_{13}^{zz}}{2}+\frac{A_{23}^{zz}}{2}+\frac{d^{zz}}{2}+\omega_{n}$$
